# Detection of copy number variants in African goats using whole genome sequence data

**DOI:** 10.1186/s12864-021-07703-1

**Published:** 2021-05-29

**Authors:** Wilson Nandolo, Gábor Mészáros, Maria Wurzinger, Liveness J. Banda, Timothy N. Gondwe, Henry A. Mulindwa, Helen N. Nakimbugwe, Emily L. Clark, M. Jennifer Woodward-Greene, Mei Liu, the VarGoats Consortium, George E. Liu, Curtis P. Van Tassell, Benjamin D. Rosen, Johann Sölkner

**Affiliations:** 1 University of Natural Resources and Life Sciences, Vienna, Austria; 2 Lilongwe University of Agriculture and Natural Resources, Lilongwe, Malawi; 3 National Livestock Resources Research Institute, Tororo, Uganda; 4 Kyambogo University, Kyambogo, Uganda; 5 The Roslin Institute, University of Edinburgh, Edinburgh, Scotland UK; 6 Animal Genomics and Improvement Laboratory, USDA-ARS, Beltsville, MD USA; 7 National Agricultural Library, USDA-ARS, Beltsville, MD USA

**Keywords:** African goats, Copy number variations, Whole genome sequence

## Abstract

**Background:**

Copy number variations (CNV) are a significant source of variation in the genome and are therefore essential to the understanding of genetic characterization. The aim of this study was to develop a fine-scaled copy number variation map for African goats. We used sequence data from multiple breeds and from multiple African countries.

**Results:**

A total of 253,553 CNV (244,876 deletions and 8677 duplications) were identified, corresponding to an overall average of 1393 CNV per animal. The mean CNV length was 3.3 kb, with a median of 1.3 kb. There was substantial differentiation between the populations for some CNV, suggestive of the effect of population-specific selective pressures. A total of 6231 global CNV regions (CNVR) were found across all animals, representing 59.2 Mb (2.4%) of the goat genome. About 1.6% of the CNVR were present in all 34 breeds and 28.7% were present in all 5 geographical areas across Africa, where animals had been sampled. The CNVR had genes that were highly enriched in important biological functions, molecular functions, and cellular components including retrograde endocannabinoid signaling, glutamatergic synapse and circadian entrainment.

**Conclusions:**

This study presents the first fine CNV map of African goat based on WGS data and adds to the growing body of knowledge on the genetic characterization of goats.

**Supplementary Information:**

The online version contains supplementary material available at 10.1186/s12864-021-07703-1.

## Background

Structural variations (SV) are an important source of genetic variation [1–4]. SV are generally considered to comprise a myriad of subclasses that consist of unbalanced copy number variants (CNV), which include deletions, duplications and insertions of genetic material, as well as balanced rearrangements, such as inversions and interchromosomal and intrachromosomal translocations [5]. Deletions and insertions are referred to as unbalanced SV because they result in changes in the length of the genome. Insertions or deletions in the genome are typically considered CNV when they are at least 50–1000 base-pairs (bp) long [6–11]. CNV are not as abundant as single nucleotide polymorphisms (SNP), but because of their larger sizes, they may have a dramatic effect on gene expression in individuals [12]. Duplication or deletion in or near a gene or the regulatory region of the gene may lead to modification of the function of the gene.

CNV cover about 4.5–9.8% of the human genome [13] and are associated with many Mendelian disorders [12]. Girirajan et al. [14] found that CNV significantly determine the severity and prognosis of many genetic disorders. Approximately 14% of diseases in children with intellectual disability are caused by CNV [15]. On the other hand, some CNV have been found to be associated with adaptive fitness of individuals, such as adaptation to starch diets associated in the gene encoding α-amylase [13].

Traditionally, microarray-based comparative genomic hybridization (array CGH) or SNP genotyping arrays are used to detect CNV. Several studies have been carried out using these methods to detect and map CNV in the goat genome, including studies by Fontanesi et al. [16] in four goat breeds; Nandolo et al. [17] in 13 East African goat breeds; and Liu et al. [18] in the global goat population.

Detecting CNV using array CGH and SNP genotyping arrays suffers from shortcomings that include hybridization noise, limited coverage of the genome, low resolution, and difficulty in detecting novel and rare mutations [19–21]. The development of whole-genome sequencing (WGS) technologies has made it possible for more rigorous and accurate detection of CNV.

According to Mills et al. [22], WGS-based CNV detection methods fall into four major approaches: methods based on paired-end (PE) mapping, split reads (SR), read depth (RD) and de novo assembly of a genome (AS). The PE and SR methods are useful for detection of small-scale CNV [23], and several algorithms are loosely based on them, including BreakDancer [24], Pindel [25], and Delly [26]. RD approaches are very useful for detection of larger CNV. Algorithms using this approach include CNV-Seq [27], CNVnator [28] and the event-wise testing approach (EWT) developed by Yoon et al. [29]. The methods can also be combined. For example, LUMPY [30] is able to combine two or more of the previous approaches to refine SV detection. Assembly-based approaches are computationally intensive and are therefore not generally used with WGS data [23, 31]. Most of these SV-detection algorithms have been extensively reviewed [1, 31–34].

LUMPY implements a breakpoint prediction framework, where a breakpoint is defined as a pair of genomic regions that are adjacent in a sample, but not in the reference genome. The location of the breakpoint is determined using a probability function that considers different sources of evidence supporting the existence of a breakpoint, including information from discordant read pairs and split reads. A discordant read pair occurs when sequence from two ends of an insert are inconsistent when compared to the reference genome. These inconsistencies result from differences between mapping distance or the orientation between the pairs of sequences [35, 36]. Split reads are sequences that map to the reference genome on one end only, and, as explained by Ye and Hall [33], such reads can indicate the location of a breakpoint with a high degree of certainty. There are similar algorithms that rely heavily on the use of breakpoints to determine genome rearrangements at single-nucleotide resolution, including Delly [26] and Pindel [25].

Like LUMPY, Manta [37] incorporates use of PE and SR methods. However, Manta also uses AS analysis. Manta overcomes the computational expense of AS methods by splitting the work into many smaller workflows which can be carried out in parallel. Manta scans the genome for SV and then scores, genotypes and filters the SV based on diploid germline and somatic biological models [37]. Manta can detect all structural variant types that are identifiable in the absence of copy number analysis and large-scale de-novo assembly, which is why this approach is also a good candidate for joint analysis of small sets of diploid individuals, tumor samples, and similar analyses. Both LUMPY and Manta are good at identifying SV break points with high resolution.

Many studies have been carried out to detect CNV using WGS data in various domesticated species: cattle [38], cats [39], chickens [40], dogs [41], etc. So far, there is no report of goat CNV discoveries using WGS data. The goal of this study was to identify CNV in the goat genome through the intersection of LUMPY and Manta outputs as a part of the characterization of African goats in conjunction with the ADAPTmap project [42]. Goats are a very important farm animal genetic resource for the livelihoods of African smallholders, and a deeper understanding of the goat genome is necessary to facilitate the improvement of goats in the region. This study aimed to generate a fine-scale CNV map for the goat genome.

## Results

### Number and distribution of CNV

The number of CNV detected depended on the filter levels (low, medium, or stringent) and the cut-off point for CNV length (3 Mb or 10 Mb) as given in Supplementary Figure 11 (Additional file 2). Using precise SV only with moderate filters (PE + SR ≥ 5), LUMPY detected 8563 duplications and 230,497 deletions while Manta detected 24,088 duplications and 320,374 deletions. A combined data set with 244,876 deletions and 8677 duplications (totaling 253,553, translating into an average of 1393 CNV per animal) was derived from the intersection of the LUMPY and Manta sets after removal of variants shorter than 50 bp or longer than 3 Mb. The combined data set had more observations than the LUMPY data set (which had fewer raw CNV) because for some individuals, many short CNV from Manta intersected with few long CNV from LUMPY.

**Fig. 1:**
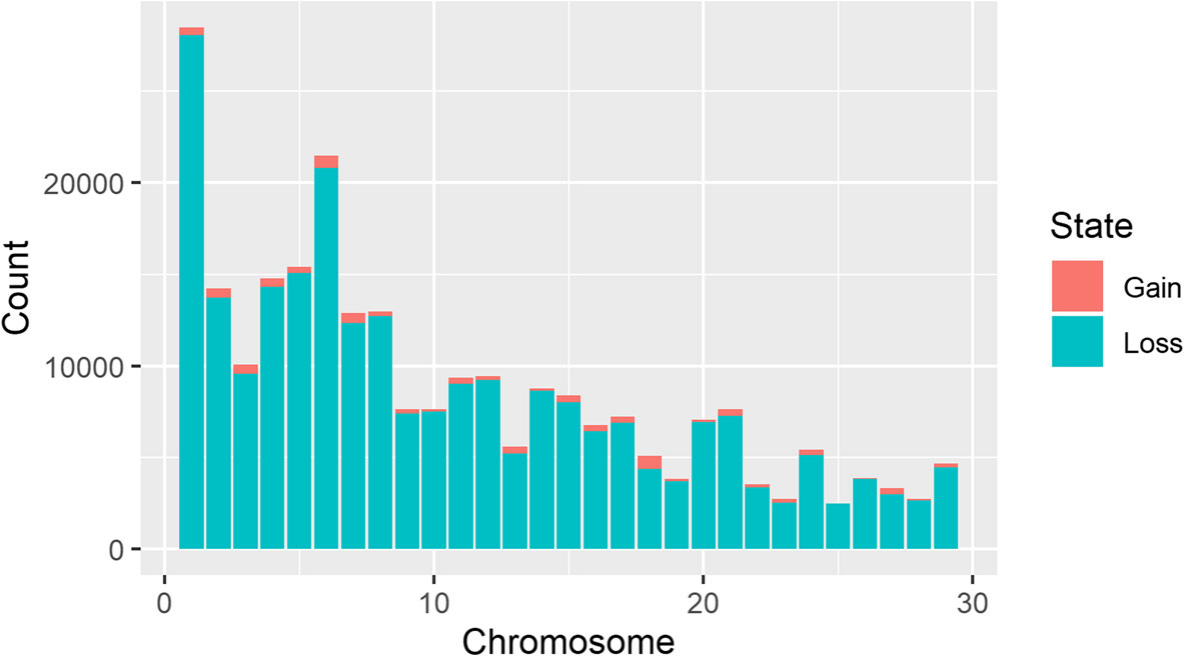
Overall numbers of CNV by chromosome and CNV state. Orange is for copy gain and blue-green is for copy loss

The CNV were distributed across the 29 autosomes as shown in Fig. 1. A vast majority of the CNV (96.6%) were losses. This is not unexpected, because all CNV detection methods suffer from an inherent deficiency in detecting insertions. In the case of CNV detection using WGS data, this limitation is even more pronounced with PE methods, because they detect insertions when the mapped reads are at a distance shorter than the fragment length, so they are not able to detect insertions larger than the insert size of the reference library [43]. This has also been supported by the observation that recall percentage is lower than 2 and 5% for medium (1–100 kb) and large (100 kb-1 Mb) duplications, respectively, for most of the SV-calling algorithms currently in use, including Manta and LUMPY used in this study [44].

**Fig. 2:**
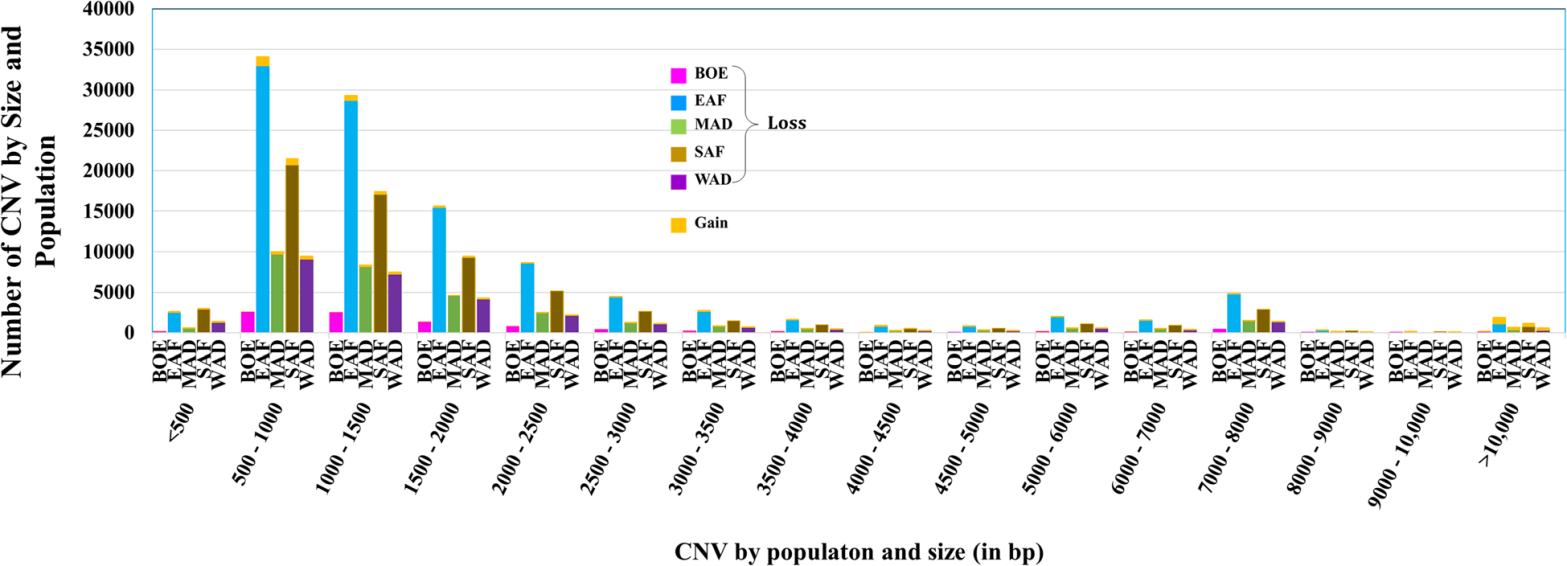
Distribution of the sizes of CNV for each population by CNV state. Orange is for copy gains while the rest of the colors for copy loss for each of the five populations (magenta for Boer; blue is for the East African; green for Madagascar; brown for Southern African and purple for West African)

**Fig. 3:**
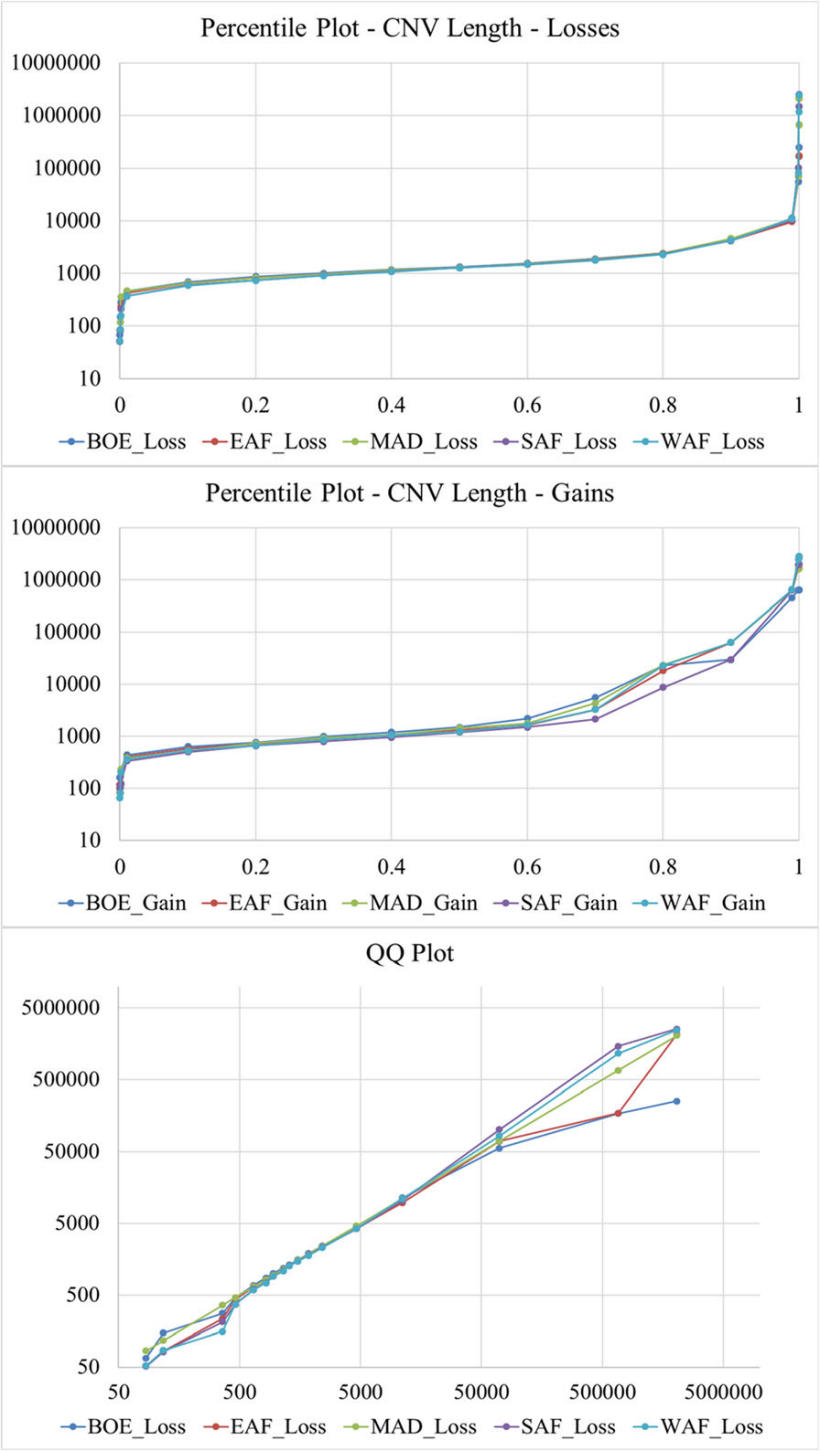
Percentile plots for CNV gains and losses and a QQ plot for CNV losses

Overall, the mean CNV length was about 3.3 kb, with a median of 1.3 kb. The distribution of the lengths of the CNV for each population are shown in Fig. 2 by CNV length category. A summary of the descriptive statistics of the CNV for the populations are given in Table 1. Most of the CNV losses (99.92%) were less than 100 kb long while 6.3% of CNV gains were longer than 100 kb. Despite the overwhelming proportion of losses over gains, there were more CNV gains observed over 100 kb than losses. Similarly, only 1.04% of the loss CNV were longer than 10 kb, while almost one-quarter (22.99%) of all gain CNV were over 10 kb. As a result, CNV gains were longer than CNV losses and had larger range in length. Deletions and duplications averaged about 2.3 and 31.5 kb long, with median lengths of 1.3 and 1.4 kb, respectively. There were no significant differences in the distribution of CNV across the five populations as shown in the percentile and sample QQ plots in Fig. 3.

**Table 1:** Descriptive statistics of CNV and CNV length for each population

Population	Number of samples	CNV		CNV length (bp)			
		State	Number	Mean	Median	Minimum	Maximum
BOE	9	Loss	9079	2227.1	1326	67	254,129
		Gain	331	20,165.9	1500	161	631,262
		Overall	9410	2858.1	1330	67	631,262
EAF	80	Loss	108,051	2244.7	1293	52	2,161,018
		Gain	3544	30,979.2	1316.5	118	2,777,398
		Overall	111,595	3157.2	1293	52	2,777,398
MAD	27	Loss	31,426	2475.3	1295	84	2,069,909
		Gain	1078	28,384.1	1446	84	1,660,243
		Overall	32,504	3334.6	1296	84	2,069,909
SAF	44	Loss	67,099	2368.9	1285	51	2,539,701
		Gain	2514	31,000.7	1192	101	1,959,154
		Overall	69,613	3402.9	1283	51	2,539,701
WAF	22	Loss	29,221	2491.4	1280	52	2,457,795
		Gain	1210	40,255.3	1234	65	2,788,546
		Overall	30,431	3993	1280	52	2,788,546

### Population CNV differentiation

**Fig. 4:**
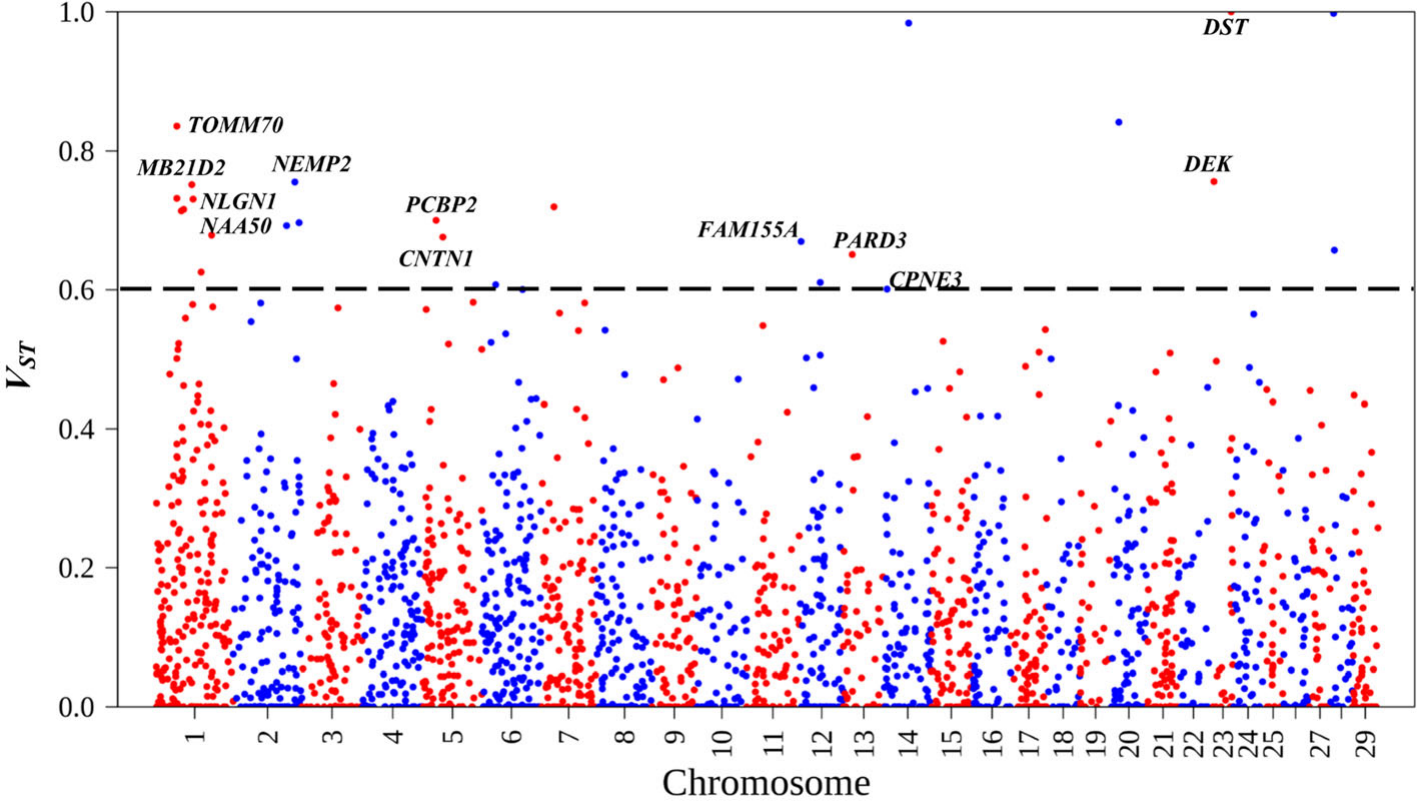
Population CNV differentiation, estimated by V_ST_ computed across all populations, plotted for each chromosome. The dotted line represents the V_ST_ threshold value for this test (0.601)

Analysis of population differentiation (*V*_*ST*_) as described by Redon et al. [11] showed that several CNV were highly differentiated between and across the populations. Some of these CNV overlapped with genes of importance in goats. Results for the pairwise population *V*_*ST*_ tests and the *V*_*ST*_ test across all the populations with their respective 99th percentile CNV *V*_*ST*_ thresholds are given in Supplementary Table 1 (Additional file 1). *V*_*ST*_ values for the pairwise tests are given in Supplementary Figures 1–10 (Additional file 2). The *V*_*ST*_ values for genes that were in CNV that were highly differentiated across all populations are shown in Fig. 4. The gene *DST* was in a CNV with a very high *V*_*ST*_ threshold across all the populations. *DST* has been associated with herpes virus and respiratory disease (BRD) in cattle [45]. Some CNV were highly differentiated both between and across populations. CNV with high differentiation between only some populations include the CNV corresponding to the genes *BCO2*, *CCSER1 (FAM190A)*, *COL24A1*, *CPNE4*, *CWC22*, *IMMP2L*, *KBTBD12*, *LAMA3*, *NAALADL2*, *RFX3*, *SEMA3D*, *SLC2A13*, *STPG2 (C4orf37)*, *TAFA2 (FAM19A2)*, *TMEM117*, *TMEM161B* and *VPS13B*. The rest of the genes were in CNV that were highly differentiated across all populations.

### Number and distribution of CNV regions (CNVR)

The lists of CNV regions (CNVR) by population are given in Supplementary Table 2 (Additional file 1) and their locations on the goat genome are shown in Fig. 5. Plots of the CNVR for each breed (with more than 2 animals) are given in Supplementary Figures 12 to 40 (Additional file 2). Descriptive statistics of the CNVR for each population are given in Supplementary Table 3 (Additional file 1) while a distribution of CNVR by size and populations is given in Fig. 6. Over 92% of the CNVR were copy losses. There was a wide variation in the number and sizes of the CNVR between and among the populations. The fraction of copy gains or gains and losses was highest in the group of CNVR of at least 10 kbp, with 25% copy gains and 19% for losses/gains (Fig. 6).

**Fig. 5:**
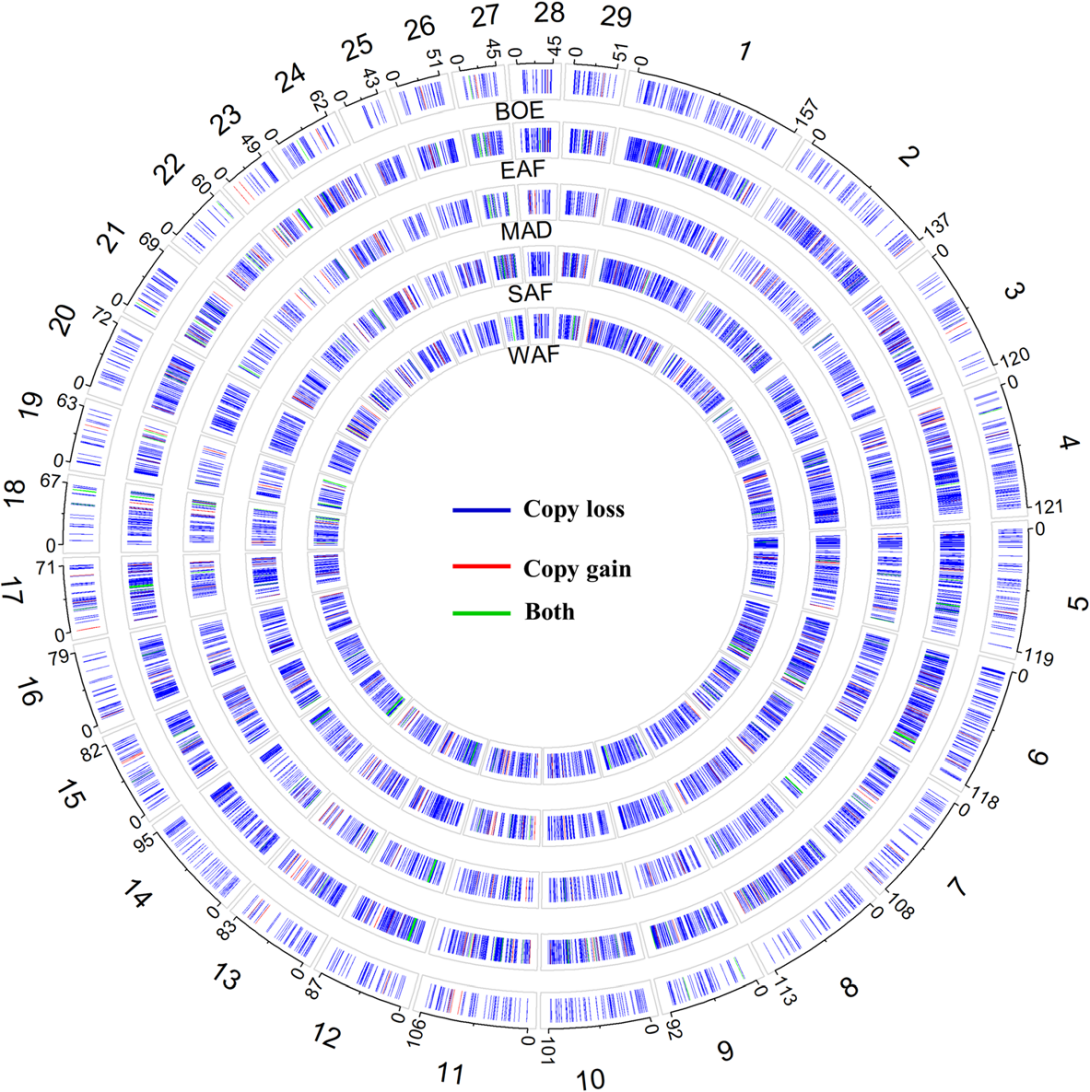
Location of the CNVR for the 29 autosomes by population. The outermost numbers are the autosomes, and the other numbers are the start and end positions of each autosome

**Fig. 6:**
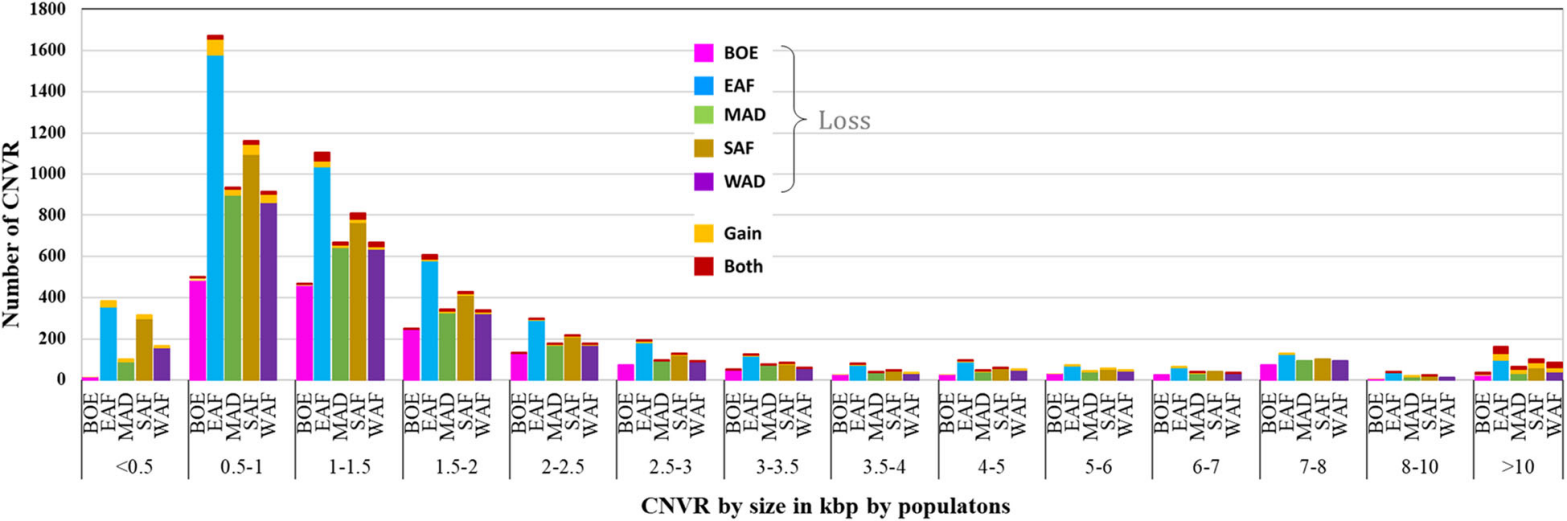
Distribution of size of CNVR (in kbp) for each population. Orange is for copy gains and red is for CNVR with both copy gains and losses. The rest of the colours for copy loss for each of the five populations (magenta for Boer; blue is for the East African; green for Madagascar; brown for Southern African and purple for West African)

### Number and distribution of global CNVR

**Fig. 7:**
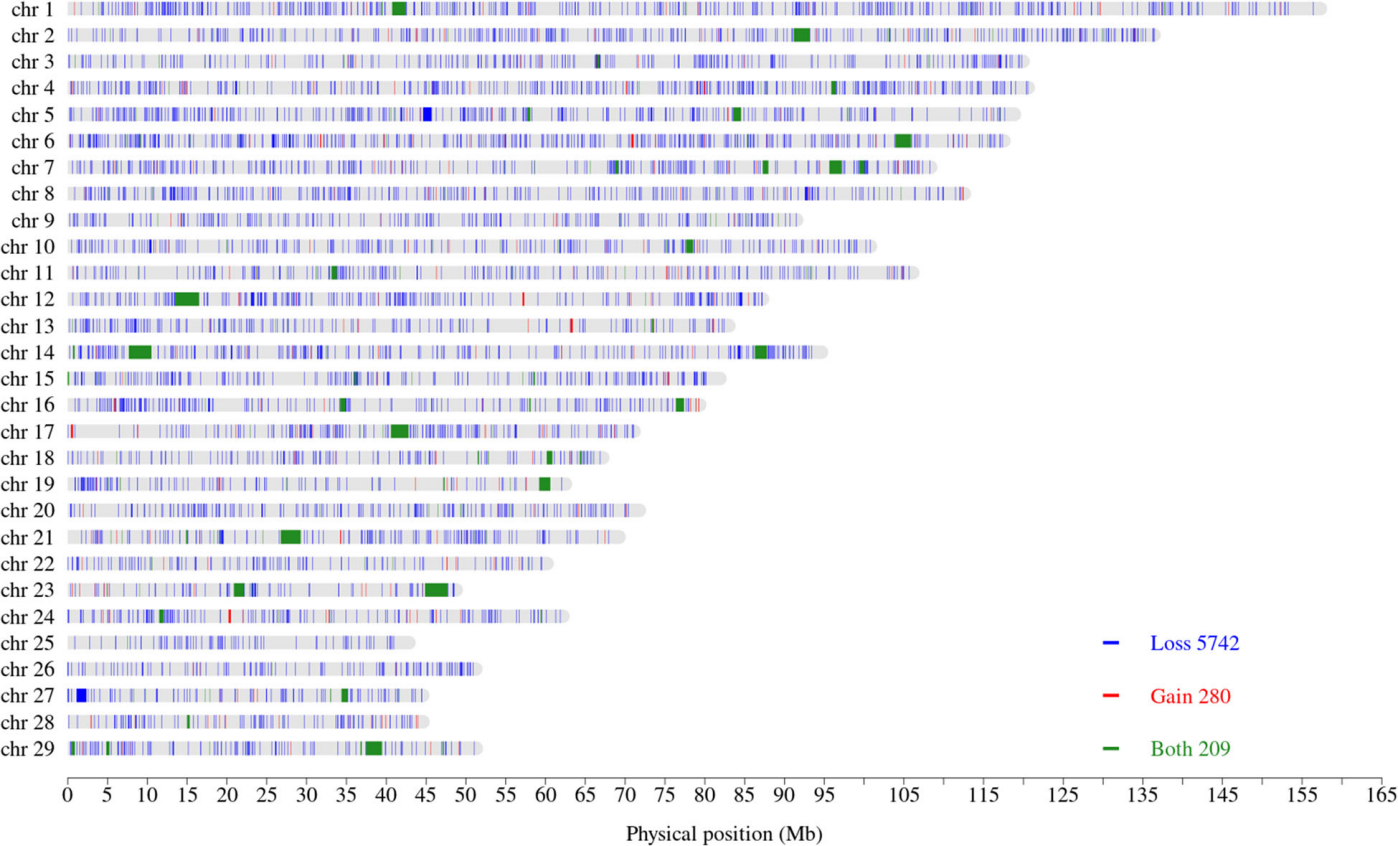
Location of the global CNVR across the 29 autosomes. Blue is for loss; red is for gain and green is for both loss and gain

Global CNVR for different levels of SV filter parameters are given in Supplementary Figures 41 to 64 (Additional file 2). Only the PE and SR filter levels and the CNV length cut-off point affected CNVR coverage. Inclusion of imprecise SV led to an increase in the proportion of called duplications, but the additional duplications were much longer than the upper cut-off point for CNV length. A total of 6231 global CNVR were found across all animals. A list of the global CNVR is given in Supplementary Table 4 (Additional file 1) and a summary is given in Table 2. There were 5742 CNVR with copy losses, 280 with copy gains and 209 with both copy losses and gains in different individuals. The locations of the global CNVR are given in Fig. 7. CNVR with both gains and losses were much longer (mean 185.8 kb) and constituted a significant proportion of the total CNVR coverage (65.6%). Sixteen of these were longer than 1 Mb (on chromosomes 1, 2, 6, 7, 12, 14 (two regions), 17, 19, 21, 23 (two regions), 27 and 29).

**Table 2:** CNVR summary statistics for each CNV state based on CNV occurring in at least 2 individuals

Copy state	Number of CNVR	Length (bp)				CNVR coverage (bp)
		Mean	Median	Minimum	Maximum	
Loss	5742	3041.3	1140.5	52	1,177,087	17,463,236
Gain	280	10,377.9	1008.0	302	236,347	2,905,806
Both	209	185,755.2	1731.0	616	2,956,746	38,822,839
**Overall**	6231	9499.6	1157.0	52	2,956,746	59,191,881

Overall, the CNVR covered about 59.2 Mb of the goat genome. Previous work on genome-wide CNV discovery in goats using SNP data done by Liu et al. [18] showed that CNVR cover approximately 262 Mb of the goat genome. Of the 978 CNVR reported in that study, 540 CNVR intersected with 819 CNVR identified in our study. The amount of the overlap between the CNVR in the two studies was 217.1 Mb, covering 38.6 Mb (65.1%) in this study, and 194.2 Mb (74.1%) in the other study.

### Common and rare CNVR

**Fig. 8:**
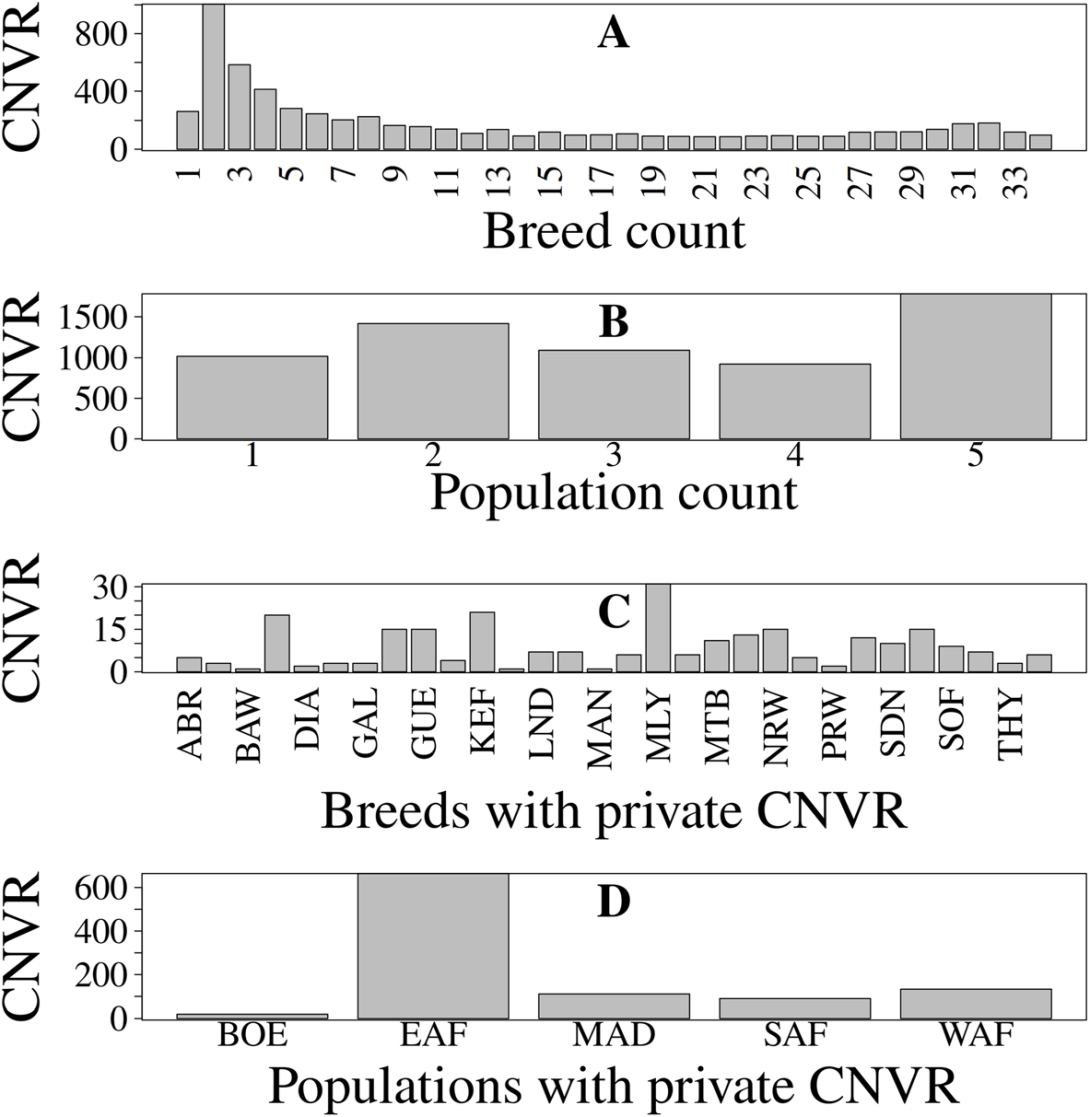
Distribution of the CNVR. **a**, **b** Number of CNVR found in different numbers of breeds and populations, respectively. **c**, **d** Distribution of CNVR found in only a single breed and only a single population only, respectively. In C, only 30 breeds had private CNVR

Most of the CNVR (> 95.9%) were found in at least 2 breeds. Out of the 6231 CNVR, 98 (1.6%) were present in all the 34 breeds and 1790 (28.7%) were present in all the populations (Fig. 8a and b). The most frequent CNVR observed was on chromosome 6 from 115,822,332 bp to 115,825,687 bp with a frequency of 96.2%. There were 259 CNVR private to 30 breeds, and 1018 private to all 5 populations, distributed as shown in Fig. 8c and Fig. 8d. BOE (Tanzania and Zimbabwe), KEF (Ethiopia) and MLY (Tanzania) breeds had the highest numbers of private CNVR (20, 21 and 31, respectively).

### Functional annotation and gene enrichment analysis

Functional annotation was carried out for genes in global and private CNVR. Up to 2980 genes overlapped with the 6321 CNVR identified in this study. Up to 755 of these genes formed 24 clusters, with enrichment scores ranging from 0.0 to 1.89. Higher enrichment scores imply higher overrepresentation of the genes in the gene set for the gene enrichment term [46]. The top 3 clusters with the highest enrichment scores are given in Table 3 while the full list is given in Supplementary Table 5 (Additional file 1). The most significant GO terms identified in the analysis included retrograde endocannabinoid signaling; glutamatergic synapse; circadian entrainment; dopaminergic synapse; gastric acid secretion; long-term potentiation; salivary secretion; and calcium signaling pathway.

**Table 3:** Functional annotation clusters of the genes found in the global CNVR based on analysis in DAVID

Cluster (Enrichment score, database)	Enrichment term	Gene count	*p*-value
1 (1.89, KEGG_PATHWAY)	chx04020: Calcium signaling pathway	31	0.001
	chx04970: Salivary secretion	16	0.011
	chx04022: cGMP-PKG signaling pathway	25	0.016
	chx04270: Vascular smooth muscle contraction	19	0.037
	chx04261: Adrenergic signaling in cardiomyocytes	21	0.046
2 (1.62, KEGG_PATHWAY)	chx04723: Retrograde endocannabinoid signaling	24	0.000
	chx04724: Glutamatergic synapse	24	0.000
	chx04713: Circadian entrainment	21	0.001
	chx04728: Dopaminergic synapse	25	0.002
	chx04971: Gastric acid secretion	16	0.004
	chx04720: Long-term potentiation	14	0.009
	chx04970: Salivary secretion	16	0.011
	chx04925: Aldosterone synthesis and secretion	15	0.014
	chx04730: Long-term depression	13	0.014
	chx04540: Gap junction	16	0.019
	chx04750: Inflammatory mediator regulation of TRP channels	18	0.020
	chx04921: Oxytocin signaling pathway	23	0.028
	chx04922: Glucagon signaling pathway	17	0.028
	chx04972: Pancreatic secretion	16	0.033
	chx04270: Vascular smooth muscle contraction	19	0.037
	chx04725: Cholinergic synapse	18	0.043
	chx04911: Insulin secretion	14	0.053
	chx04726: Serotonergic synapse	17	0.072
	chx04915: Estrogen signaling pathway	15	0.091
	chx04961: Endocrine and other factor-regulated calcium reabsorption	8	0.094
	chx04912: GnRH signaling pathway	13	0.131
	chx04918: Thyroid hormone synthesis	11	0.137
	chx04924: Renin secretion	10	0.161
	chx04611: Platelet activation	16	0.273
	chx04916: Melanogenesis	11	0.458
	chx04310: Wnt signaling pathway	14	0.599
3 (1.14, KEGG_PATHWAY)	chx05204: Chemical carcinogenesis	12	0.033
	chx00980: Metabolism of xenobiotics by cytochrome P450	10	0.075
	chx00982: Drug metabolism - cytochrome P450	10	0.075
	chx00830: Retinol metabolism	9	0.152

CNVR private to populations and breeds overlapped with 172 and 620 genes, respectively. The GO terms associated with these genes based on functional analysis are listed in Supplementary Table 6 (Additional file 1). The genes that overlapped with the CNVR private to breeds were not significantly enriched in biological processes, molecular functions and cellular components, while the ones that overlapped with the CNVR private to populations were significantly enriched (*P* ≤ 0.05) with such terms as aldosterone synthesis and secretion; glucagon signaling pathway; insulin secretion; glutamatergic synapse; thyroid hormone synthesis; gastric acid secretion and phosphatidylinositol signaling system. The most common CNVR (chr6:115,822,332-115,825,687) includes the gene TMEM129 (transmembrane protein 129) that has been reported to be responsible for ubiquitination and proteasome-mediated degradation of misformed or unassembled proteins in the cytosol [47–49], and belongs to a network responsible for cellular assembly and organization, cellular function and maintenance, and cell cycle [50].

## Discussion

This study identified CNV and CNVR in the goat genome using WGS data. Use of WGS for CNV detection is highly encouraged, because it overcomes many of the shortcomings of the other CNV detection methods such as the ones using array CGH and SNP data [19–21]. Genome-wide studies to discover CNV have already been done in other domesticated species, such as in *Sus scrofa* [51], *Bos taurus* [38, 52] and *Felis catus* [39]. Here we provide a first glimpse of the goat genome CNV map at a dense genome coverage, using animals from 34 diverse breeds from the African continent. This addition is an important contribution, as goats are an important source of income and high-quality animal protein for small holder farmers in Africa.

We used two software suites (LUMPY [30] and Manta [37]) for detecting SV to increase our confidence in the SV calls. Both software packages use split read and read-pair methods. They complement each other in that LUMPY makes use of read depth methods, while Manta draws heavily on genome assembly methods. Taking the intersection of SV calls from the two methods gives us confidence that the number of false positives in the SV calls was kept to a minimum, although this means that some true SV were possibly filtered out.

This study has shown that there are wide variations in the number and sizes of CNV in the goat genome between chromosomes, individuals and breeds. However, considering the small and variable numbers of samples within breeds, breed comparisons are not particularly meaningful. The results suggest that there are negligible differences in the sizes of CNV between populations. Some of the CNV displayed large differences between populations, suggestive of population-specific selective pressures.

A large proportion of the global CNVR identified in this study (65.1%) are within the CNVR reported by Liu et al. [18]. The remaining 34.9% may comprise false positive CNVR and CNVR that were missed by the PennCNV algorithm used in the other study, considering the limitation of CNV detection using SNP data, which include limited coverage for genome, low resolution, and difficulty in detecting novel and rare mutations. The CNVR coverage of 2.4% (59.2 Mb of about 2466 Mb of autosomal genome) found in this study is lower than the 4.8–9.5% SV coverage in the human genome [13], comparable to 55.6 Mb (2.0%) reported for cattle [38], later revised to 87.5 Mb (3.1%) [53].

*V*_*ST*_ analysis showed that several CNV were highly differentiated among and across the populations. The genes in the highly differentiated CNV included *BCO2* (Madagascar vs West African population differentiation), *CCSER1 (FAM190A)* (Boer vs East African), *FAM155A* (across all populations), *GNRHR* (Boer vs Madagascar; Boer vs West African), *IMMP2L* (East vs Southern African), *LAMA3* (East African vs Madagascar), *NAALADL2* (East vs Southern African), *TAFA2 (FAM19A2)* (East vs Southern African) and *TOMM70* (across all the populations)*.* Våge and Boman [54] reported that *BCO2* is associated with the accumulation of carotenoids in the adipose tissue of sheep, leading to the yellow fat syndrome. The quality of semen (including total sperm motility, average path velocity and beat cross frequency) in Holstein-Friesian bulls has been associated with *CCSER1 (FAM190A)* as well as *FAM155A* [55]. *GNRHR* has been associated with number of days to first service after calving in dairy cattle [56] while *IMMP2L* is associated with cow conception rate [57]. The partial deletion of *LAMA3* is responsible for epidermolysis bullosa in horses [58]; *NAALADL2* is believed to be responsible for immune homeostasis [59], and *TAFA2 (FAM19A2)* is believed to be responsible for the regulation of feed intake and metabolic activities in mice [60]. Yamano et al. [61] reported that *TOMM70* is responsible for integral mitochondrion proteins and for metabolism.

Functional annotation and clustering analysis revealed that the CNVR identified in the study have genes that are significantly enriched with many biological processes, molecular functions and cellular components, some of the most significant of which are retrograde endocannabinoid signaling, circadian entrainment and long-term potentiation. The retrograde endocannabinoid signaling system is a complex and diverse regulator of synaptic function [62], and is responsible for many diseases in the nervous system and peripheral organs. In the human genome, this system is widely considered as a potential target for treating conditions such as alcoholism [63]. A CNVR in the cannabinoid receptor 2 (CNR2) region has been reported in the human genome, but its effect has not been fully characterized [64]. Zajkowska et al. [65] suggested that there is need to explore genetic variation in the system from the perspective of copy number of variations.

Circadian entrainment is an important aspect of animal behavior and adaptation, especially considering the wide range of environmental conditions the animals are exposed to. An example of goat adaptation to the environment is their ability to rapidly change the size of their foreguts in response to changes in the environment [66]. Goats tend to be active during some parts of the day only [67], and this varies with season [67], suggesting a considerable amount of circadian entrainment. The increased importance of the biological process “response to stimulus” (GO:0050896) in the highly differentiated CNV may also support the hypothesis of the importance of circadian entrainment in goats.

## Conclusions

This study presents the first fine CNV map of the African goats based on WGS data. This information will prove invaluable for further improvement of goats, especially on African continent, as more phenotype data becomes available, through CNV or CNVR association analyses and other approaches.

## Methods

### Sample description

The data used in this study was generated from 182 goats representing 34 breeds from 9 Sub-Saharan African countries (Ethiopia, Kenya, Madagascar, Malawi, Mali, Mozambique, Tanzania, Uganda, and Zimbabwe), and these countries were grouped into four populations based on geographic locations and a fifth population of Boer goats obtained in Tanzania and Zimbabwe. The Boer goat is a special breed widely used in Africa and much of the world [68]. The samples were previously genotyped using the Illumina Goat SNP50 BeadChip [69] as described by Bertolini et al. [70], Cardoso et al. [71] and Colli et al. [72], and some of them were also used for detection of CNV using 50 K SNP chip data, as reported by Liu et al. [18]. A list of the breeds, populations and samples sizes used in the analysis is given in Table 4.

**Table 4:** List of the breeds used in the analysis

Population	Population Code	Breed name	Breed Code	Country	Number of samples
Boer	BOE	Boer	BOE	Tanzania	2
Boer	BOE	Boer	BOE	Zimbabwe	7
East African	EAF	Abergelle	ABR	Ethiopia	6
East African	EAF	Galla	GAL	Kenya	7
East African	EAF	Gogo	GOG	Tanzania	7
East African	EAF	Gumez	GUM	Ethiopia	4
East African	EAF	Keffa	KEF	Ethiopia	7
East African	EAF	Landin	LND	Mozambique	5
East African	EAF	Maasai	MAA	Tanzania	7
East African	EAF	Manica	MAN	Mozambique	3
East African	EAF	Malya	MLY	Tanzania	7
East African	EAF	Norwegian	NRW	Tanzania	3
East African	EAF	Pare White	PRW	Tanzania	6
East African	EAF	Saanen	SAA	Tanzania	4
East African	EAF	Small East African	SEA	Kenya	7
East African	EAF	Small East African	SEA	Mozambique	6
East African	EAF	Sonjo	SNJ	Tanzania	2
East African	EAF	Woyito Guji	WYG	Ethiopia	7
Madagascar	MAD	Androy	AND	Madagascar	4
Madagascar	MAD	Diana	DIA	Madagascar	3
Madagascar	MAD	Menabe	MEN	Madagascar	7
Madagascar	MAD	Sofia	SOF	Madagascar	6
Madagascar	MAD	SudOuest	SOU	Madagascar	7
Southern African	SAF	Balaka-Ulongwe	BAW	Malawi	2
Southern African	SAF	Dedza	DZD	Malawi	4
Southern African	SAF	Lilongwe	LGW	Malawi	3
Southern African	SAF	Mashona	MSH	Zimbabwe	7
Southern African	SAF	Matebele	MTB	Zimbabwe	7
Southern African	SAF	Nsanje	NSJ	Malawi	6
Southern African	SAF	Thyolo	THY	Malawi	7
West African	WAF	Guerra	GUE	Mali	6
West African	WAF	Maure	MAU	Mali	1
West African	WAF	Naine	NAI	Mali	5
West African	WAF	Peulh	PEU	Mali	1
West African	WAF	Soudanaise	SDN	Mali	7
West African	WAF	Targui	TAR	Mali	2

Sample processing was done by Edinburgh Genomics using the Edinburgh Clinical Genomics method. This approach uses Illumina SeqLab products and services, including, Illumina TruSeq library preparation, Illumina cBot2 cluster generation, Illumina HiSeqX sequencing, Hamilton Microlab STAR integrative automation, and Genologics Clarity LIMS X Edition as outlined in Supplementary Table 7 (Additional file 1). Quality control information for the samples is given in Supplementary Table 8 (Additional file 1).

### Sequence alignment

Sequence alignment was done using the Burrow-Wheeler Alignment (BWA) tool version 0.7.13-r1126 with the maximal exact matches (MEM) “mem” option [73]. The reads were aligned to the ARS1 *Capra hircus* (goat) reference assembly (https: //www.ncbi.nlm.nih.gov/assembly/GCF_001704415.1/) [74]. The aligned reads were processed into binary sequence alignment map (BAM) format using SAMTools version 1.8 [75].

### Detection of SV

SV were detected using LUMPY version 0.2.13–85-gc1bcea1 and Manta version 1.5.1, which are two of the most used algorithms for detecting SV. In LUMPY, the “lumpyexpress” script was used. This script runs automated breakpoint detection for standard analyses. It uses SAMBLASTER [76] to extract split and discordant reads from BWA-MEM-aligned Binary Sequence Alignment Map (BAM) files. Default options were used, including minimum non-overlap and minimum sample weight set to 20 and 4, respectively. In Manta, the “configManta.py” script was used to process each sample, with default options including minimum variant candidate size (8); minimum candidate spanning count (3); minimum scored variant size (50); minimum diploid variant score (10); minimum diploid variant score pass point (20); minimum somatic score (10); and minimum somatic score pass point (30). The “runWorkflow.py” scripts were run in parallel to extract the SV for each sample.

### Post-processing of SV

SV from LUMPY were genotyped with svtyper version 0.6.1 [77], which uses a Bayesian maximum likelihood algorithm to determine the most likely genotype of each base-pair. Variant call format (VCF) files from the two software packages were converted to browser extensible data (BED) format for downstream analysis using svtools version 0.5.0 [78]. Various levels of SV post-processing parameters were used to come up with the CNV calls from the SV calls. The parameters included: 1) precision of SV calls (whether imprecise SV were included in computation of the CNV calls); 2) point of application of the lower SV length cut-off point (before or after merging Manta and LUMPY SV); 3) stringency of the SV call filters (low, medium, and high stringency); and 4) upper SV length cut-off (3 or 10 Mb). Stringency of SV call filters was in terms of the number of PE and SR required as evidence supporting an SV. Consensus SV were obtained by identifying the intersection of the SV from LUMPY and Manta using BEDTools version 2.26.0 [79] with default settings.

### Derivation of copy number variations

CNV were defined as SV duplications and deletions longer than 50 bp [80]. SV longer that 3 Mb were also filtered out, because putative CNV in the goat genome are usually much shorter than this length. Visualization of the SV was done using R [81] package circlize version 0.4.7 [82].

### Population CNV differentiation

A measure of population differentiation (*V*_*ST*_) as described by Redon et al. [11] was computed based on normalized read count values for each CNV, similar to the method used in PECNV as described by Liu et al. [83], which was in turn based on clustering algorithms described by Cridland et al. [84] and transposable element detection algorithms described by Rogers et al. [85]. Read count values were corrected for size of the consensus CNV, batch effect, variable GC content and genomic mappability as described by Liu et al. [83]. Regional and batch effect correction was done by computing reads per kb per million mapped reads (RPKM) as described by Mortazavi et al. [86], where $RPKM=\frac{10^9\ast RC}{TRC\ast S}$, where RC is the read count of a region, S is the size of the region and TRC is the total number of mapped reads in the library. GC content and mappability correction was done on the RPKM using the formula used by Yoon et al. [29], where adjusted read count is given by $\frac{RPKM\ast m}{m_{GC}}$ where *m*_*GC*_ is the median GC content of all regions with the same read count and *m* is the median GC of all regions. This approach is similar to the read depth approaches used in CNVnator [28] and in CNVcaller [87]. The normalized read count values were treated as proxies of log R ratio (LRR) values normally obtained from array analysis. As defined by Redon et al. [11], *V*_*ST*_ was computed as $\frac{V_T-{V}_S}{V_T}$, where V_T_ is the variance in LRR among all unrelated individuals and V_S_ is the average variance in LRR within each population. CNV *V*_*ST*_ testing was done pairwise (for each combination of two populations) and (separately) across all the 5 populations. CNV with *V*_*ST*_ values above the 99th percentile of all *V*_*ST*_ values for each comparison were treated as being highly differentiated. We searched for these highly differentiated CNV in the Golden Helix Genome Browse® software (version 3.0.0) (https://www.goldenhelix.com/) using the ARS1 caprine genome reference assembly to identify the genes in the CNV.

### Determination of CNV regions

CNV regions (CNVR) were obtained by merging CNV that overlapped by at least 1 bp within populations (population CNVR) and across all the individuals (global CNVR) using the “merge” function in BEDTools version 2.26.0 [79].

### CNVR functional annotation and gene enrichment analysis

A list of genes for the goat genome was downloaded from the NCBI website (https://www.ncbi.nlm.nih.gov/gene). The Database for Annotation, Visualization, and Integrated Discovery (DAVID) Bioinformatics Resources (version 6.8) [88–90] was used to identify if genes in the CNVR have significant biological, cellular or molecular function. Functional analysis was done using default parameters, with significance of enriched terms determined at *P* ≤ 0.05. Further information about various genes was obtained from the GeneCards (www.genecards.org) database.

## Supplementary Information

**Additional file 1 Supplementary Table 1** Genes in CNV with V_ST_ values above the 99th percentile for each comparison. **Supplementary Table 2** List of CNVR by population. **Supplementary Table 3** Summary of CNVR by population. **Supplementary Table 4** List of global CNVR with frequency above 1%. **Supplementary Table 5** Functional annotation and clustering analysis for global CNVR. **Supplementary Table 6** Functional analysis for CNVR private to breeds and to populations. **Supplementary Table 7** Sample processing details. **Supplementary Table 8** Quality control details for the samples used in the study.

**Additional file 2 Supplementary Figures 1–10** CNV differentiation between the following populations; respectively: Boer and East African; Boer and Madagascar; Boer and Southern African; Boer and West African; East African and Madagascar; East African and Southern African; East African and West African; Madagascar and Southern African; Madagascar and West African and Southern African and Western African. **Supplementary Figures 11–39** CNVR for the following goat breeds, respectively: Abergelle (Ethiopia), Androy (Madagascar), Balaka-Ulongwe (Malawi), Boer (Tanzania and Zimbabwe), Dedza (Malawi), Diana (Madagascar), Galla (Kenya), Gogo (Tanzania), Guera (Mali), Gumez (Ethiopia), Keffa (Ethiopia), Landin (Mozambique), Maasai (Tanzania), Malya (Tanzania), Manica (Mozambique), Mashona (Zimbabwe), Matebele (Zimbabwe), Menabe (Madagascar), Naine (Mali), Norwegian (Tanzania), Nsanje (Malawi), Pare White (Tanzania), Saanen (Tanzania), Small East African (Kenya and Mozambique), Sofia (Madagascar), Soudanaise (Mali), SudOuest (Madagascar), Thyolo (Malawi) and Woyito Guji (Ethiopia). **Supplementary Figures 40–64** Global CNVR with variable SV calling parameters.

## Data Availability

The dataset supporting the conclusions of this article is available at the National Center for Biotechnology Information Sequence Read Archive as detailed in Additional file 1 (Supplementary Table 7).

## Abbreviations

ABR: Abergelle
AND: Androy
BAM: Binary Sequence Alignment Map
BAW: Balaka-Ulongwe
BED: Browser Extensible Data
BOE: Boer
BWA: Burrow-Wheeler Alignment
CGH: Comparative genomic hybridization
CNV: Copy number variation
CNVR: Copy number variation region
DAVID: Database for Annotation, Visualization, and Integrated Discovery
DIA: Diana
DZD: Dedza
EAF: East African
EWT: Event-wise testing
GAL: Galla
GO: Gene Ontology
GOG: Gogo
GSEA: Gene set enrichment analysis
GUE: Guerra
GUM: Gumez
KEF: Keffa
KEGG: Kyoto Encyclopedia of Genes and Genomes
LGW: Lilongwe
LND: Landin
MAA: Maasai
MAD: Madagascar
MAN: Manica
MAU: Maure
MEM: Maximal exact matches
MEN: Menabe
MLY: Malya
MSH: Mashona
MTB: Matebele
NAI: Naine
NRW: Norwegian
NSJ: Nsanje
PE: Paired-end mapping
PEU: Peulh
PR: Paired-end reads
PRW: Pare White
RD: Read depth
RPKM: Reads per kb per million mapped reads
SAA: Saanen
SAF: Southern African
SDN: Soudanaise
SEA: Small East African
SNJ: Sonjo
SNP: Single nucleotide polymorphisms
SOF: Sofia
SOU: SudOuest
SR: Split reads
SV: Structural variation
TAR: Targui
THY: Thyolo
VCF: Variant call format
WAF: West African
WGS: Whole genome sequencing
WYG: Woyito Guji
