# Supplementary material for: Detection of copy number variants in African goats using whole genome sequence data
Source: BMC Genomics. 2021 May 29;22:398. doi: 10.1186/s12864-021-07703-1 (PMC8164248; doi:10.1186/s12864-021-07703-1)
Supplement: Supplementary file 2 — Additional file 2 Supplementary Figures 1–10 CNV differentiation between the following populations; respectively: Boer and East African; Boer and Madagascar; Boer and Southern African; Boer and West African; East African and Madagascar; East African and Southern African; East African and West African; Madagascar and Southern African; Madagascar and West African and Southern African and Western African. Supplementary Figures 11–39 CNVR for the following goat breeds, respectively: Abergelle (Ethiopia), Androy (Madagascar), Balaka-Ulongwe (Malawi), Boer (Tanzania and Zimbabwe), Dedza (Malawi), Diana (Madagascar), Galla (Kenya), Gogo (Tanzania), Guera (Mali), Gumez (Ethiopia), Keffa (Ethiopia), Landin (Mozambique), Maasai (Tanzania), Malya (Tanzania), Manica (Mozambique), Mashona (Zimbabwe), Matebele (Zimbabwe), Menabe (Madagascar), Naine (Mali), Norwegian (Tanzania), Nsanje (Malawi), Pare White (Tanzania), Saanen (Tanzania), Small East African (Kenya and Mozambique), Sofia (Madagascar), Soudanaise (Mali), SudOuest (Madagascar), Thyolo (Malawi) and Woyito Guji (Ethiopia). Supplementary Figures 40–64 Global CNVR with variable SV calling parameters. [file 12864_2021_7703_MOESM2_ESM.docx]

**Detection of copy number variants in African goats using whole genome sequence data**

**Wilson Nandolo^1,2^, Gábor Mészáros^1^, Maria Wurzinger^1^, Liveness Jessica Banda^2^, Timothy N. Gondwe^2^, Henry Aaron Mulindwa^3^, Helen N. Nakimbugwe^4^, Emily L. Clark^5^, M. Jennifer Woodward-Greene^6^, Mei Liu^6^, George E. Liu^6^, Curtis P. Van Tassell^6^, Benjamin D. Rosen^6^, Johann Sölkner^1^**

1 University of Natural Resources and Life Sciences, Vienna, Austria

2 Lilongwe University of Agriculture and Natural Resources, Lilongwe, Malawi

3 National Livestock Resources Research Institute, Tororo, Uganda

4 Kyambogo University, Kyambogo, Uganda

5 The Roslin Institute, University of Edinburgh, Edinburgh, Scotland, United Kingdom

6 Animal Genomics and Improvement Laboratory, USDA-ARS, Beltsville, MD, USA

Corresponding author: Benjamin D. Rosen (Ben.Rosen@usda.gov)

# List of figures

Supplementary Figure 1: CNV differentiation between Boer and East African goat populations. 3

Supplementary Figure 2: CNV differentiation between Boer and Madagascar goat populations. 3

Supplementary Figure 3: CNV differentiation between Boer and Southern African goat populations. 4

Supplementary Figure 4: CNV differentiation between Boer and West African goat populations. 5

Supplementary Figure 5: CNV differentiation between East African and Madagascar goat populations. 5

Supplementary Figure 6: CNV differentiation between East African and Southern African goat populations. 6

Supplementary Figure 7: CNV differentiation between East African and West African goat populations. 6

Supplementary Figure 8: CNV differentiation between Madagascar and Southern African goat populations. 7

Supplementary Figure 9: CNV differentiation between Madagascar and West African goat populations. 7

Supplementary Figure 10: CNV differentiation between Southern African and Western African goat populations. 8

Supplementary Figure 11: Proportions of CNV copy loss and gain for each set of filter parameter and CNV cut-off point. 8

Supplementary Figure 11: CNVR for the Abergelle breed 9

Supplementary Figure 12: CNVR for the Androy breed 9

Supplementary Figure 13: CNVR for the Balaka-Ulongwe breed 10

Supplementary Figure 14: CNVR for the Boer breed 10

Supplementary Figure 15: CNVR for the Dedza breed 11

Supplementary Figure 16: CNVR for the Diana breed 11

Supplementary Figure 17: CNVR for the Galla breed 12

Supplementary Figure 18: CNVR for the Gogo breed 12

Supplementary Figure 19: CNVR for the Guera breed 13

Supplementary Figure 20: CNVR for the Gumez breed 13

Supplementary Figure 21: CNVR for the Keffa breed 14

Supplementary Figure 22: CNVR for the Landin breed 14

Supplementary Figure 23: CNVR for the Maasai breed 15

Supplementary Figure 24: CNVR for the Malya breed 15

Supplementary Figure 25: CNVR for the Manica breed 16

Supplementary Figure 26: CNVR for the Mashona breed 16

Supplementary Figure 27: CNVR for the Matebele breed 17

Supplementary Figure 28: CNVR for the Menabe breed 17

Supplementary Figure 29: CNVR for the Naine breed 18

Supplementary Figure 30: CNVR for the Norwegian breed 18

Supplementary Figure 31: CNVR for the Nsanje breed 19

Supplementary Figure 32: CNVR for the Pare White breed 19

Supplementary Figure 33: CNVR for the Saanen breed 20

Supplementary Figure 34: CNVR for the Small East African breed 20

Supplementary Figure 35: CNVR for the Sofia breed 21

Supplementary Figure 36: CNVR for the Soudanaise breed 21

Supplementary Figure 37: CNVR for the SudOuest breed 22

Supplementary Figure 38: CNVR for the Thyolo breed 23

Supplementary Figure 39: CNVR for the Woyito Guji breed 23

Supplementary Figure 40: Global CNVR with variable SV calling parameters 24

Supplementary Figure 41: Global CNVR with variable SV calling parameters 25

Supplementary Figure 42: Global CNVR with variable SV calling parameters 26

Supplementary Figure 43: Global CNVR with variable SV calling parameters 27

Supplementary Figure 44: Global CNVR with variable SV calling parameters 28

Supplementary Figure 45: Global CNVR with variable SV calling parameters 29

Supplementary Figure 46: Global CNVR with variable SV calling parameters 30

Supplementary Figure 47: Global CNVR with variable SV calling parameters 31

Supplementary Figure 48: Global CNVR with variable SV calling parameters 32

Supplementary Figure 49: Global CNVR with variable SV calling parameters 33

Supplementary Figure 50: Global CNVR with variable SV calling parameters 34

Supplementary Figure 51: Global CNVR with variable SV calling parameters 35

Supplementary Figure 52: Global CNVR with variable SV calling parameters 36

Supplementary Figure 53: Global CNVR with variable SV calling parameters 37

Supplementary Figure 54: Global CNVR with variable SV calling parameters 38

Supplementary Figure 55: Global CNVR with variable SV calling parameters 39

Supplementary Figure 56: Global CNVR with variable SV calling parameters 40

Supplementary Figure 57: Global CNVR with variable SV calling parameters 41

Supplementary Figure 58: Global CNVR with variable SV calling parameters 42

Supplementary Figure 59: Global CNVR with variable SV calling parameters 43

Supplementary Figure 60: Global CNVR with variable SV calling parameters 44

Supplementary Figure 61: Global CNVR with variable SV calling parameters 45

Supplementary Figure 62: Global CNVR with variable SV calling parameters 46

Supplementary Figure 64: Global CNVR with variable SV calling parameters 47


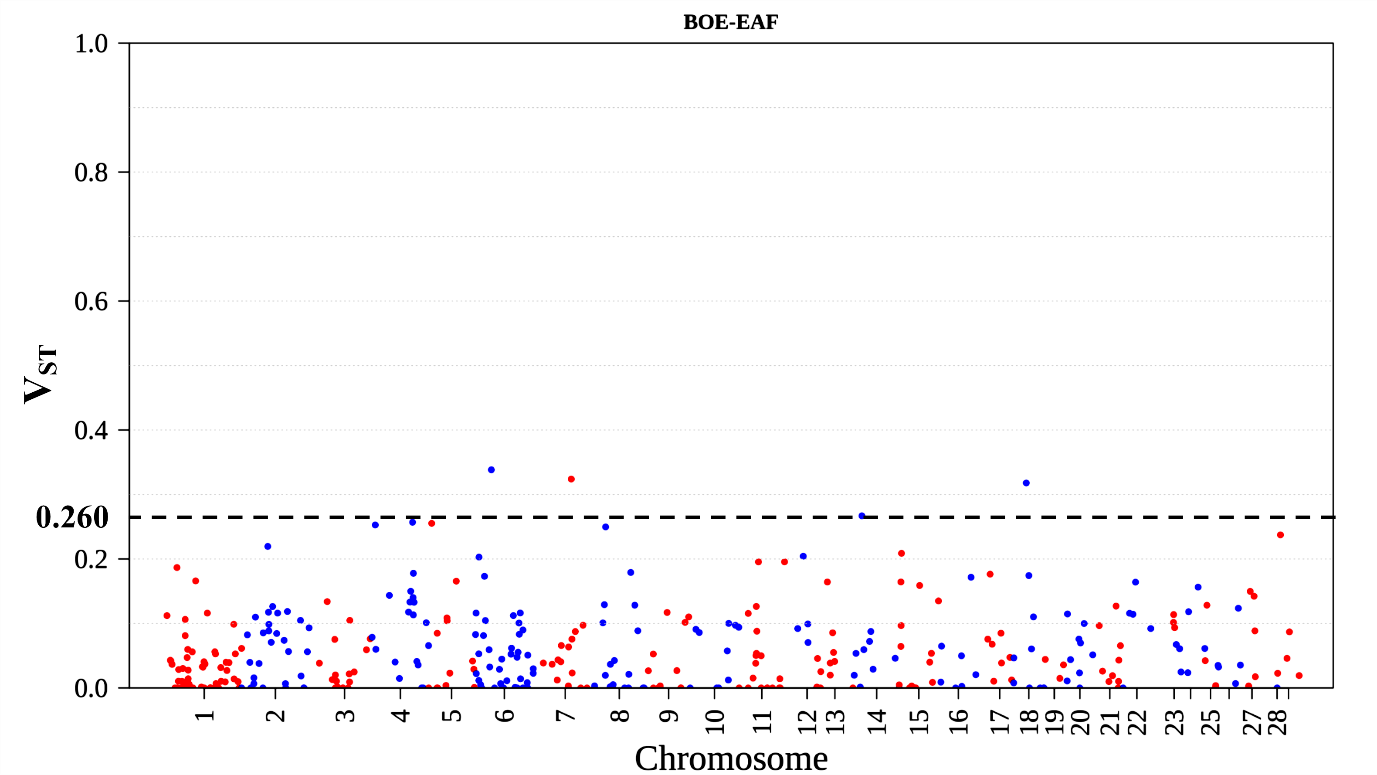


Supplementary Figure 1: CNV differentiation between Boer and East African goat populations.

The dotted line represents the *V_ST_* threshold value for this test.


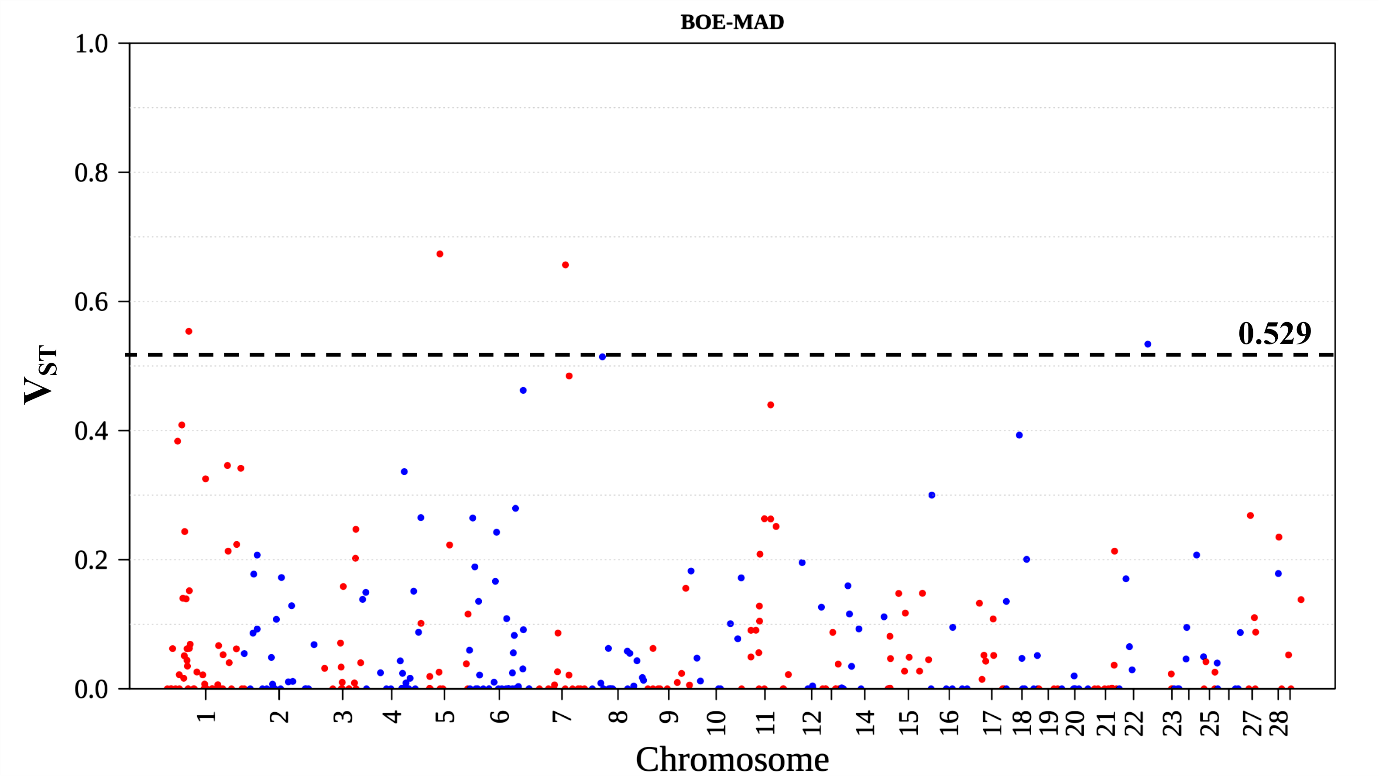


Supplementary Figure 2: CNV differentiation between Boer and Madagascar goat populations.

The dotted line represents the *V_ST_* threshold value for this test.


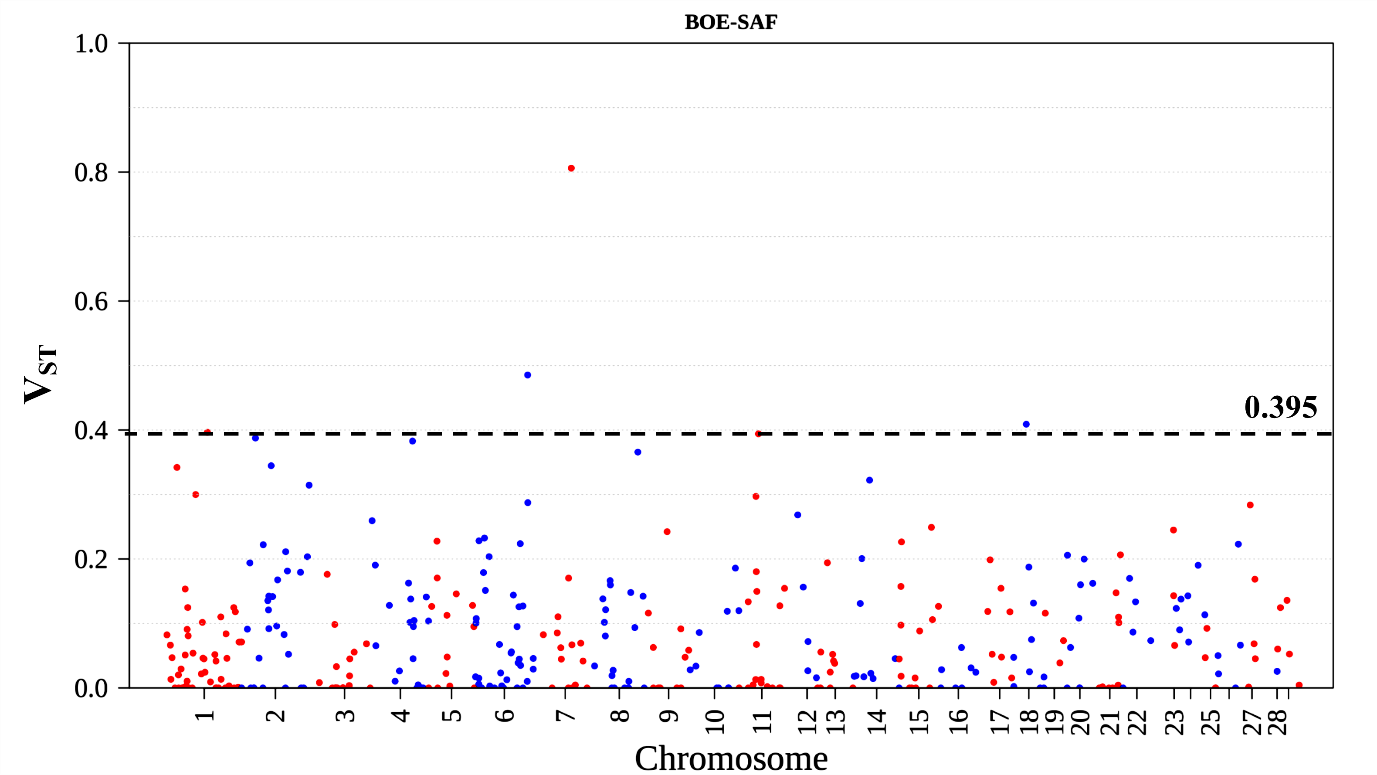


Supplementary Figure 3: CNV differentiation between Boer and Southern African goat populations.

The dotted line represents the *V_ST_* threshold value for this test.


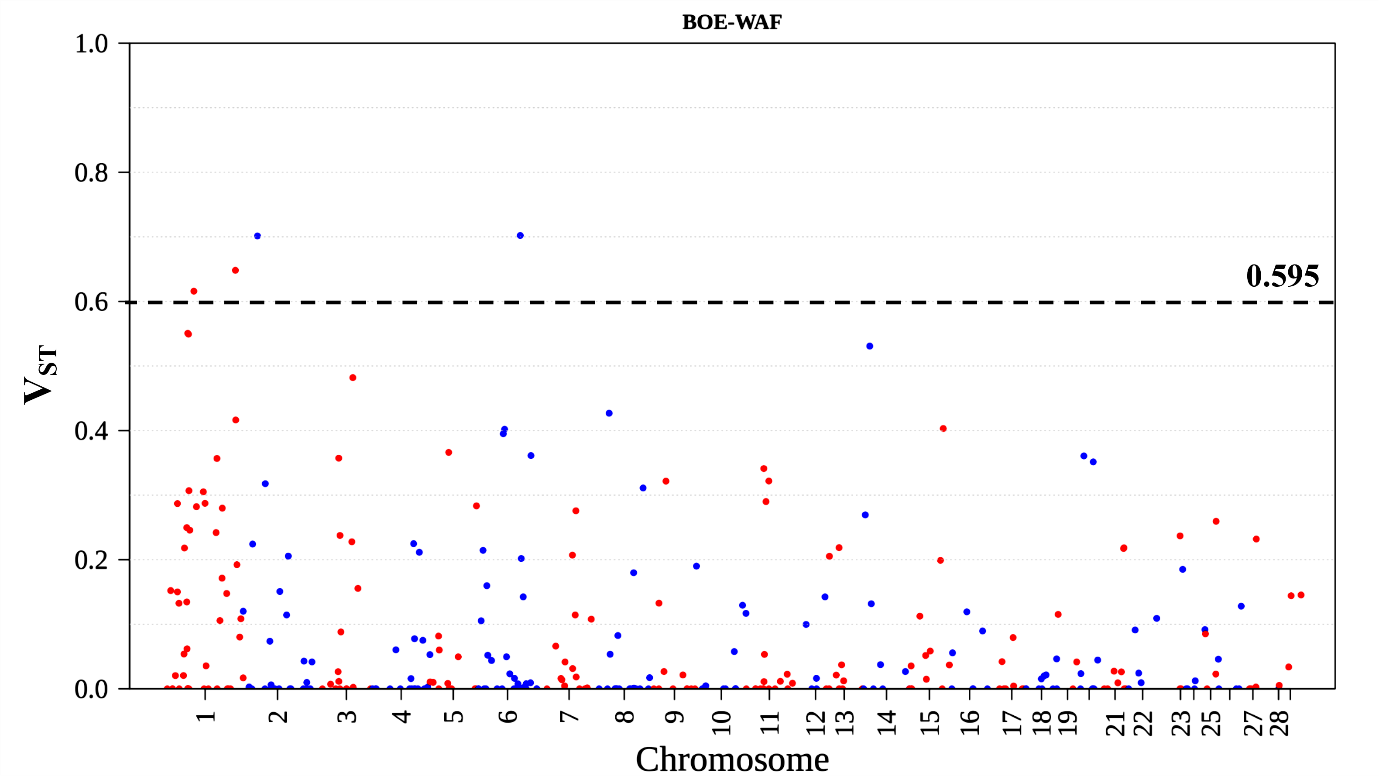


Supplementary Figure 4: CNV differentiation between Boer and West African goat populations.

The dotted line represents the *V_ST_* threshold value for this test.


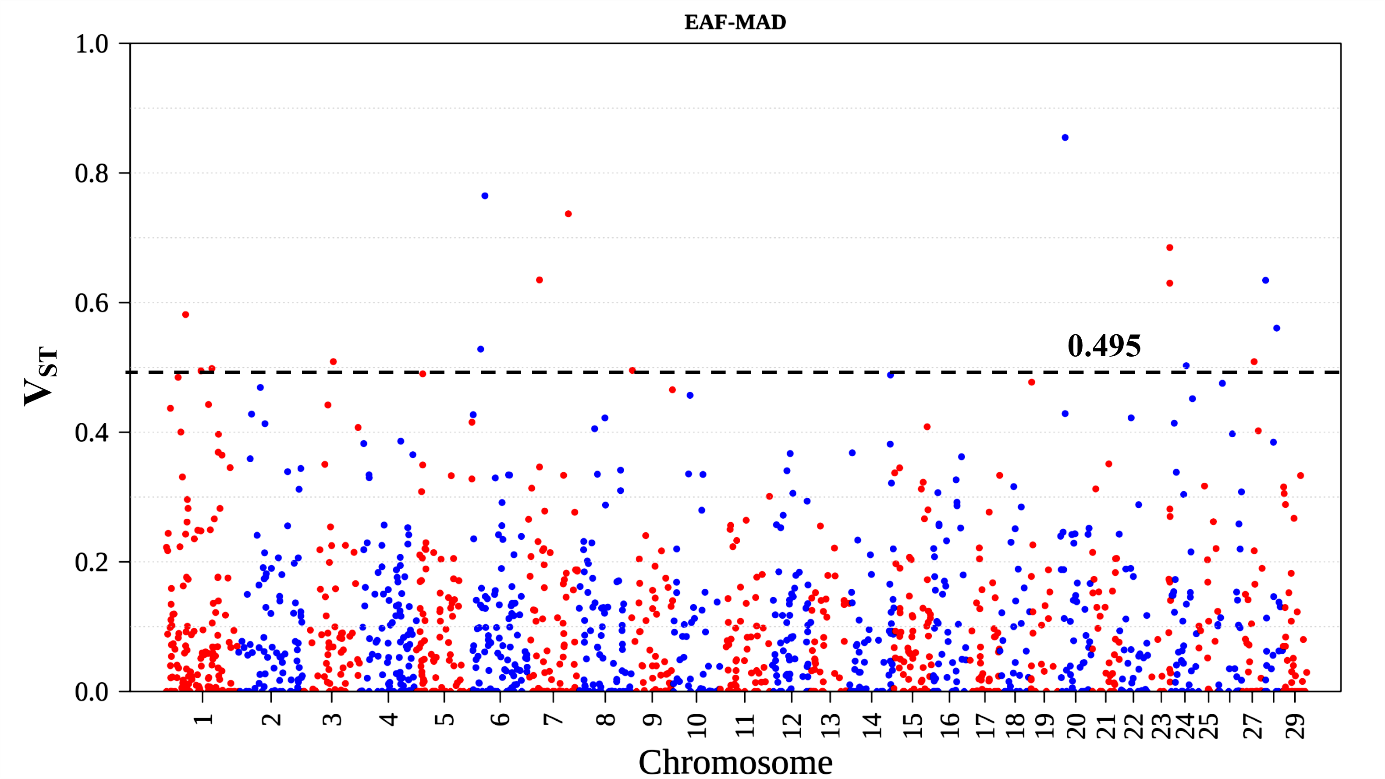


Supplementary Figure 5: CNV differentiation between East African and Madagascar goat populations.

The dotted line represents the *V_ST_* threshold value for this test.


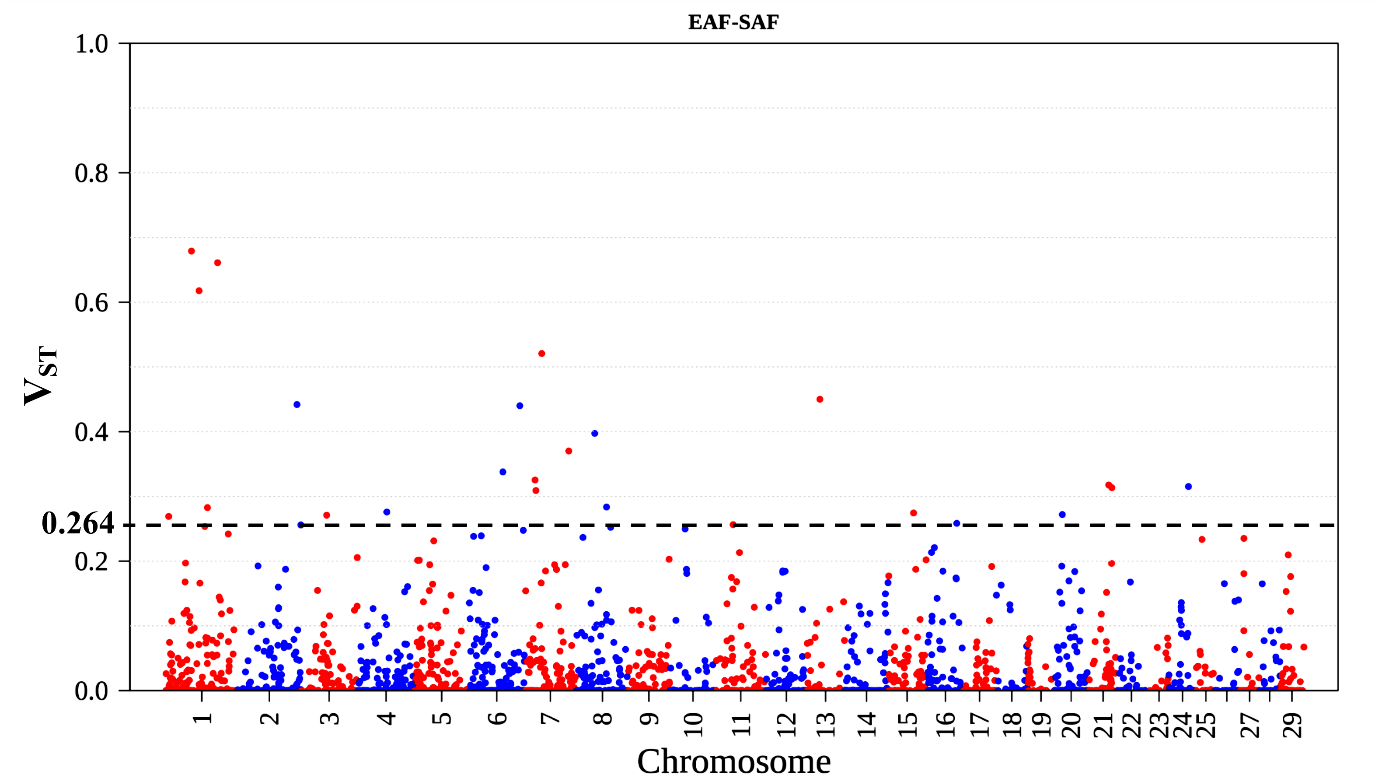


Supplementary Figure 6: CNV differentiation between East African and Southern African goat populations.

The dotted line represents the *V_ST_* threshold value for this test.


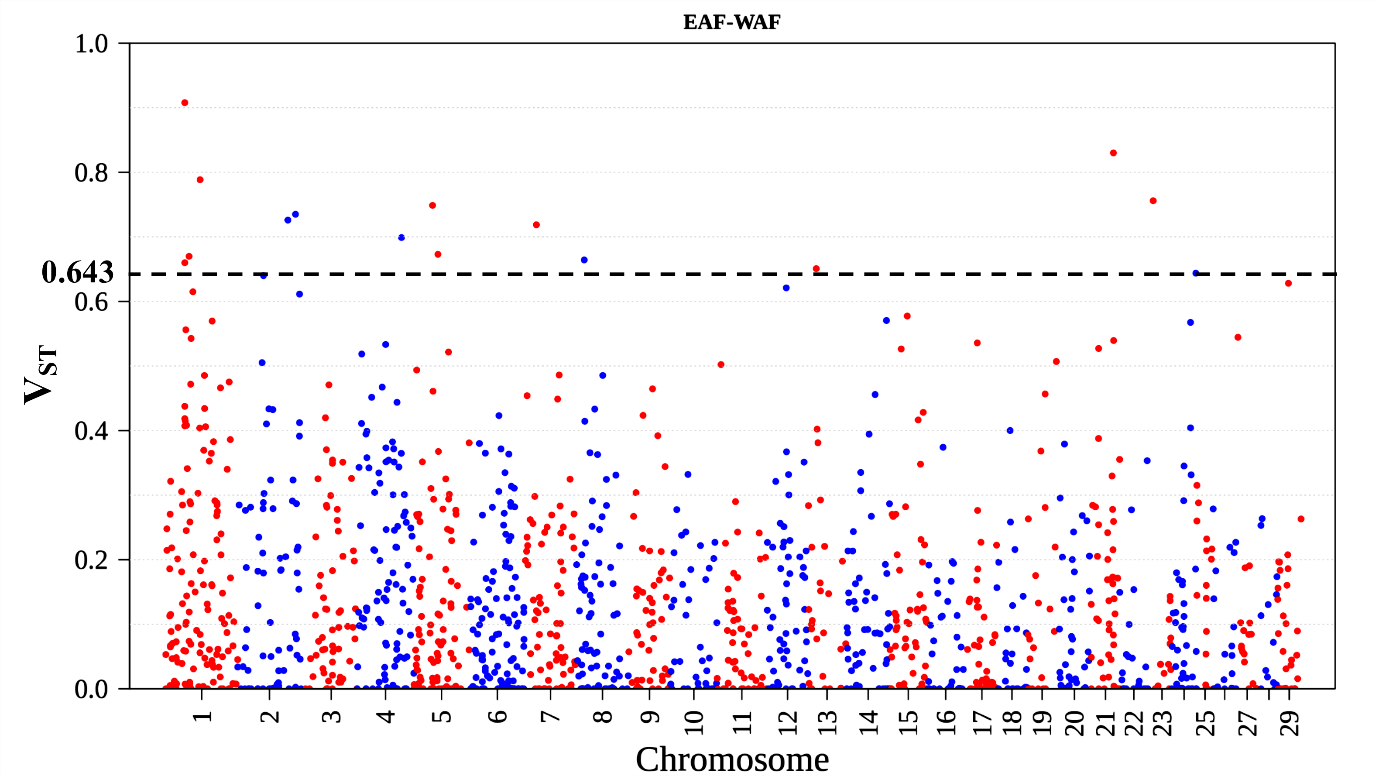


Supplementary Figure 7: CNV differentiation between East African and West African goat populations.

The dotted line represents the *V_ST_* threshold value for this test.


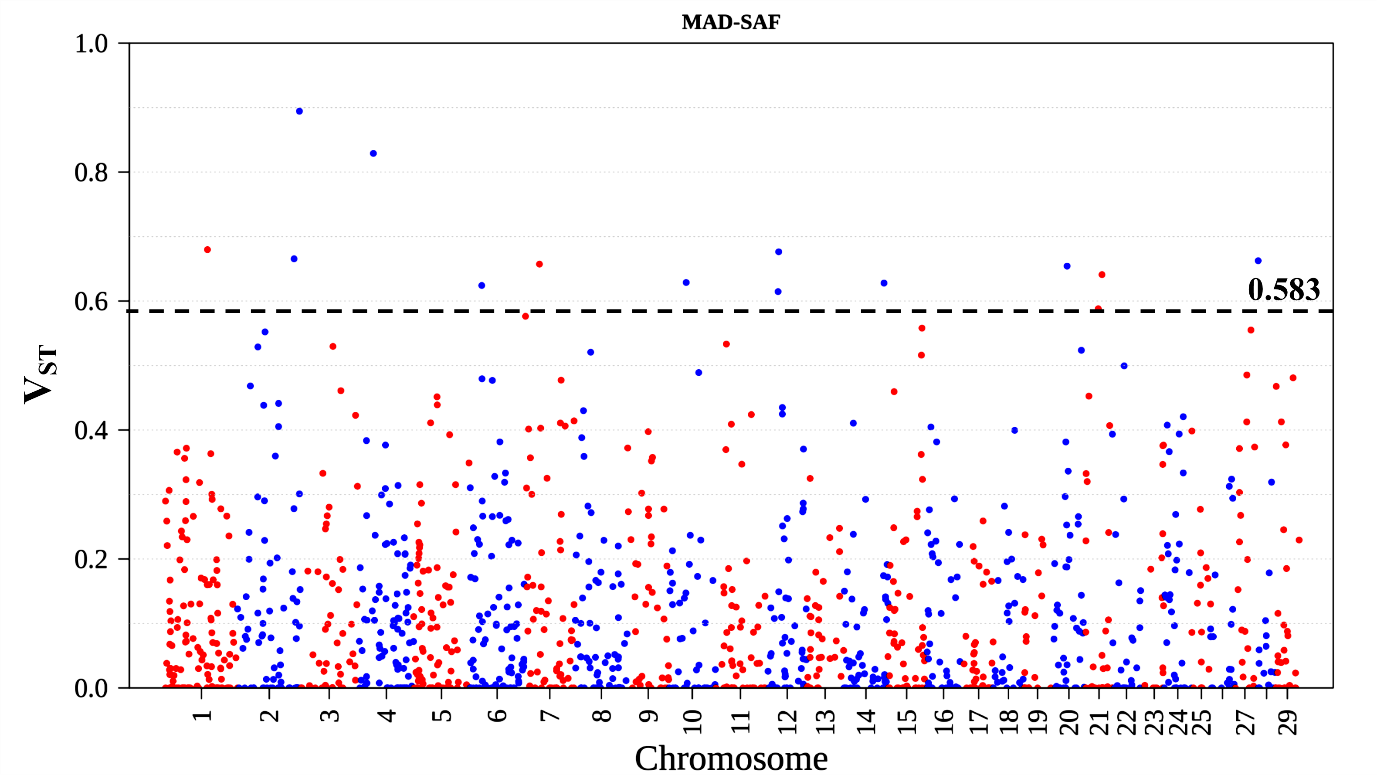


Supplementary Figure 8: CNV differentiation between Madagascar and Southern African goat populations.

The dotted line represents the *V_ST_* threshold value for this test.


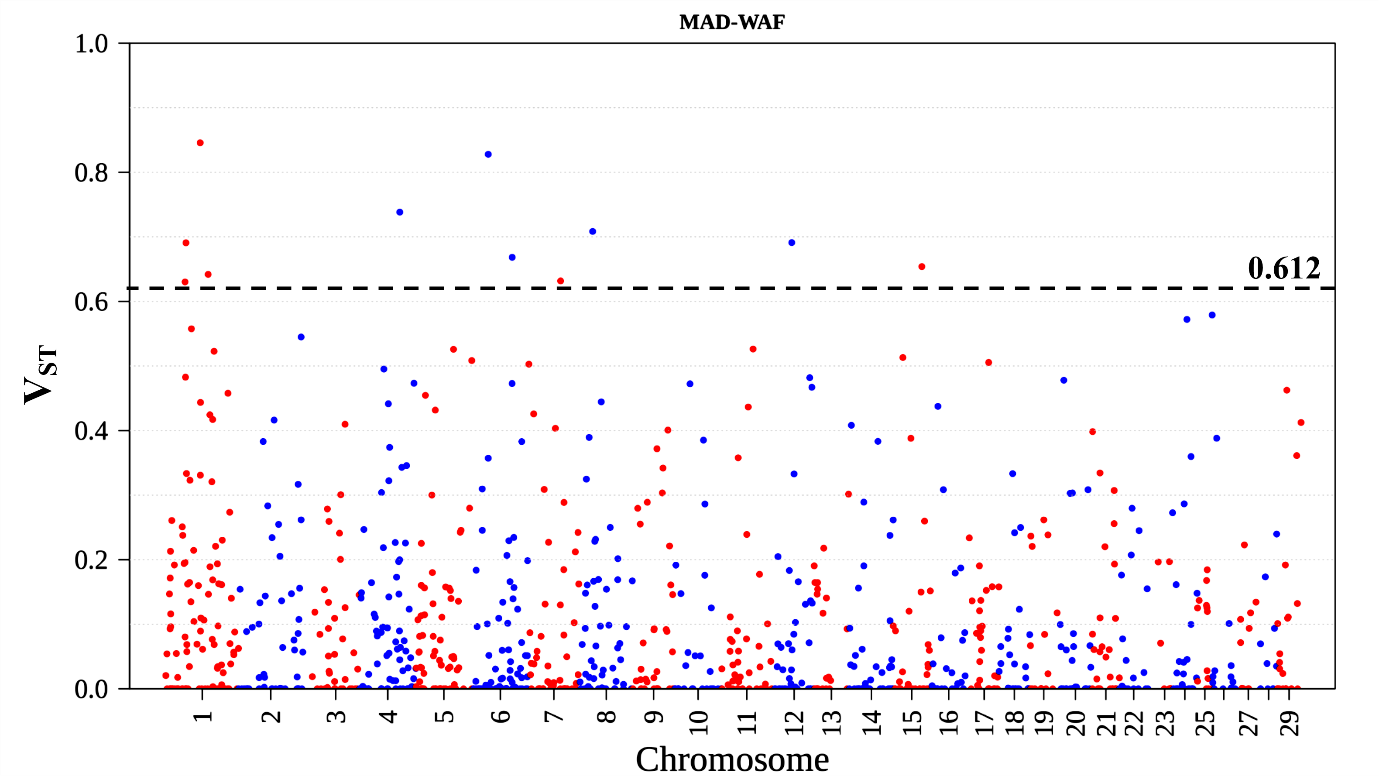


Supplementary Figure 9: CNV differentiation between Madagascar and West African goat populations.

The dotted line represents the *V_ST_* threshold value for this test.


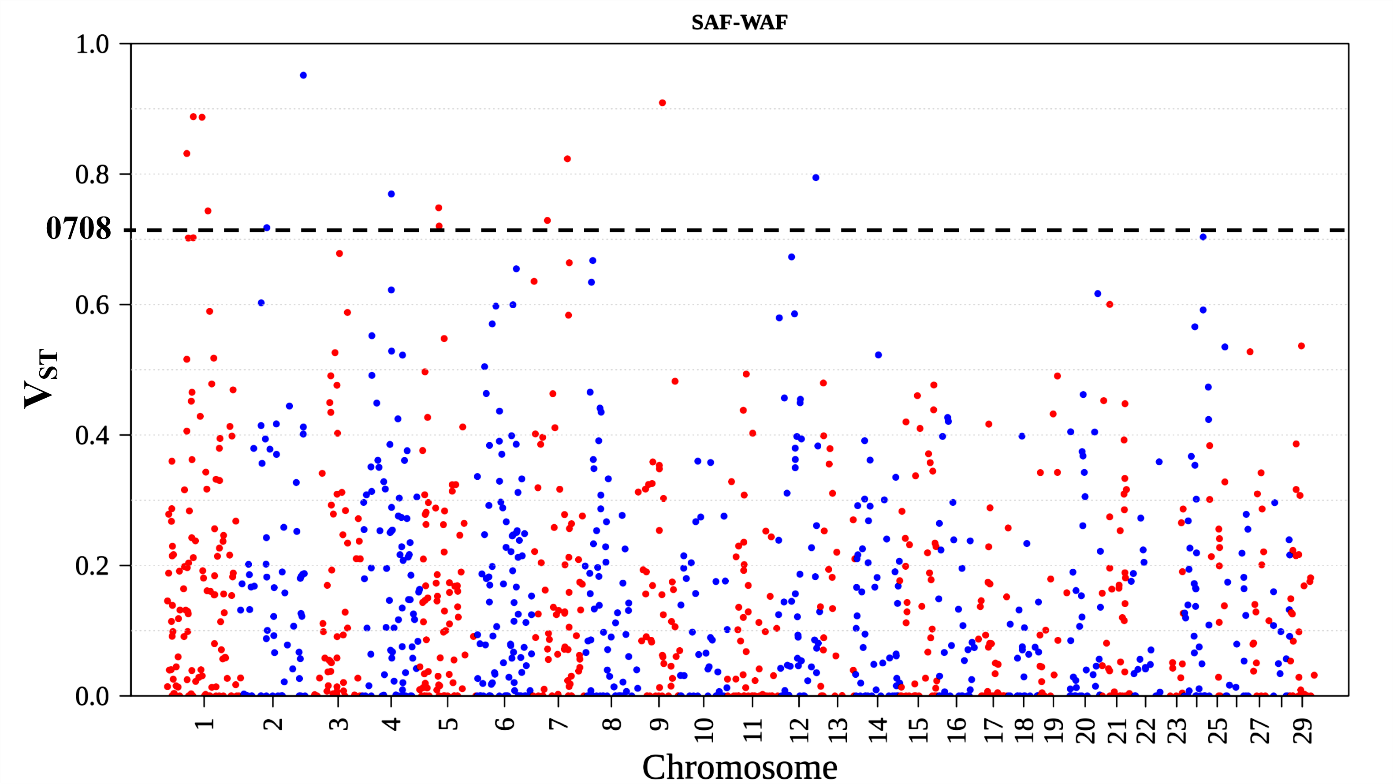


Supplementary Figure 10: CNV differentiation between Southern African and Western African goat populations.

The dotted line represents the *V_ST_* threshold value for this test.


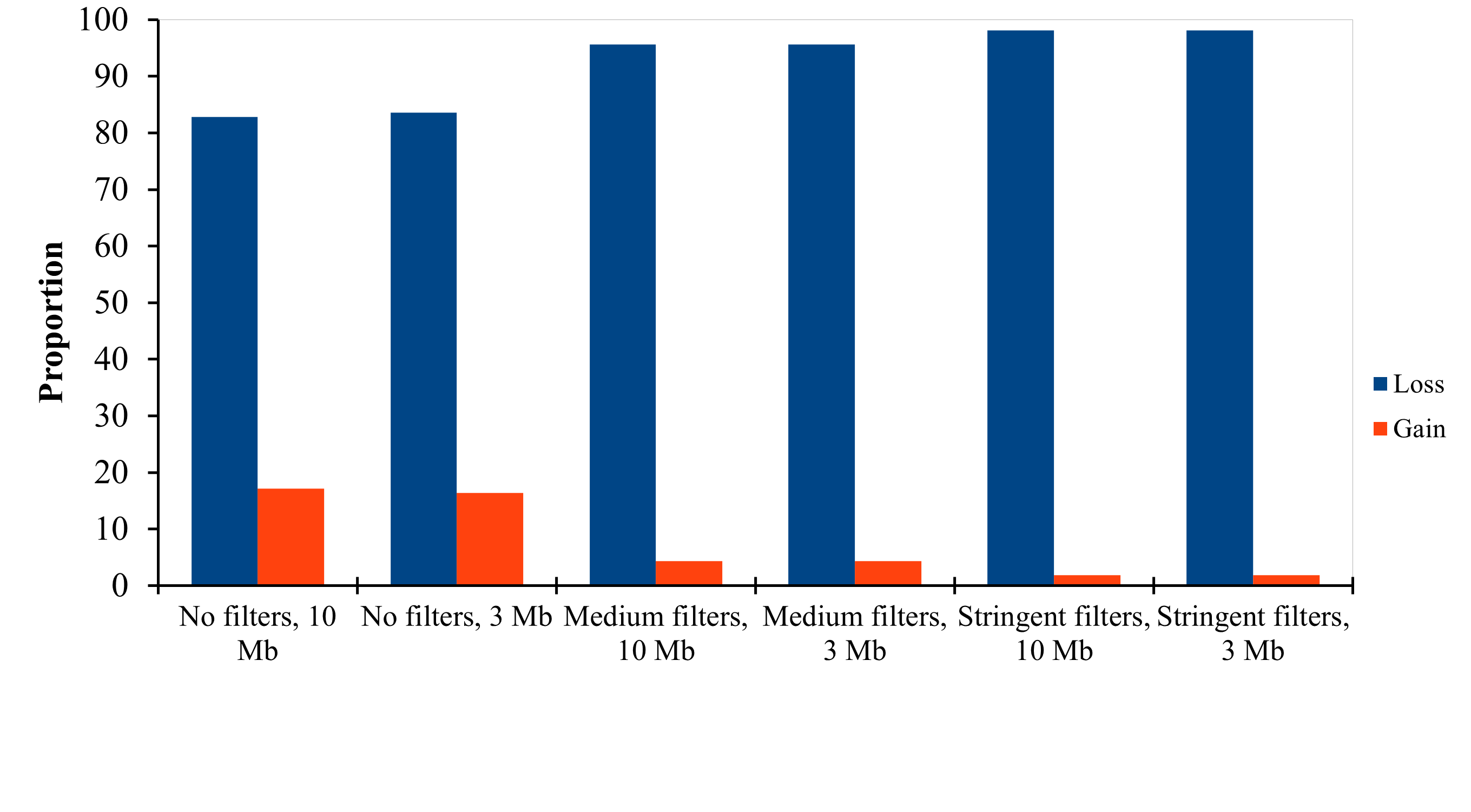


Supplementary Figure 11: Proportions of CNV copy loss and gain for each set of filter parameter and CNV cut-off point.

Medium filters: PE = SR = 3

Stringent filters: PE= SR = 5


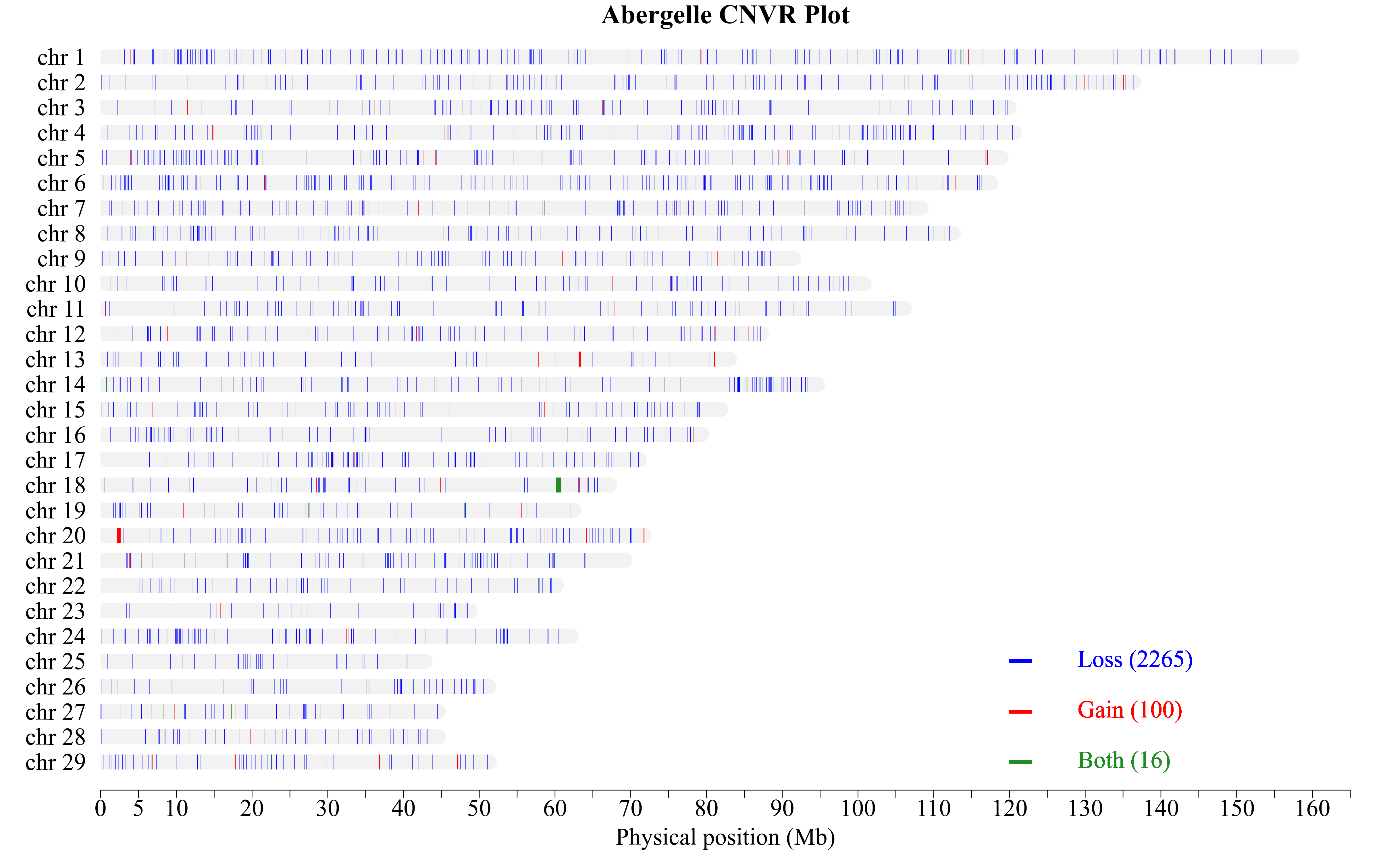


Supplementary Figure 12: CNVR for the Abergelle breed


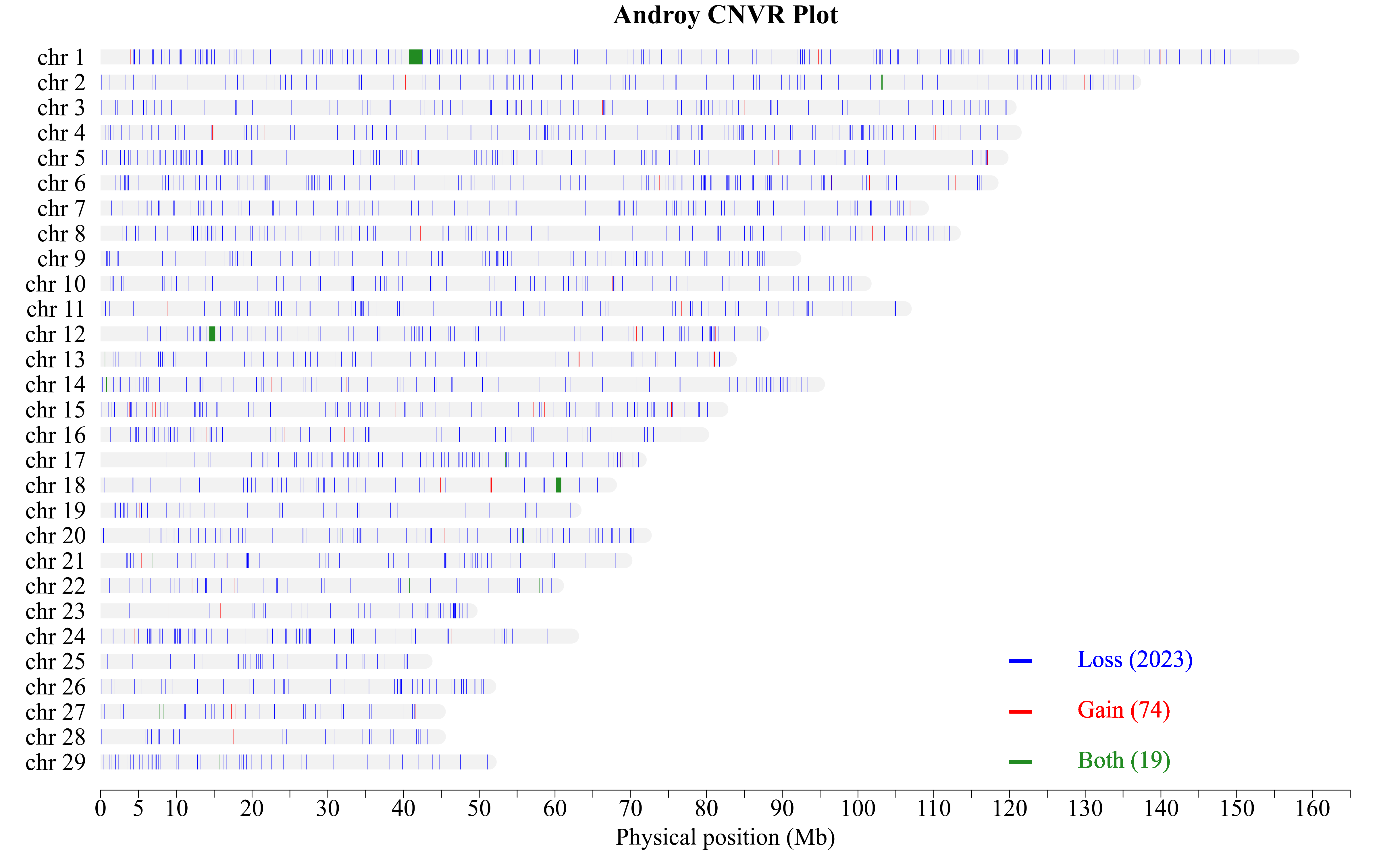


Supplementary Figure 13: CNVR for the Androy breed


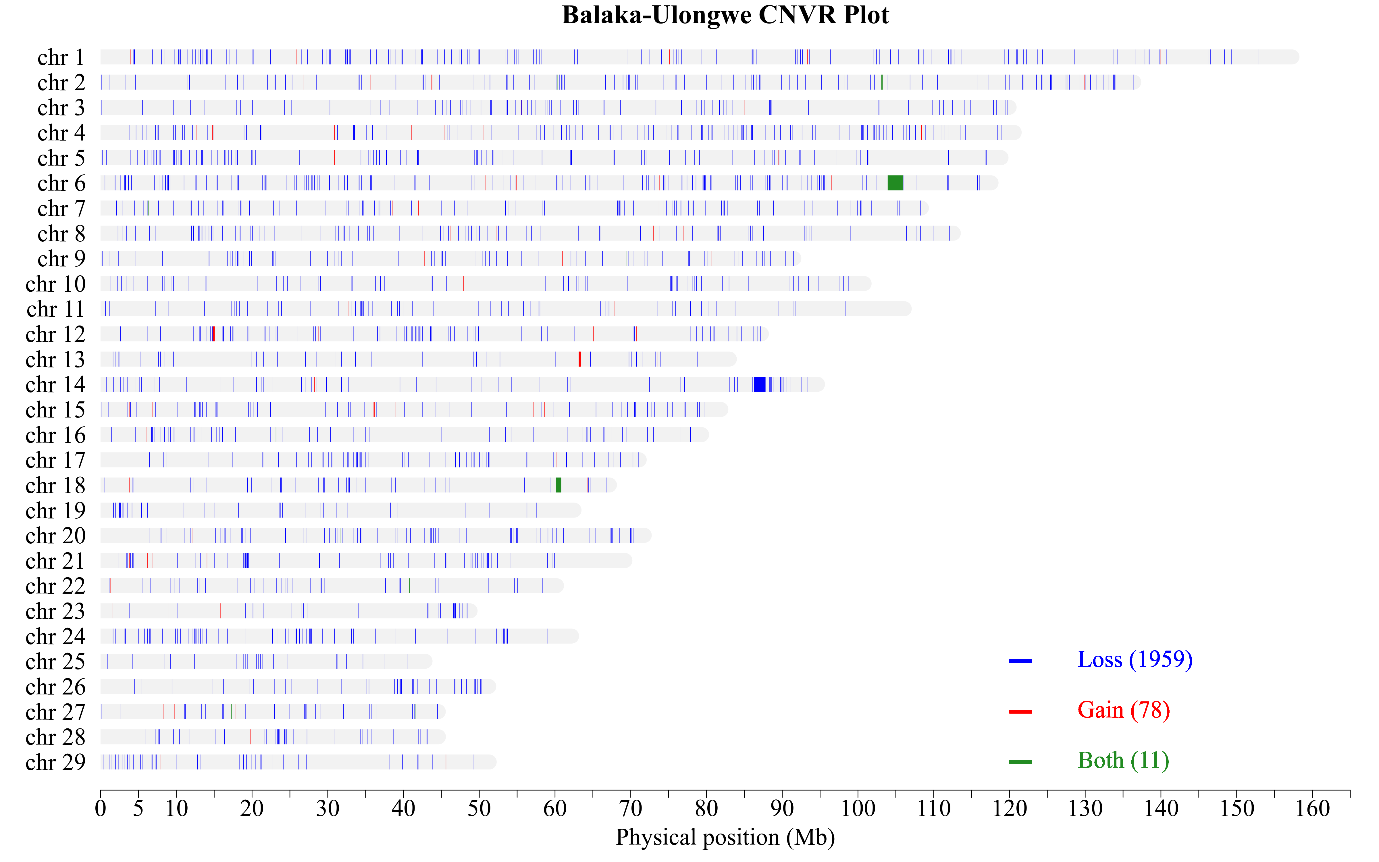


Supplementary Figure 14: CNVR for the Balaka-Ulongwe breed


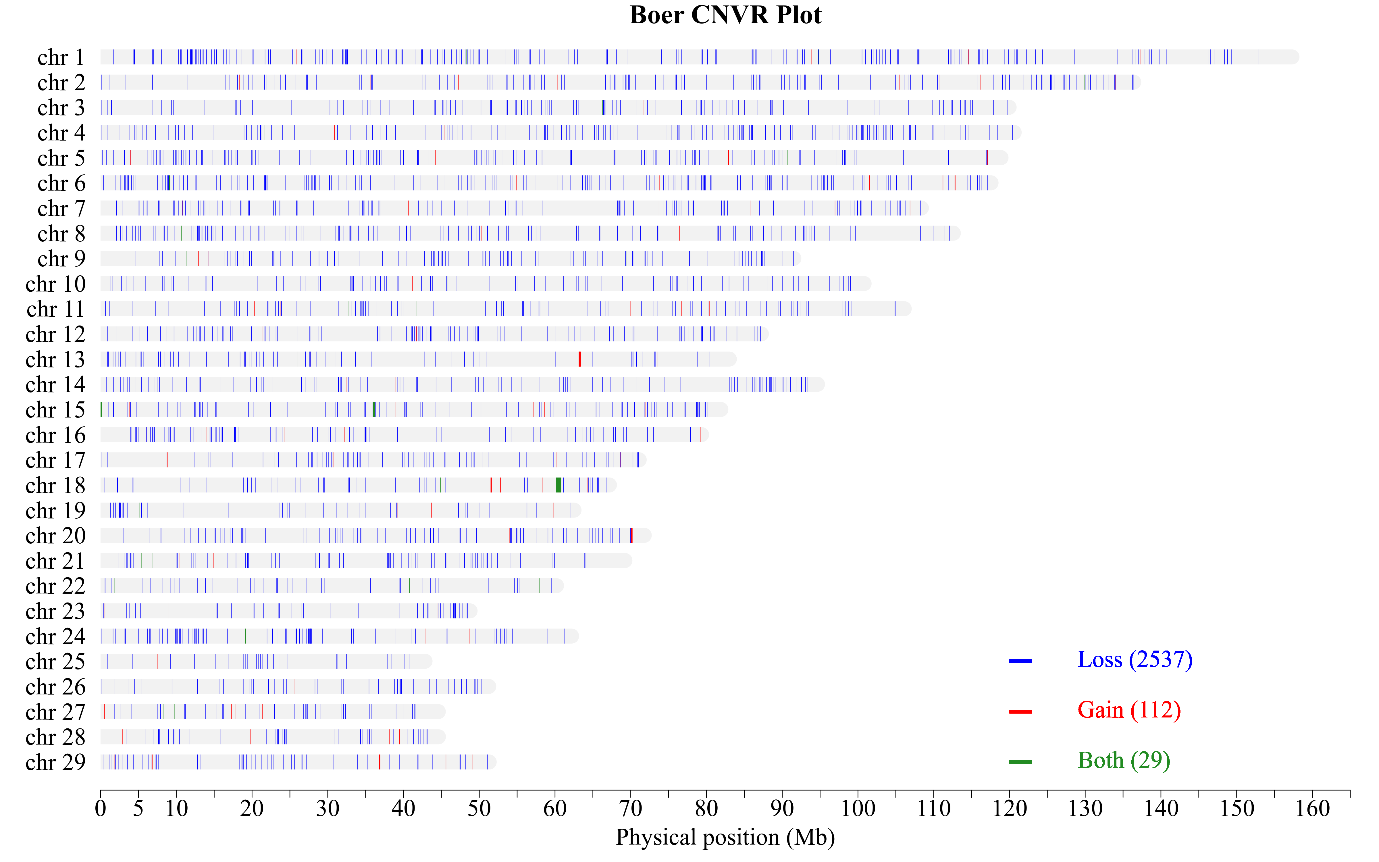


Supplementary Figure 15: CNVR for the Boer breed


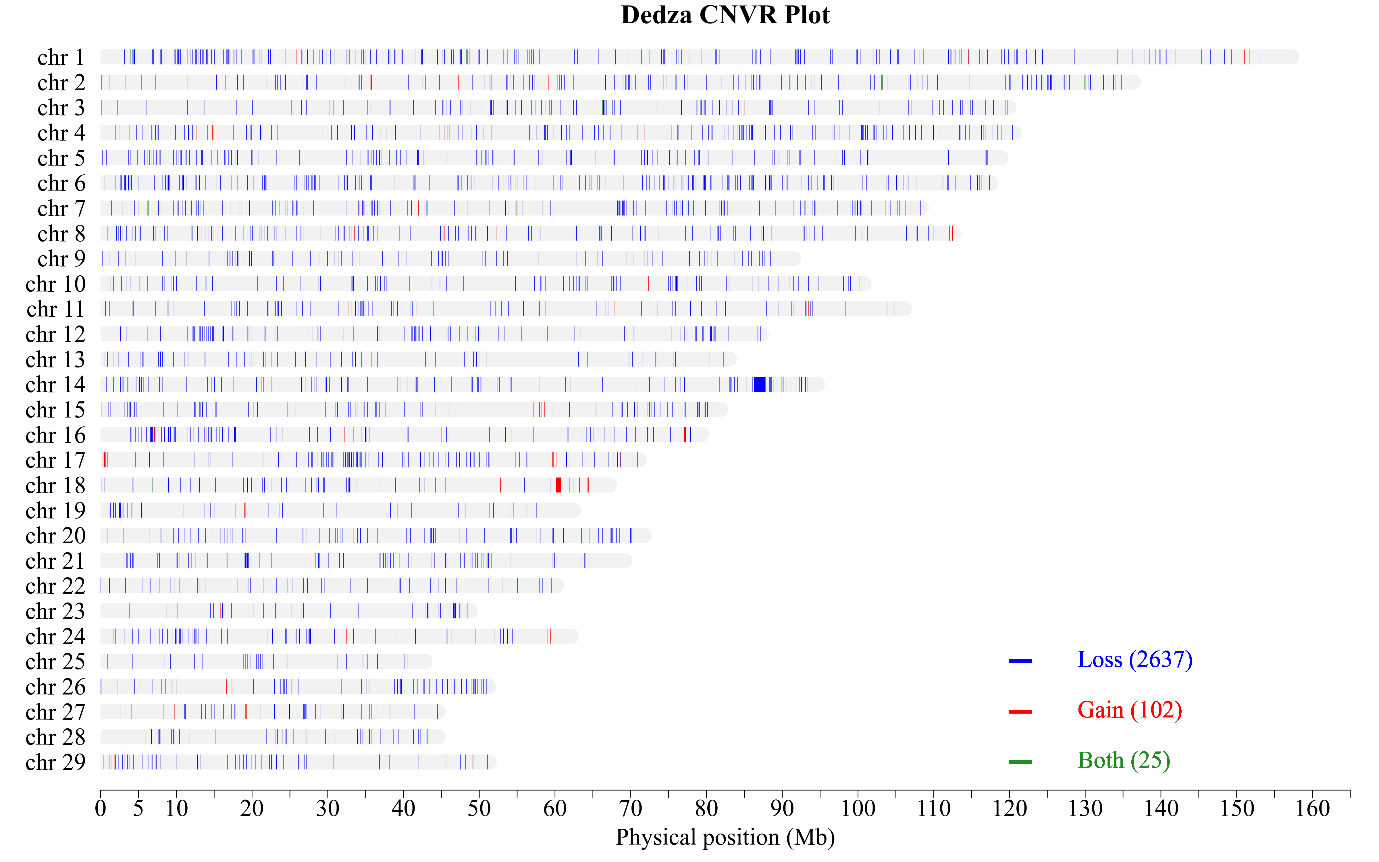


Supplementary Figure 16: CNVR for the Dedza breed


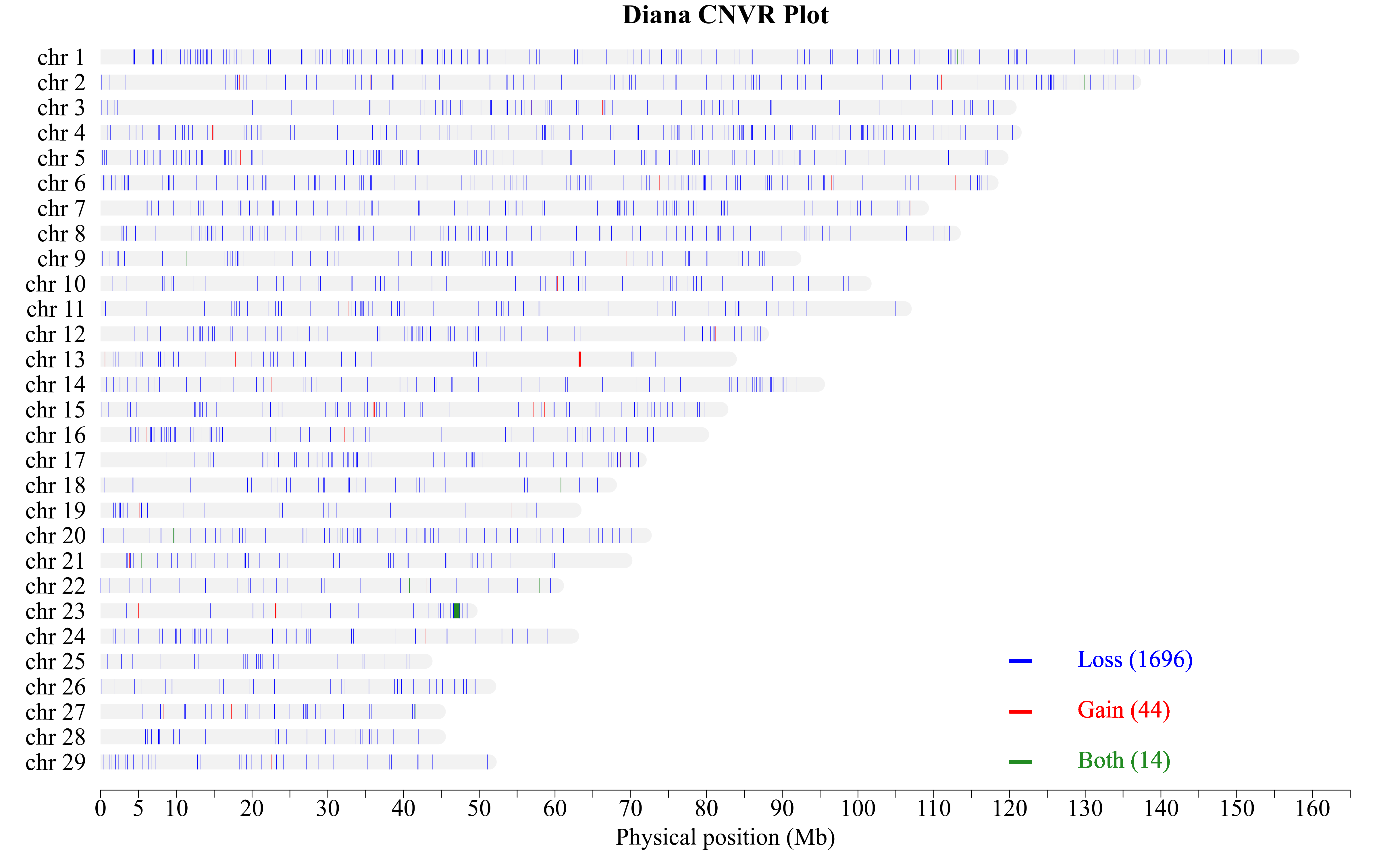


Supplementary Figure 17: CNVR for the Diana breed


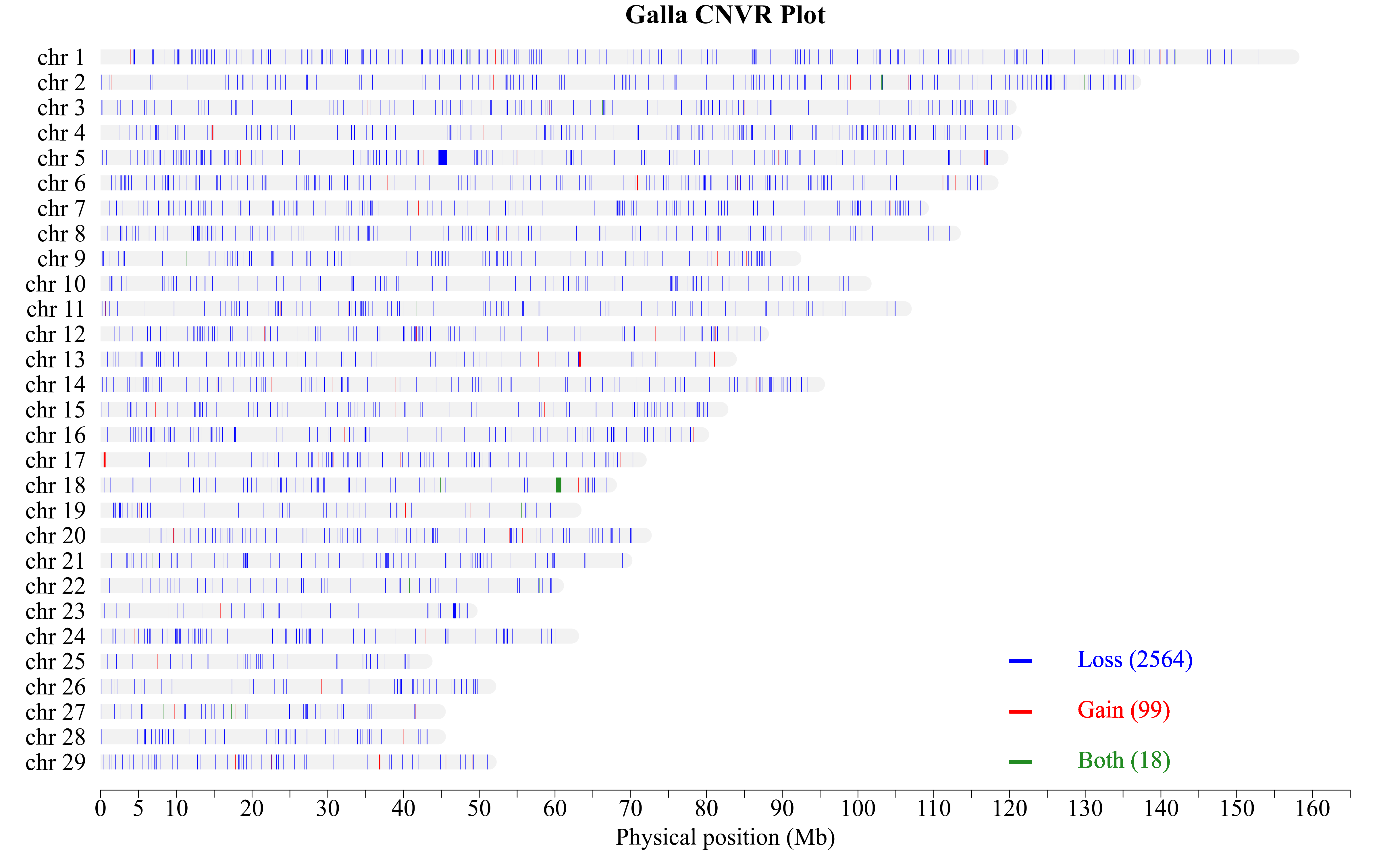


Supplementary Figure 18: CNVR for the Galla breed


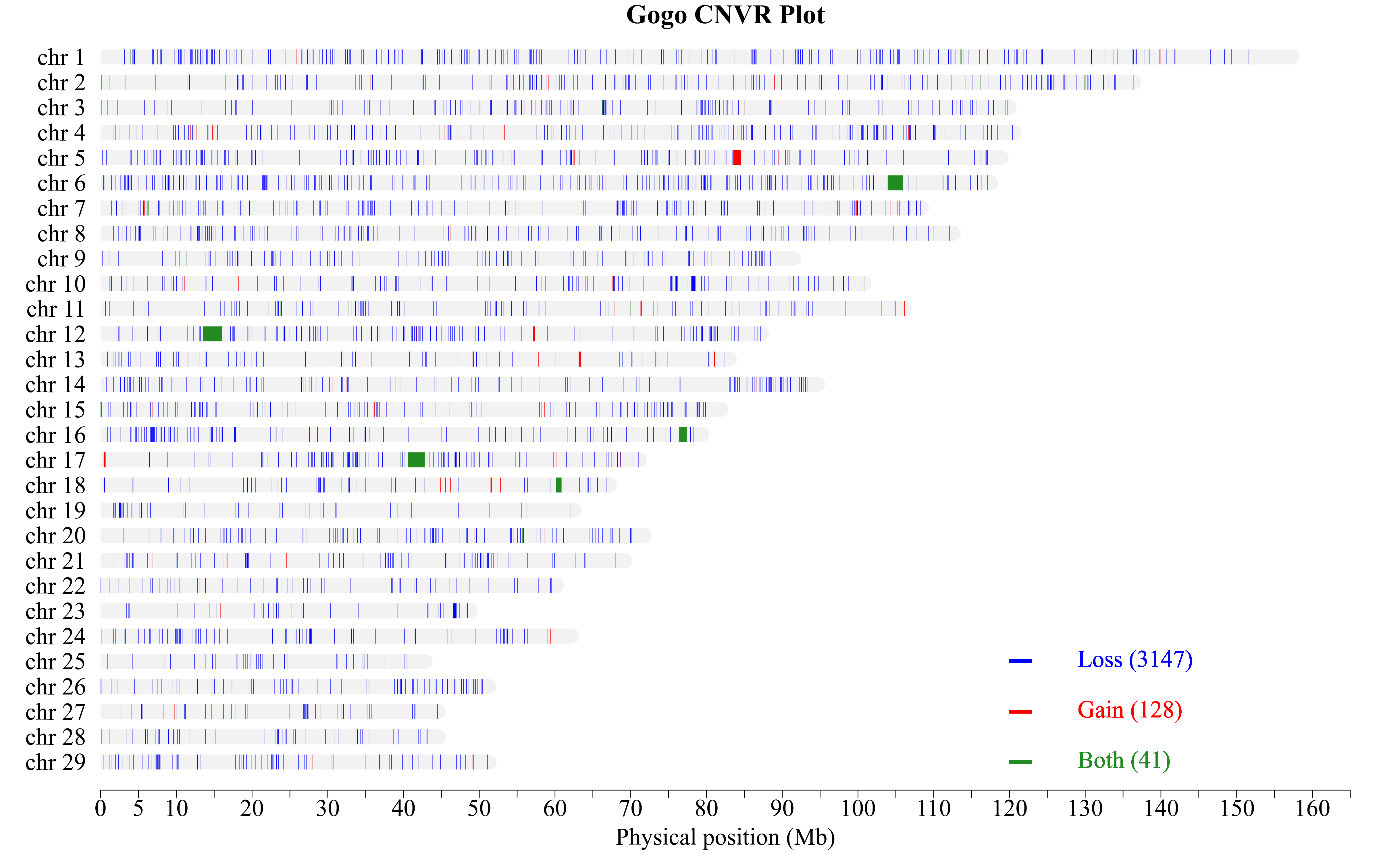


Supplementary Figure 19: CNVR for the Gogo breed


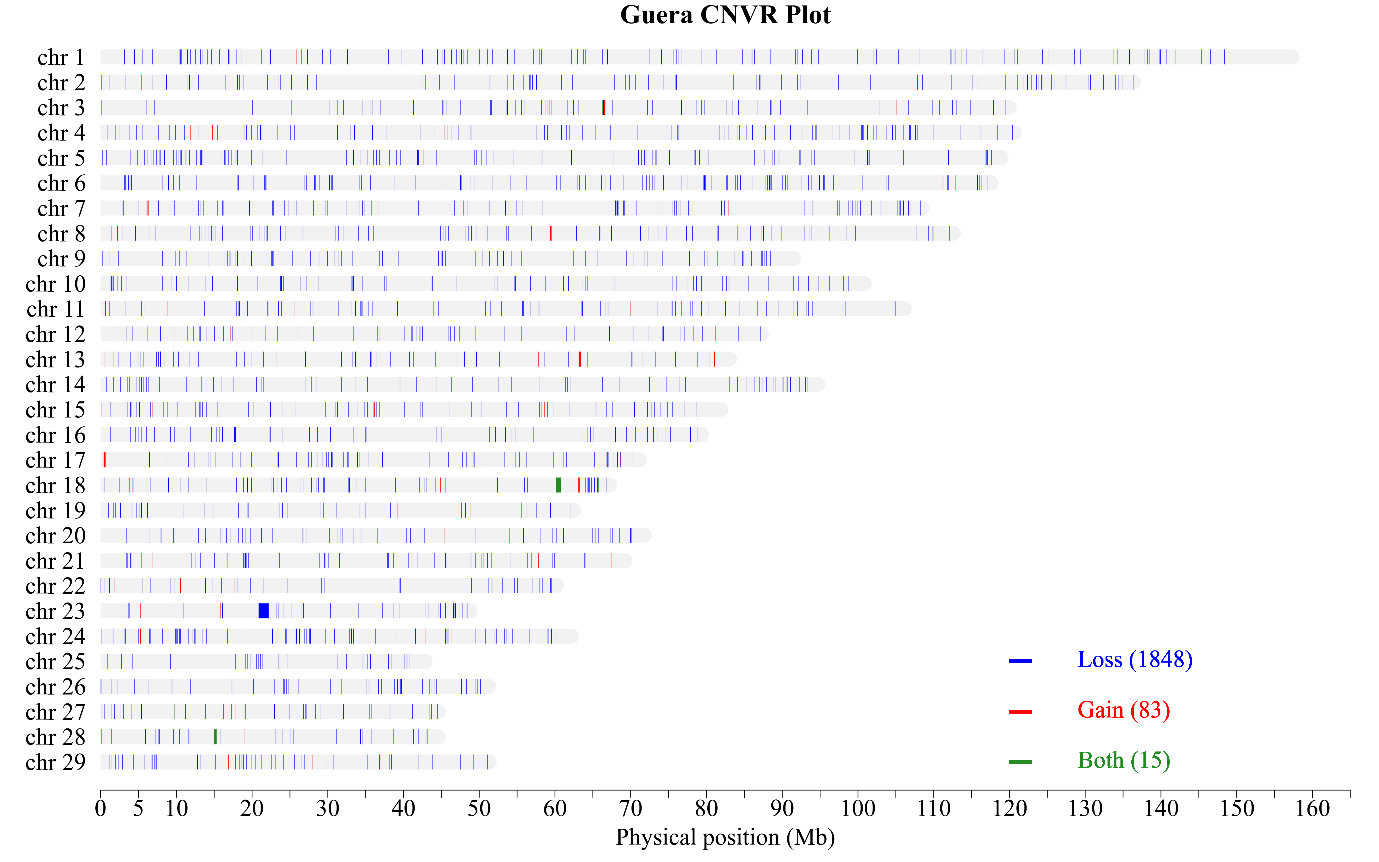


Supplementary Figure 20: CNVR for the Guera breed


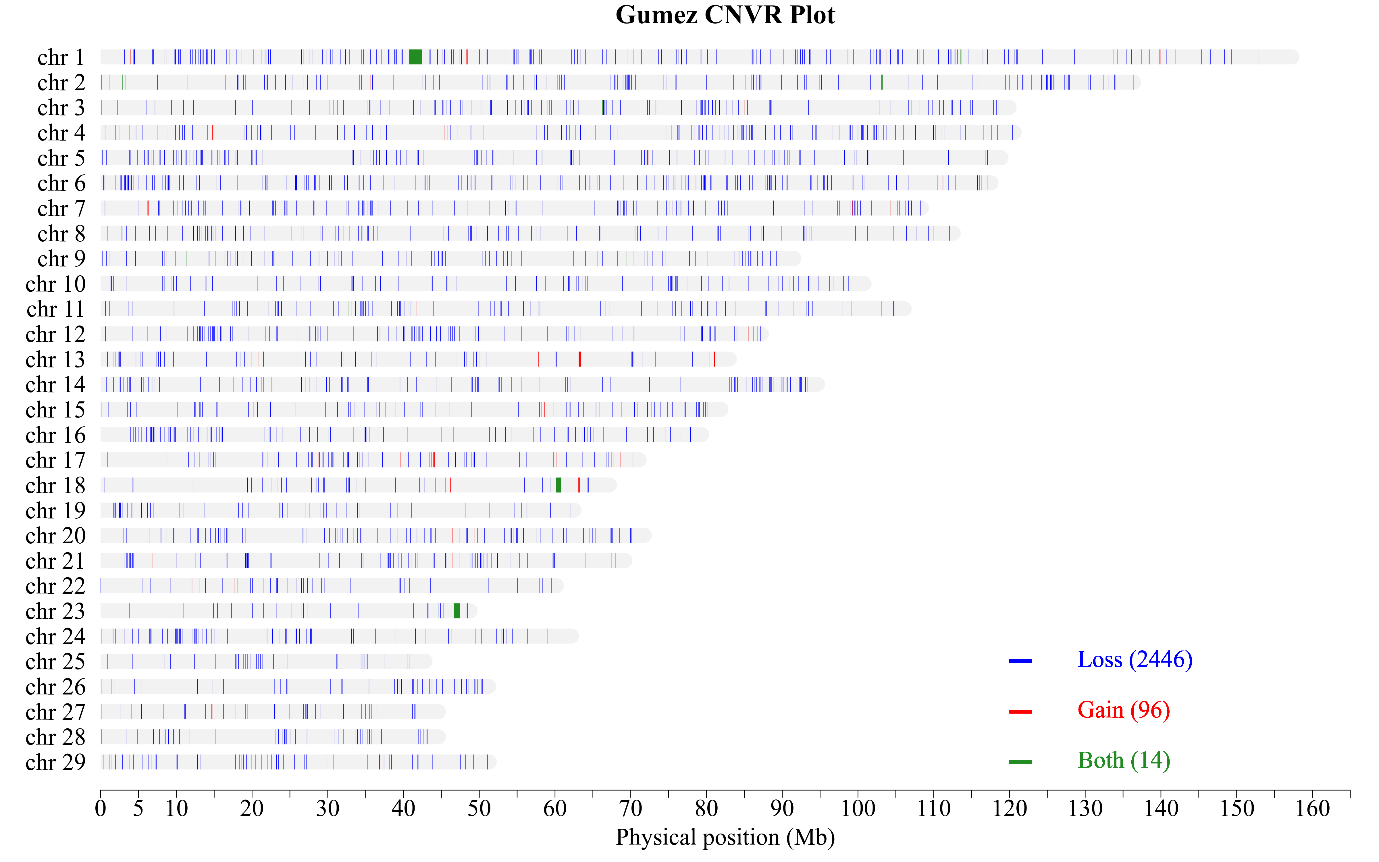


Supplementary Figure 21: CNVR for the Gumez breed


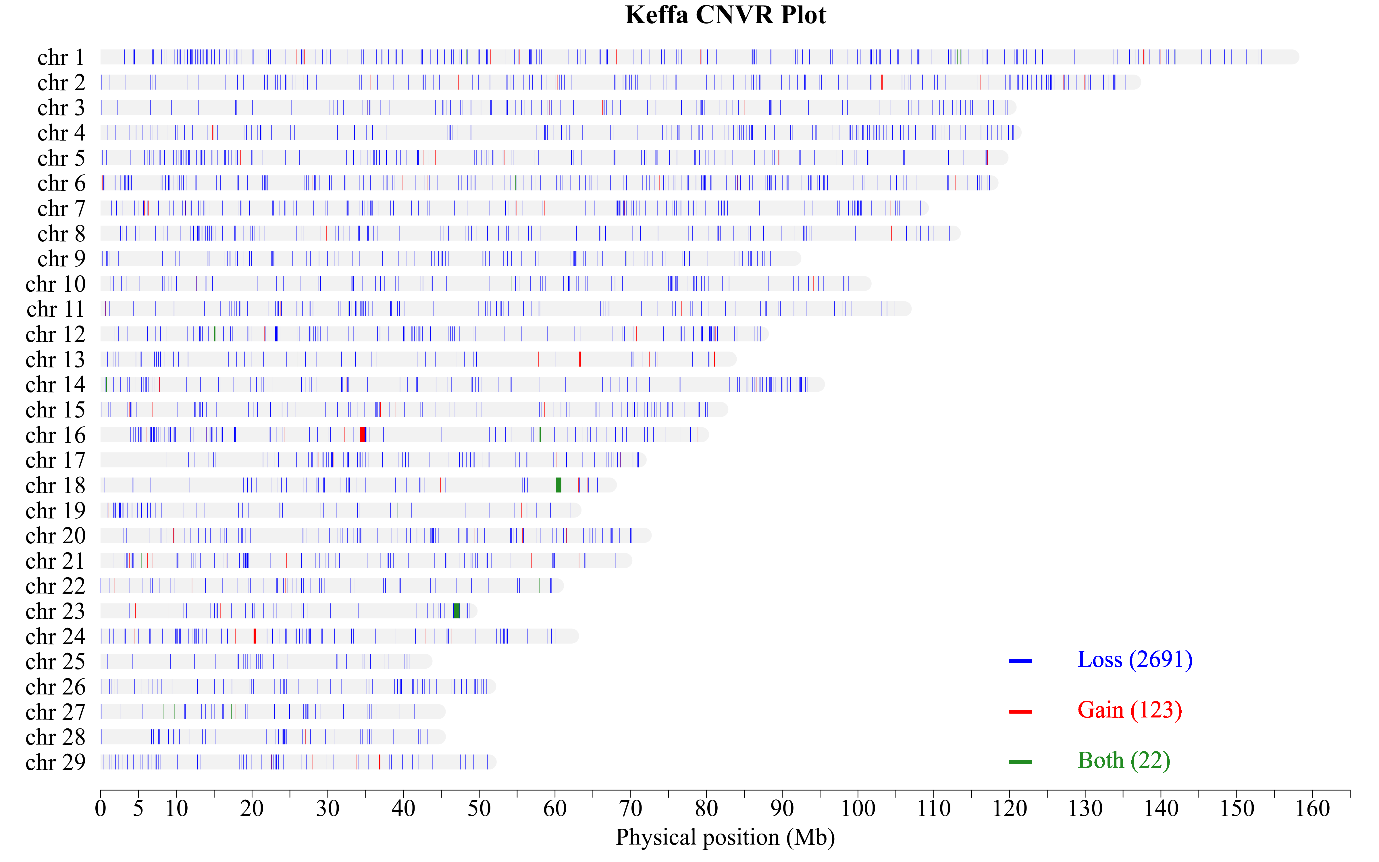


Supplementary Figure 22: CNVR for the Keffa breed


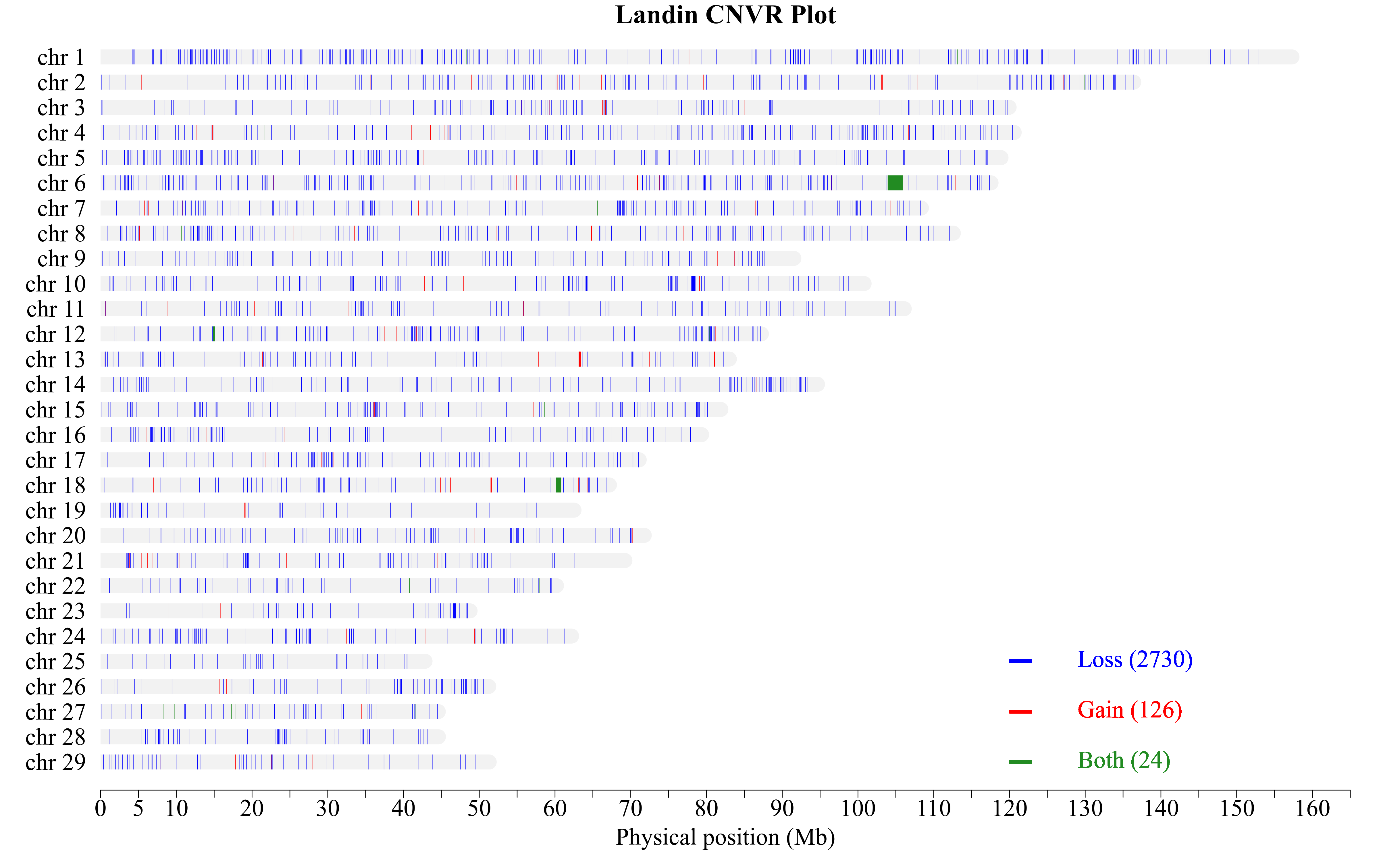


Supplementary Figure 23: CNVR for the Landin breed


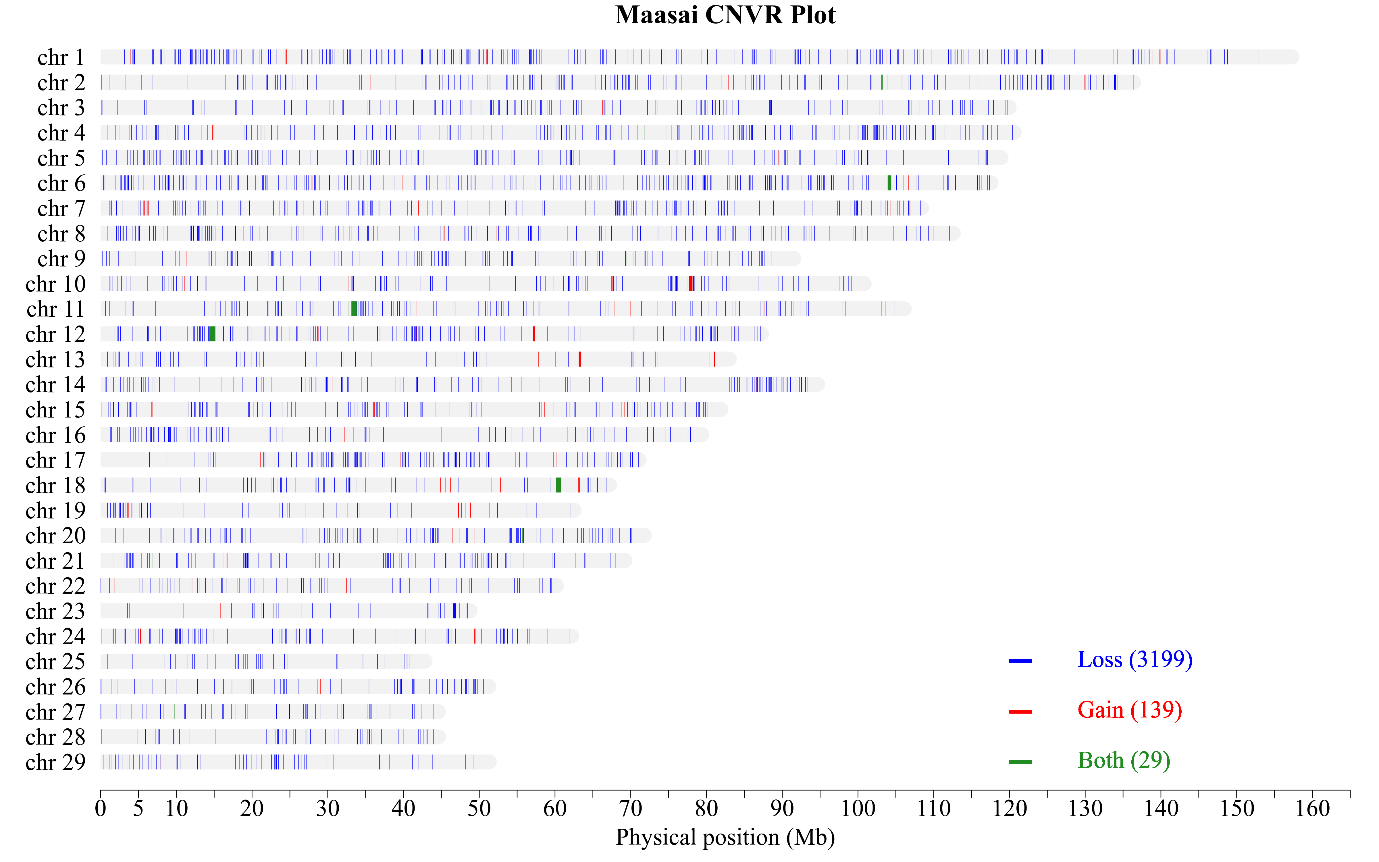


Supplementary Figure 24: CNVR for the Maasai breed


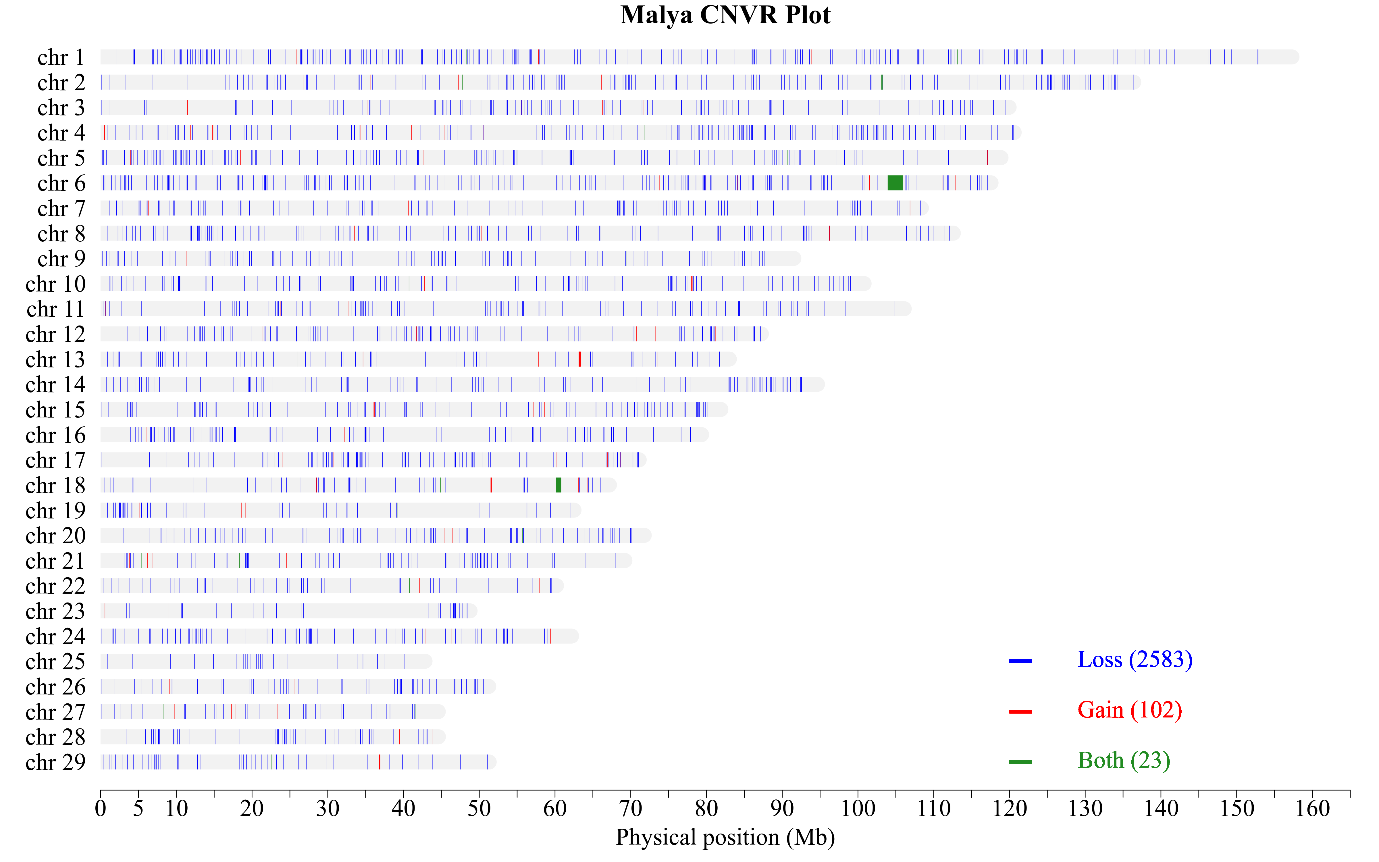


Supplementary Figure 25: CNVR for the Malya breed


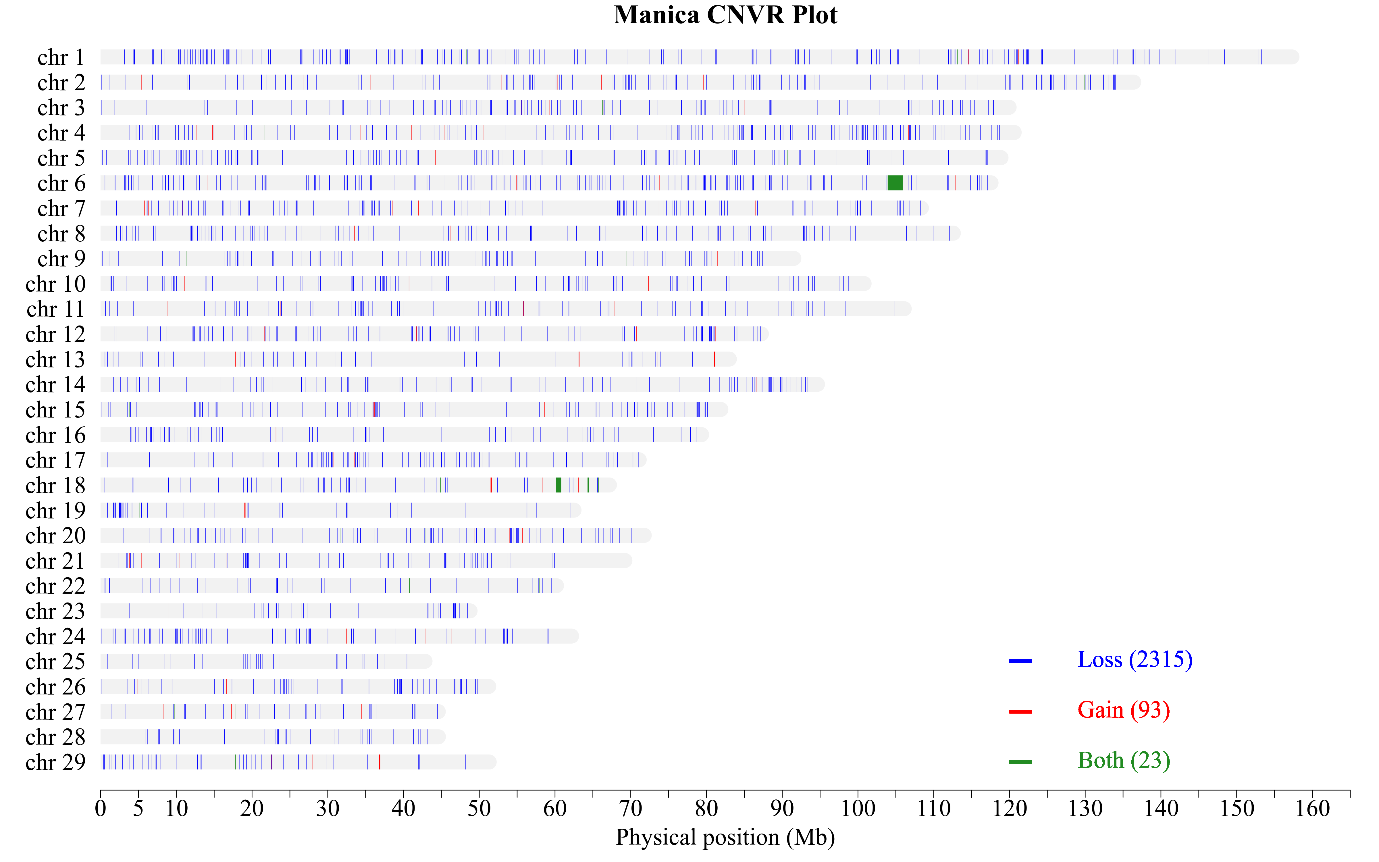


Supplementary Figure 26: CNVR for the Manica breed


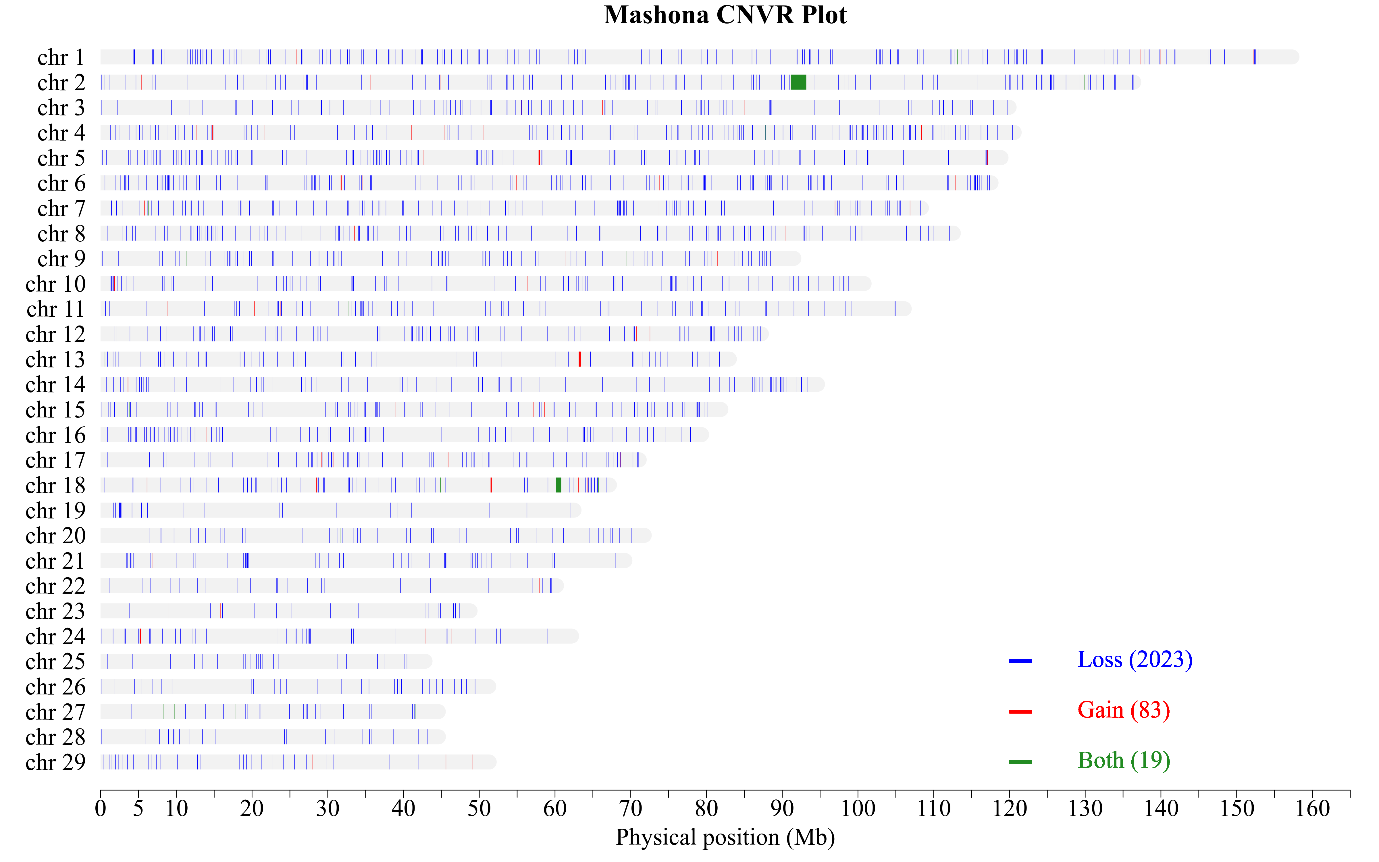


Supplementary Figure 27: CNVR for the Mashona breed


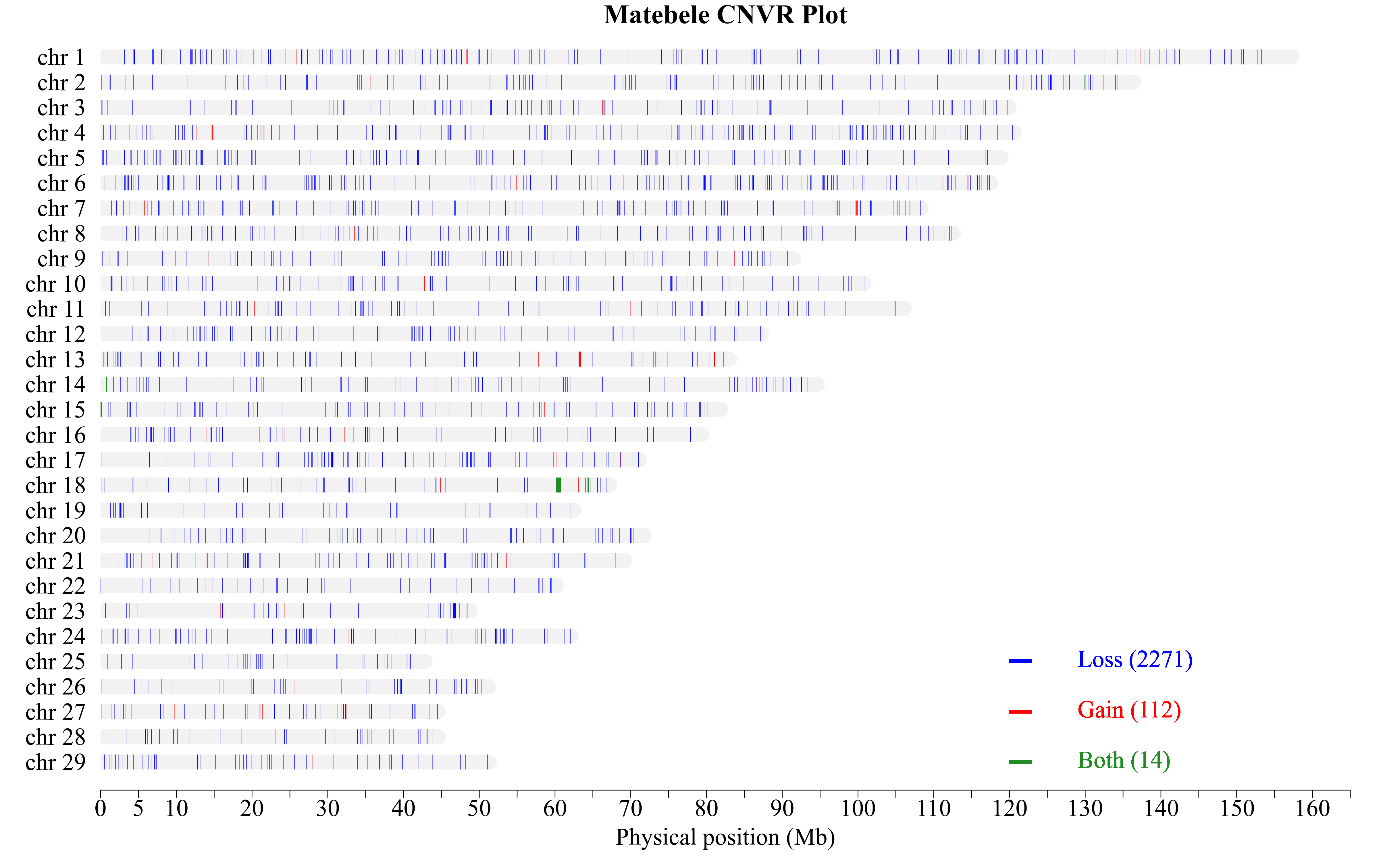


Supplementary Figure 28: CNVR for the Matebele breed


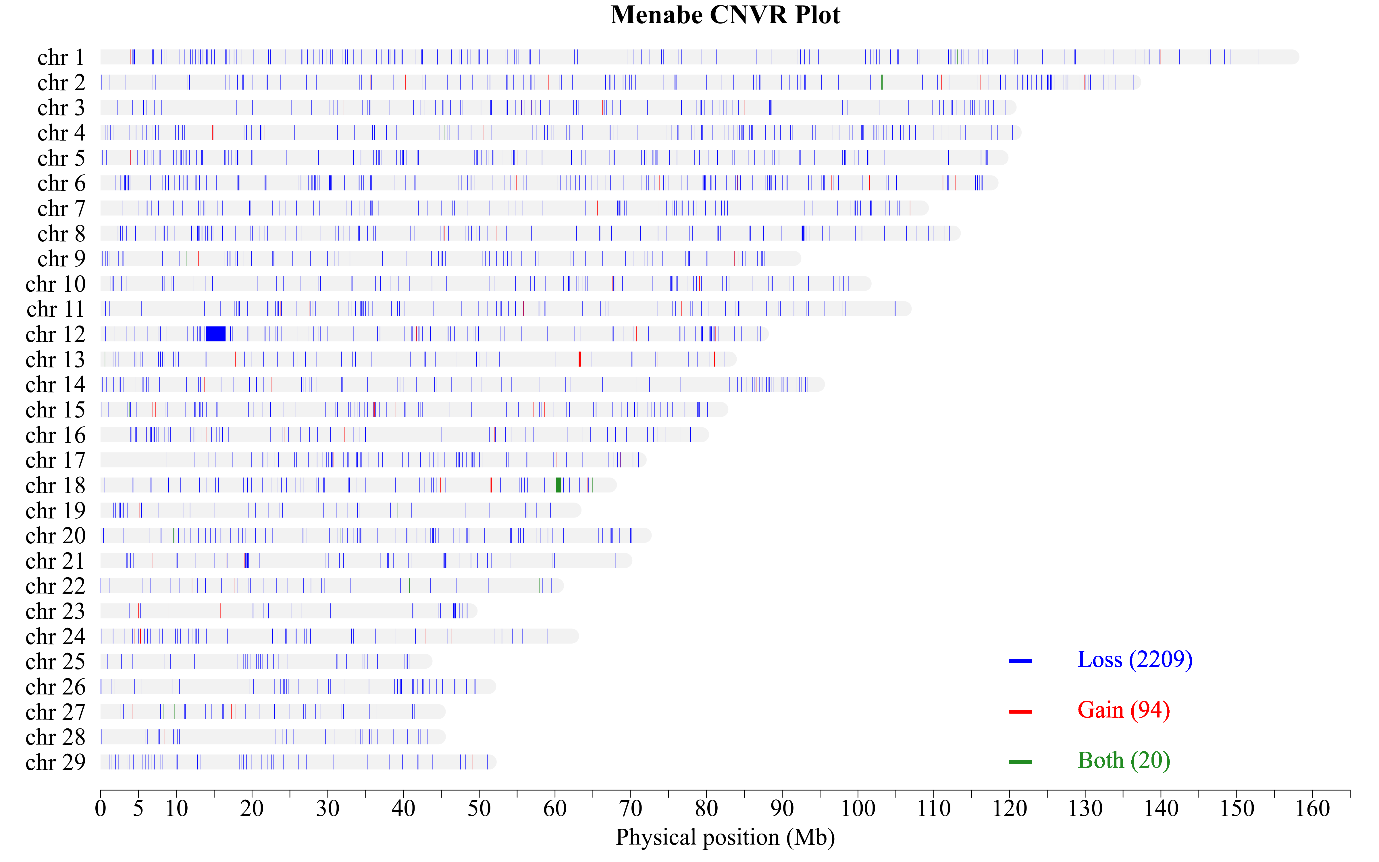


Supplementary Figure 29: CNVR for the Menabe breed


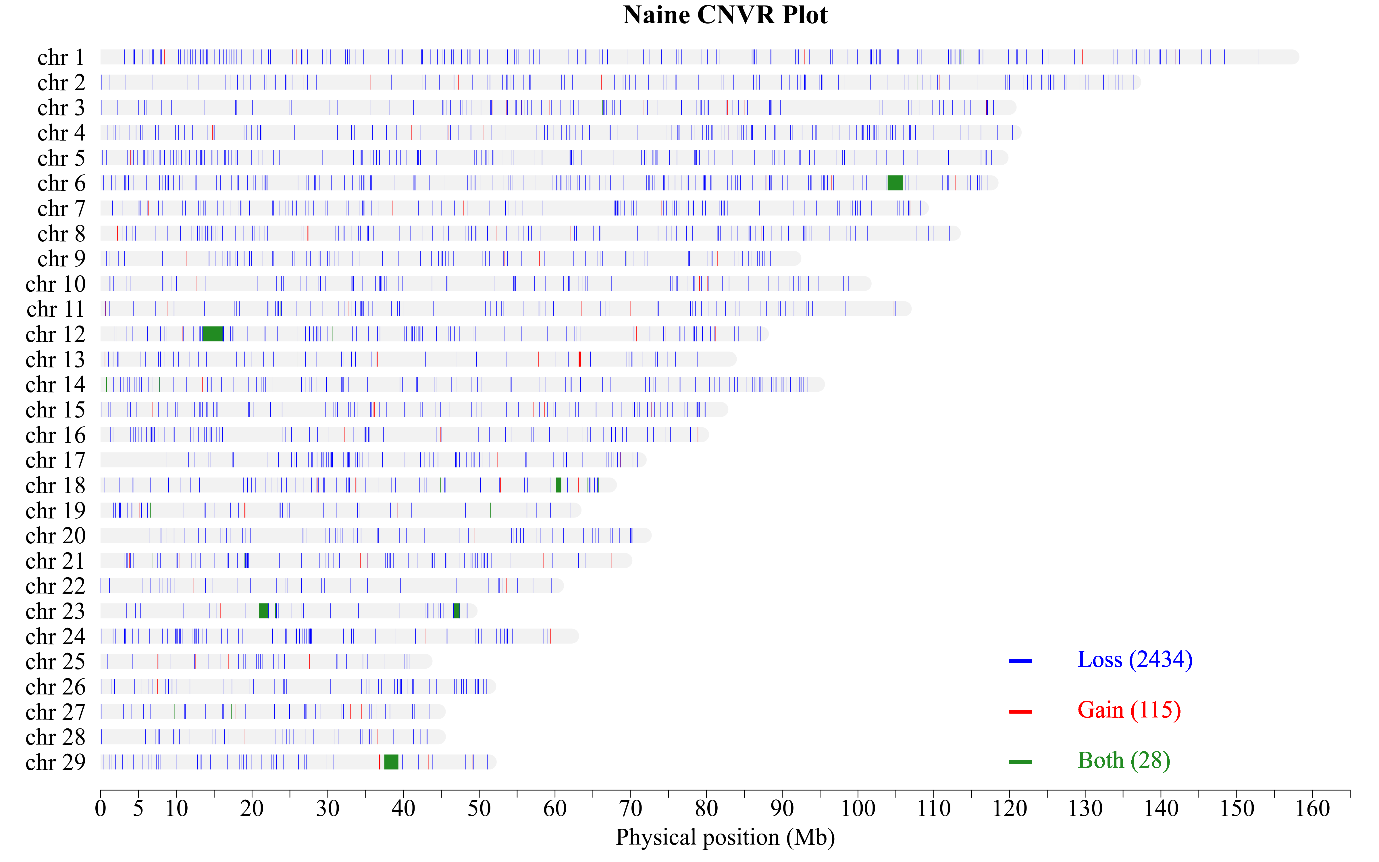


Supplementary Figure 30: CNVR for the Naine breed


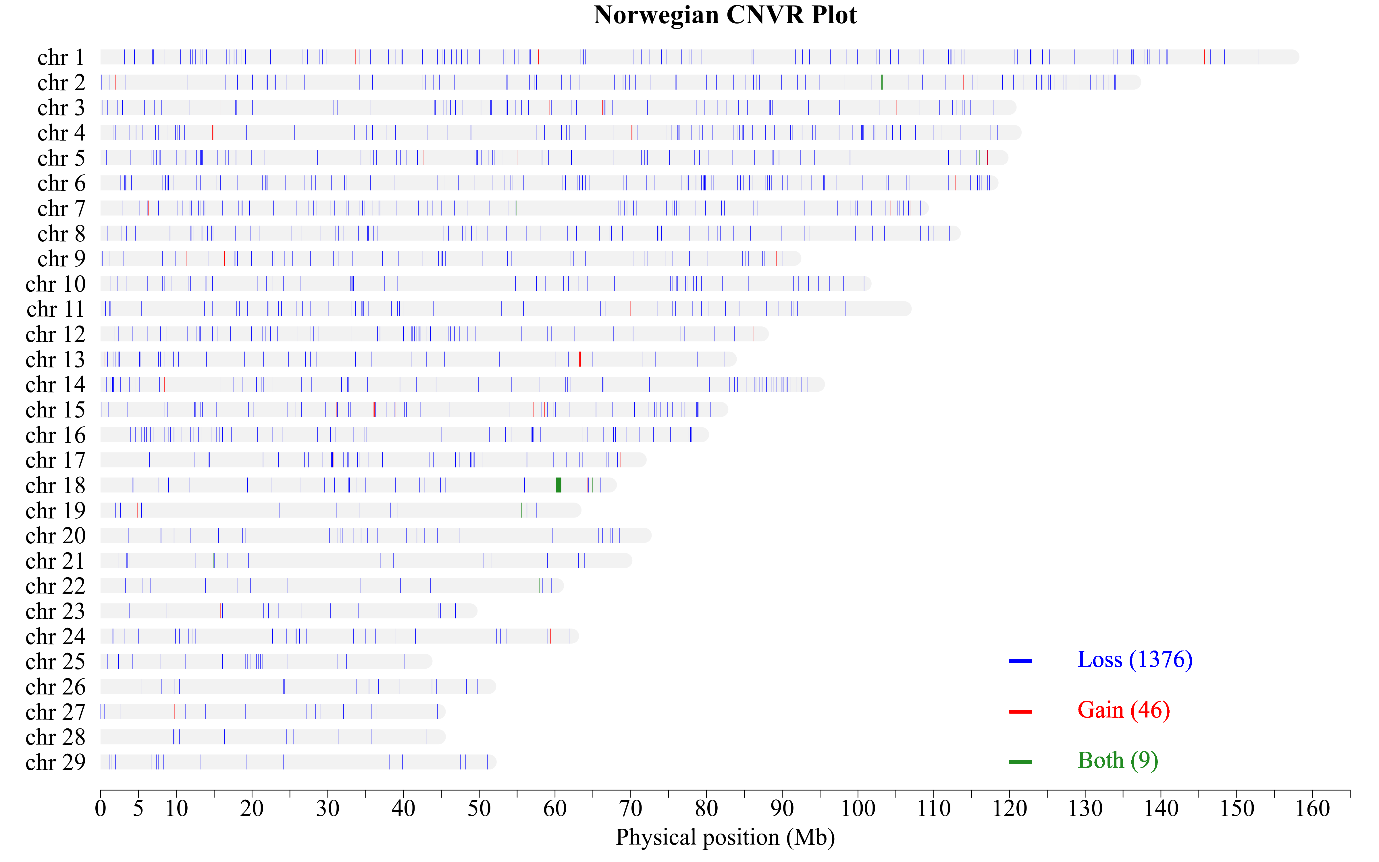


Supplementary Figure 31: CNVR for the Norwegian breed


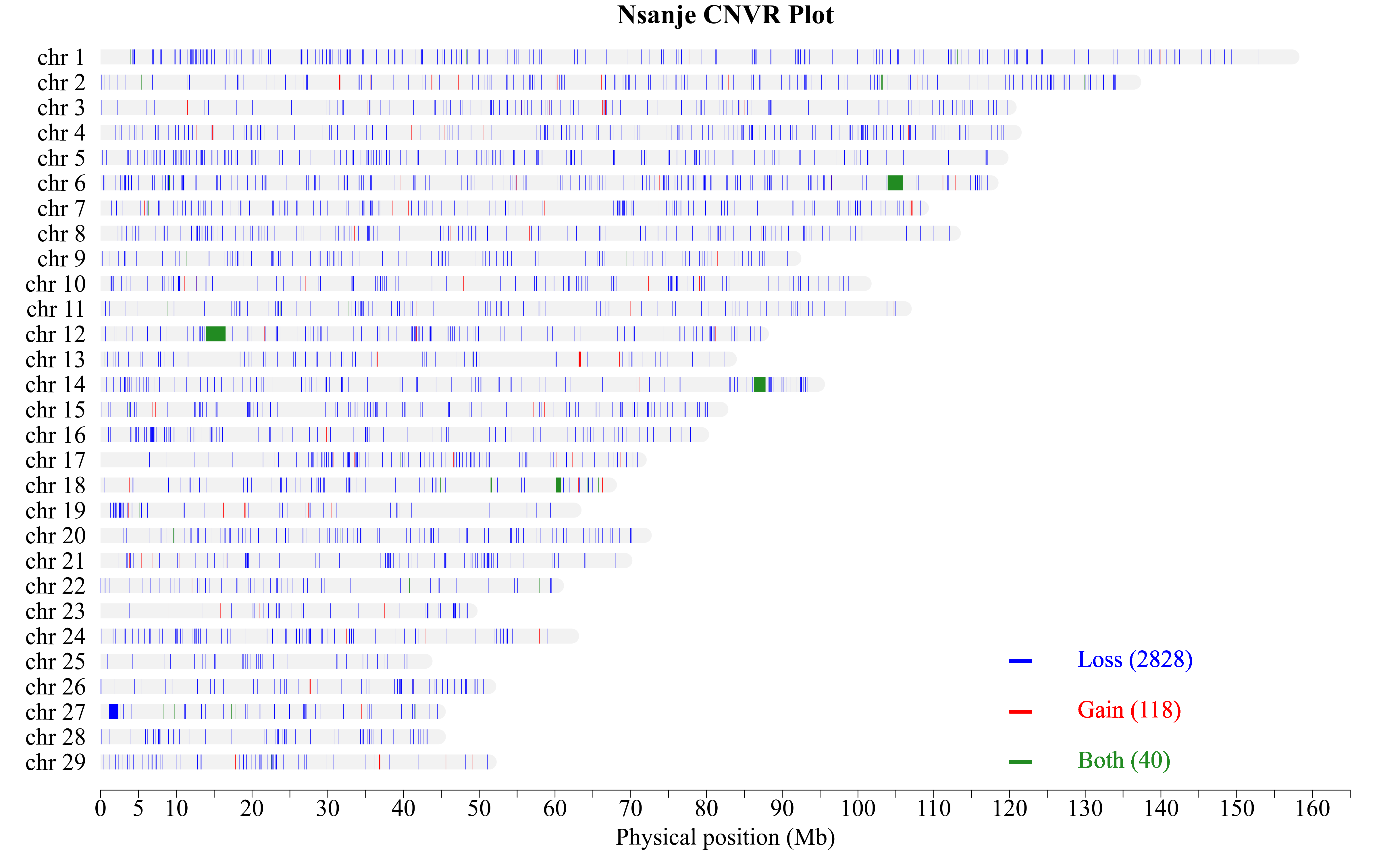


Supplementary Figure 32: CNVR for the Nsanje breed


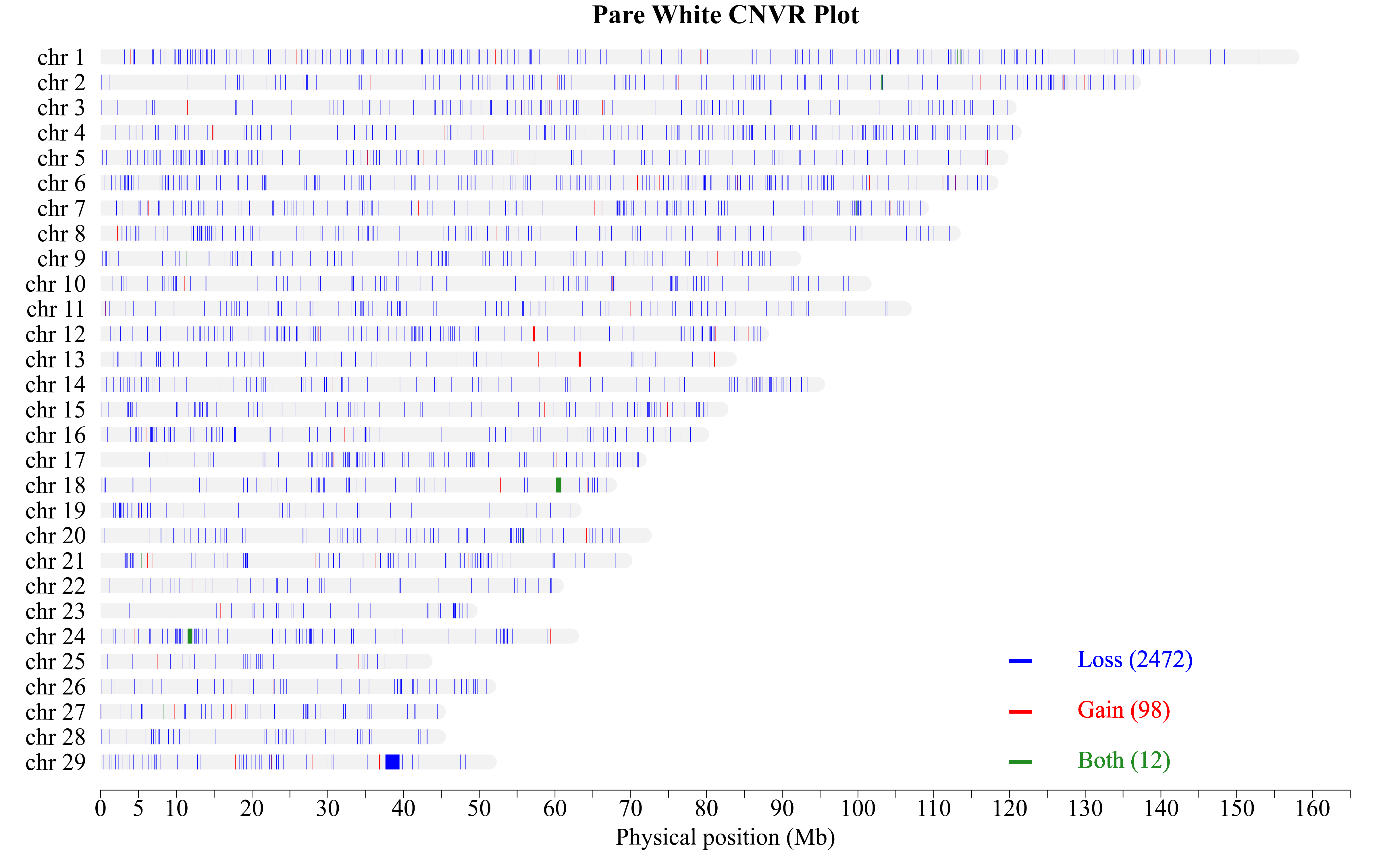


Supplementary Figure 33: CNVR for the Pare White breed


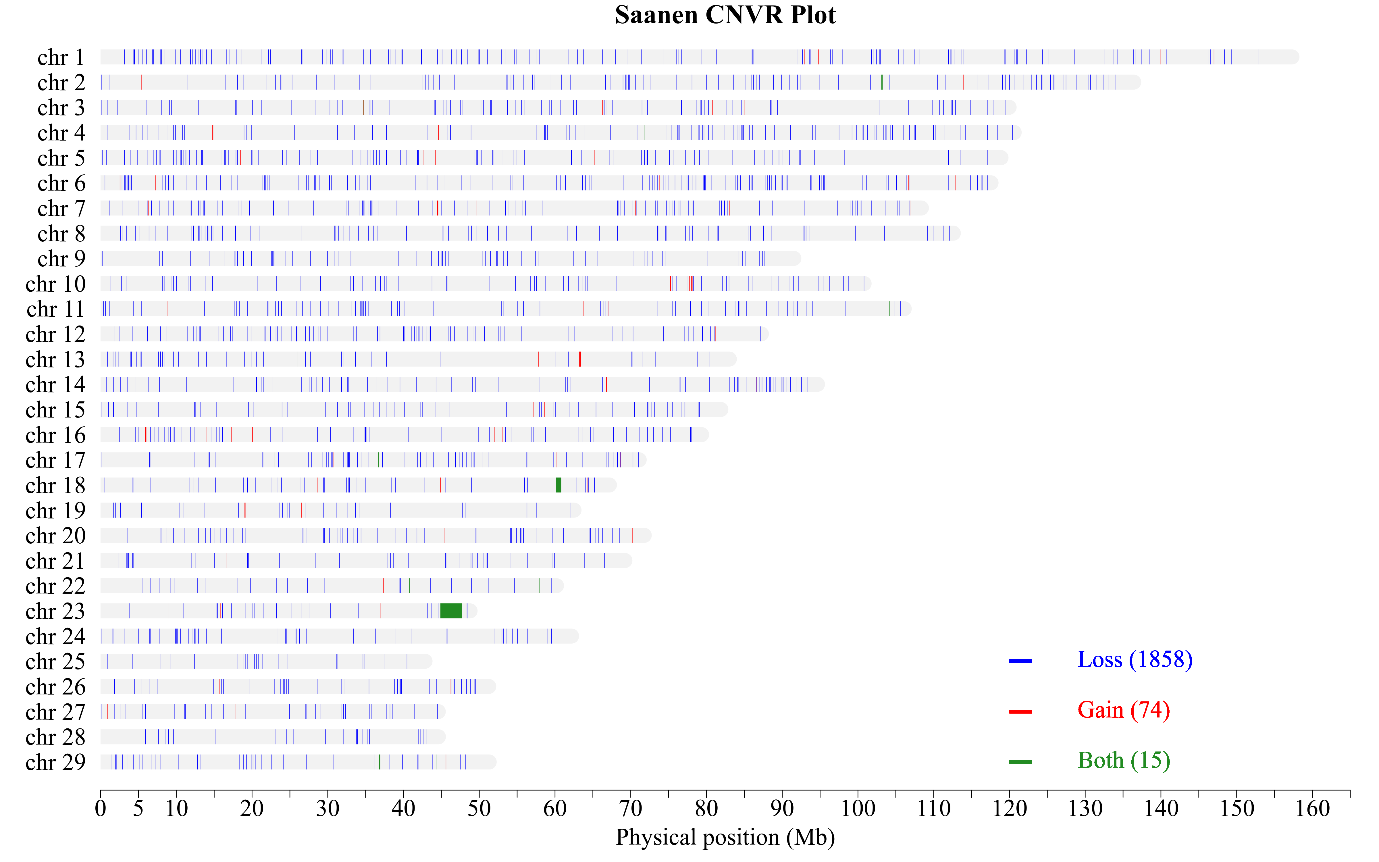


Supplementary Figure 34: CNVR for the Saanen breed


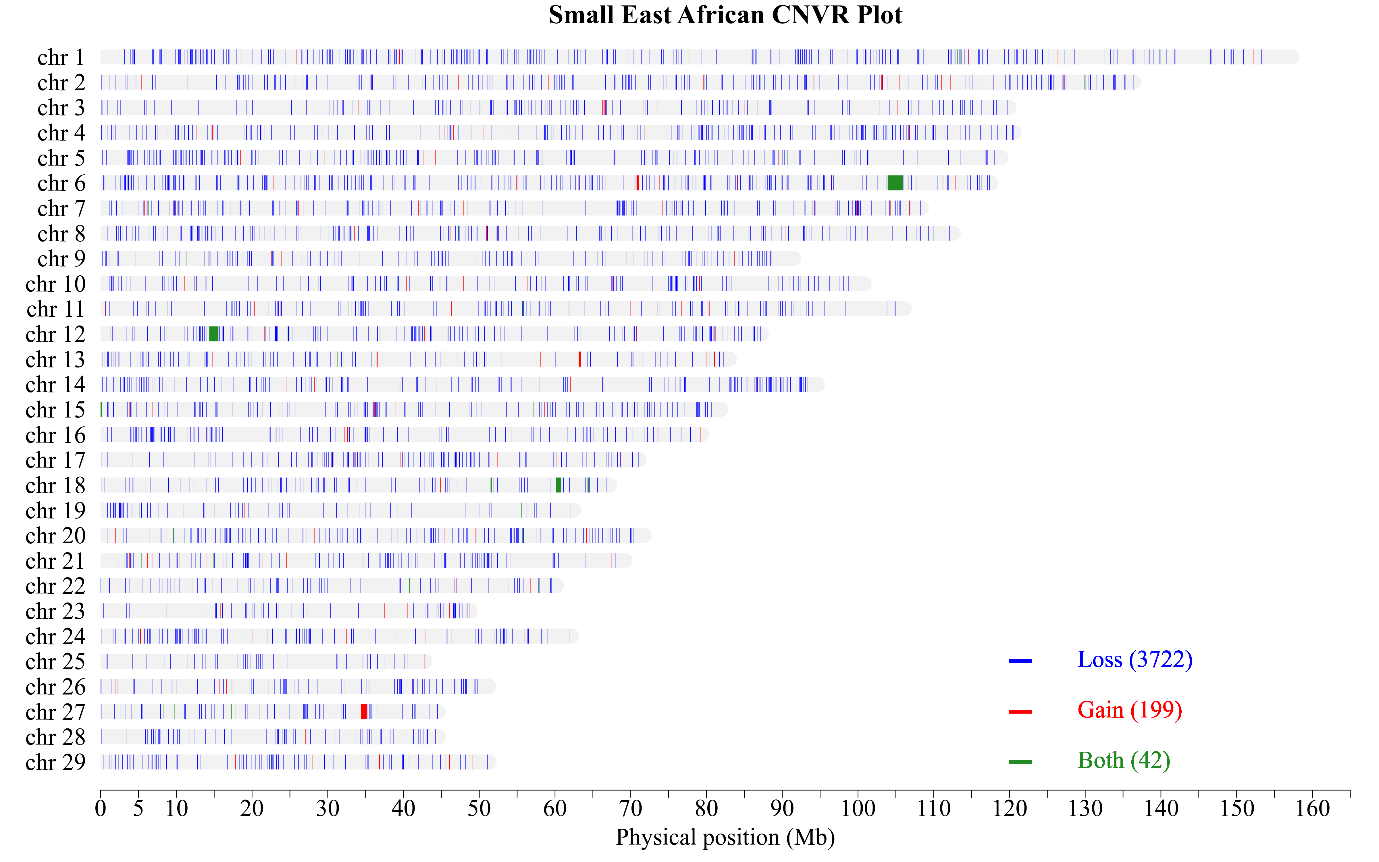


Supplementary Figure 35: CNVR for the Small East African breed


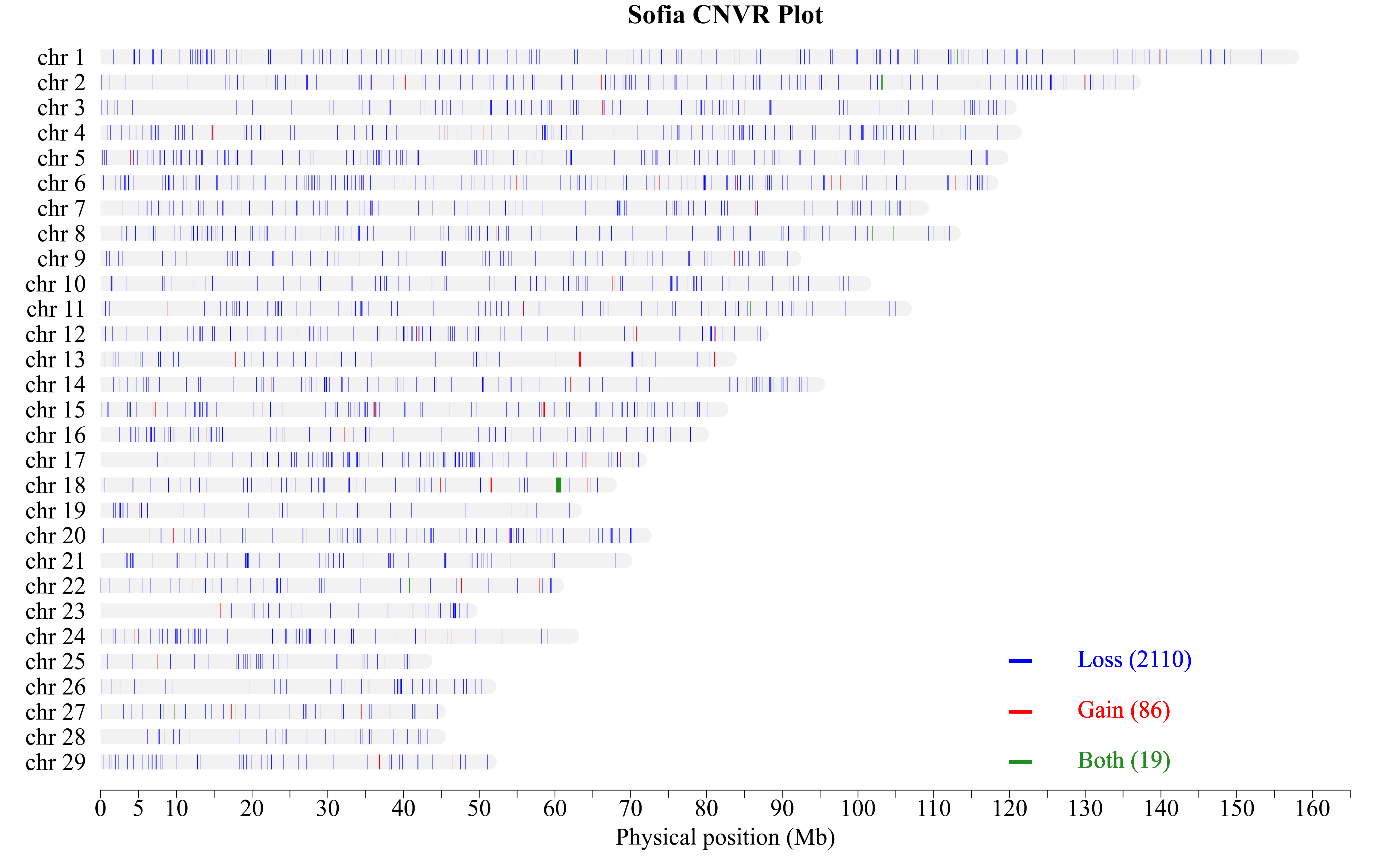


Supplementary Figure 36: CNVR for the Sofia breed


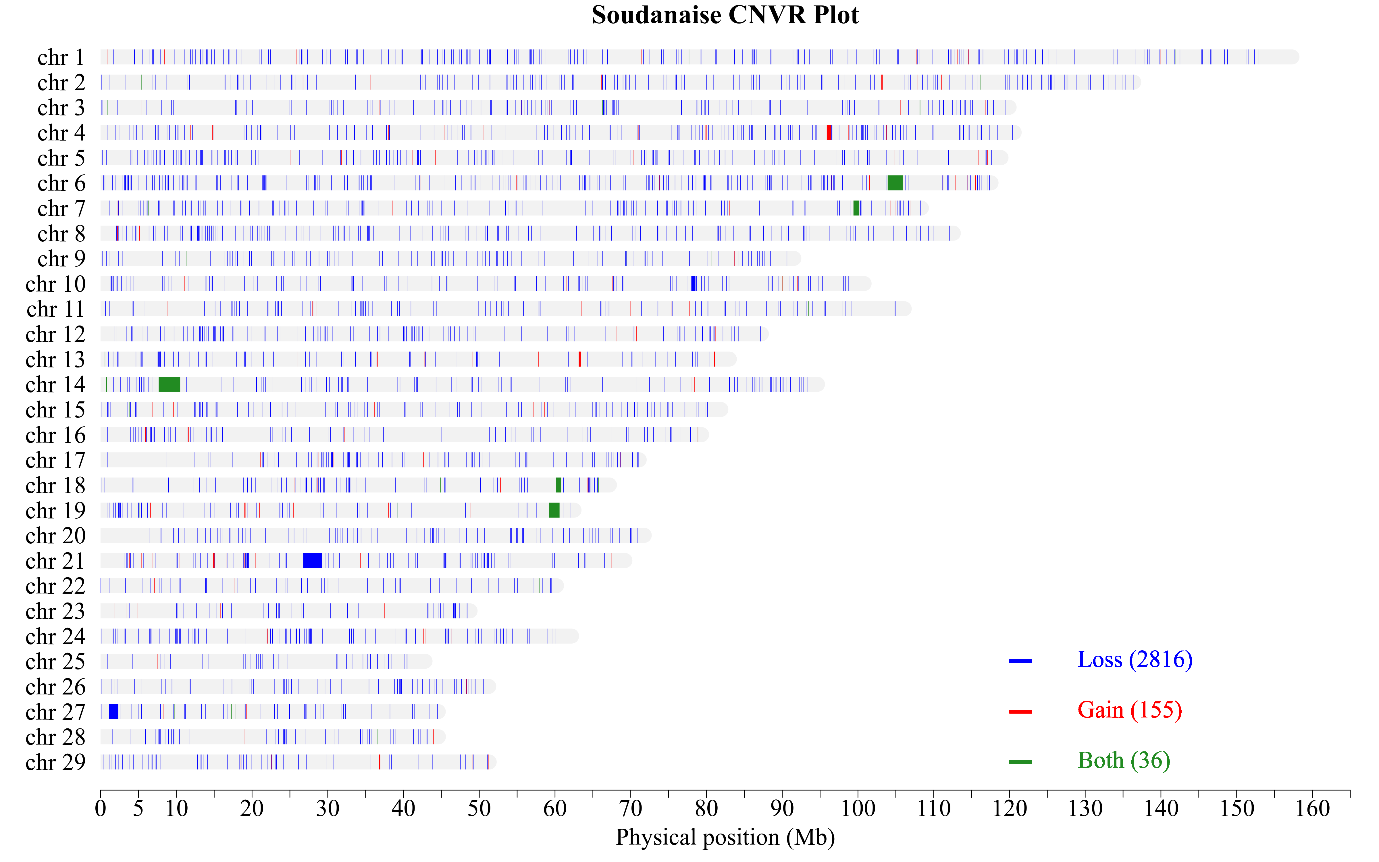


Supplementary Figure 37: CNVR for the Soudanaise breed


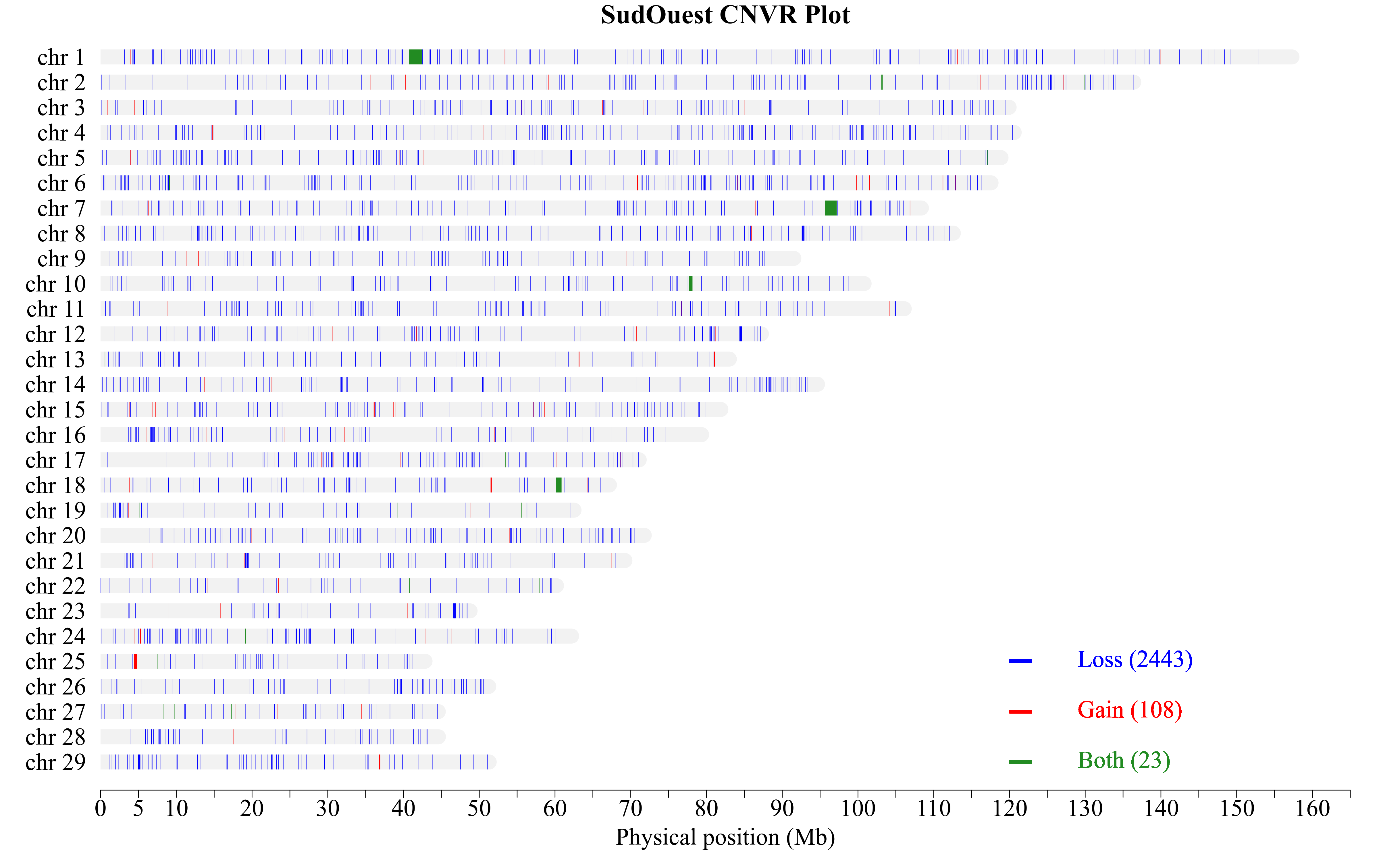


Supplementary Figure 38: CNVR for the SudOuest breed


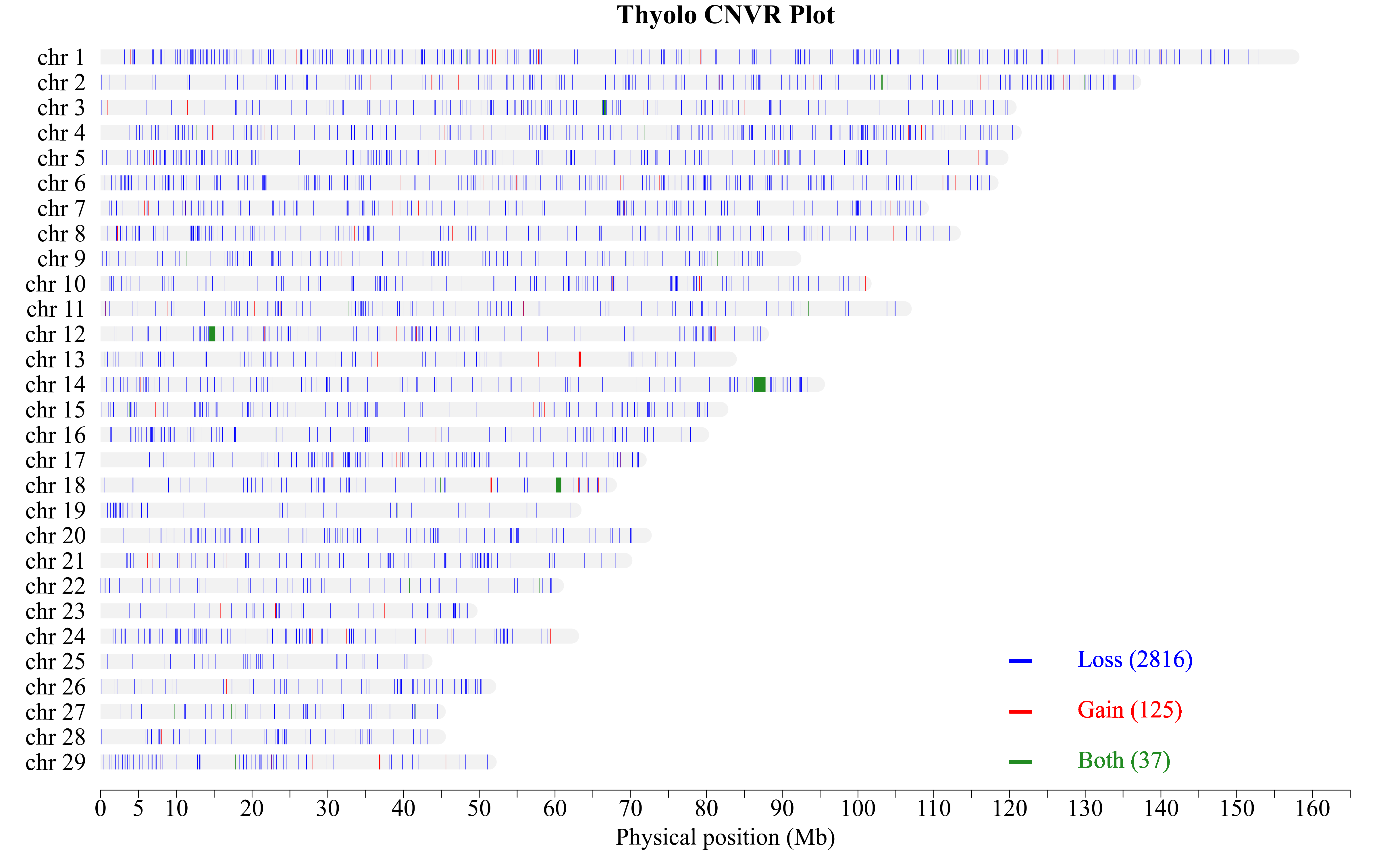


Supplementary Figure 39: CNVR for the Thyolo breed


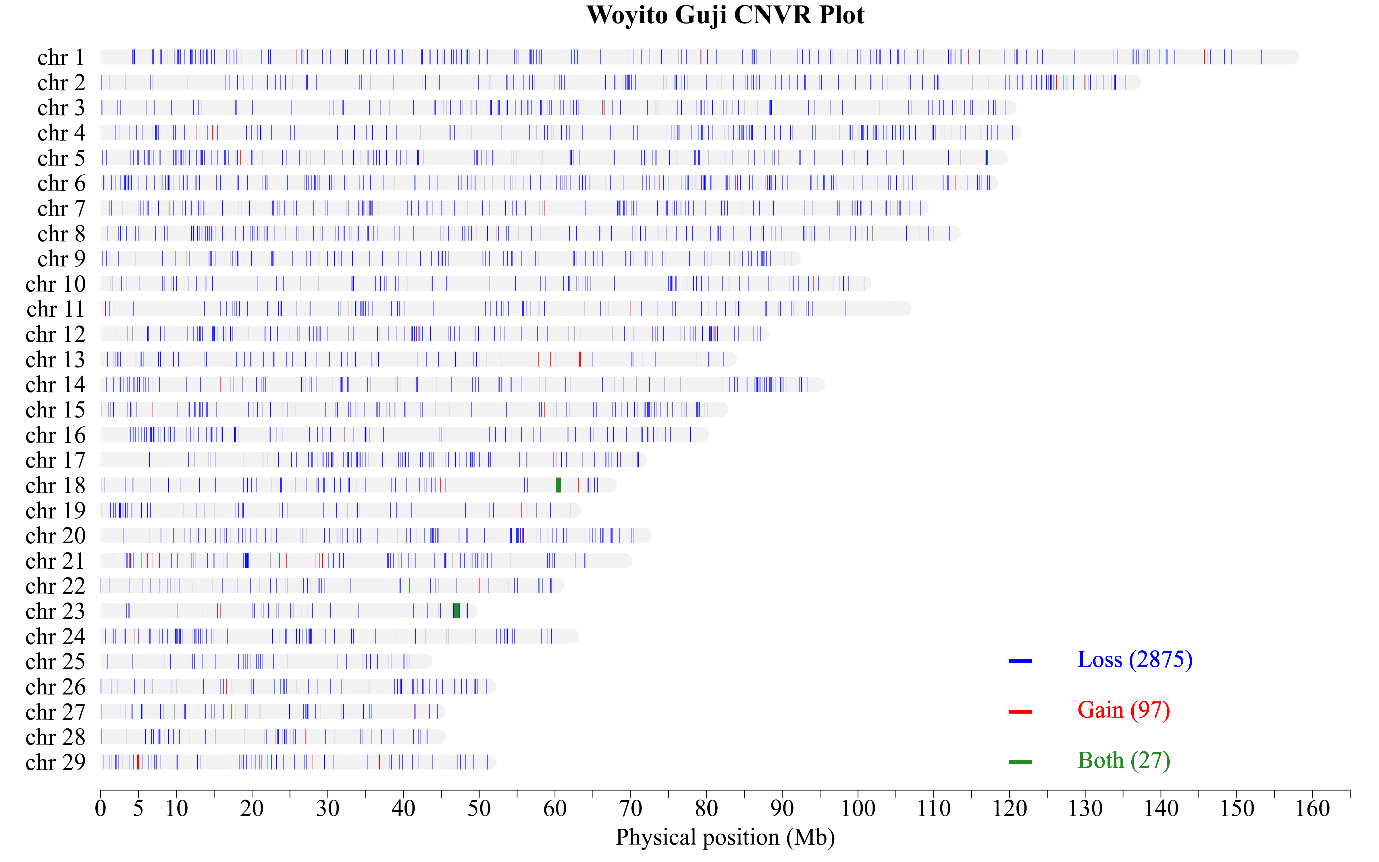


Supplementary Figure 40: CNVR for the Woyito Guji breed


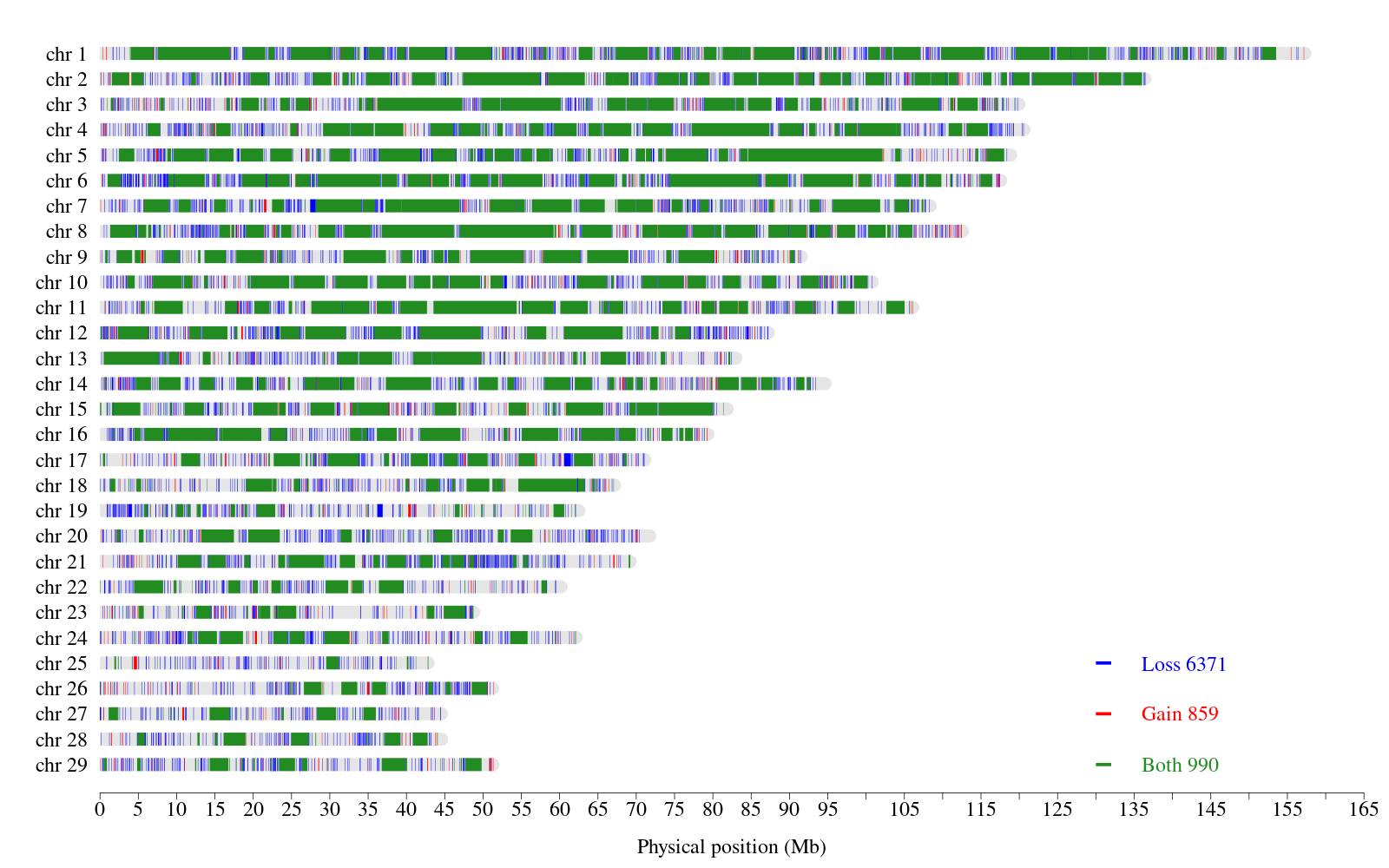
Supplementary Figure 41: Global CNVR with variable SV calling parameters

Imprecise SV included

Lower cut-off length of 50 bp applied before merging Manta and Lumpy SV

No SV filters (PE = SR = 0)

Upper cut-off length: 3 Mb


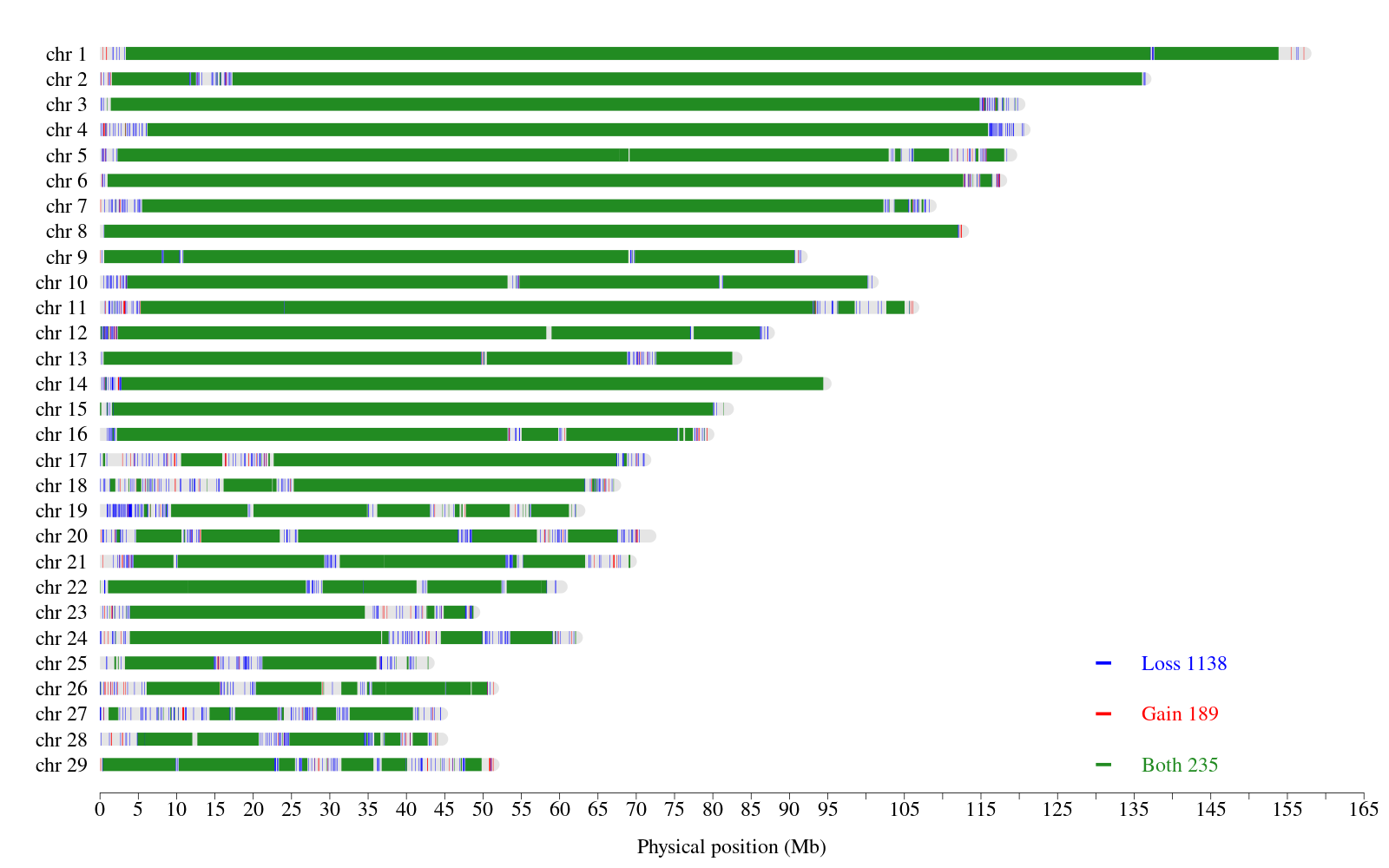
Supplementary Figure 42: Global CNVR with variable SV calling parameters

Imprecise SV included

Lower cut-off length of 50 bp applied before merging Manta and Lumpy SV

No SV filters (PE = SR = 0)

Upper cut-off length: 10 Mb


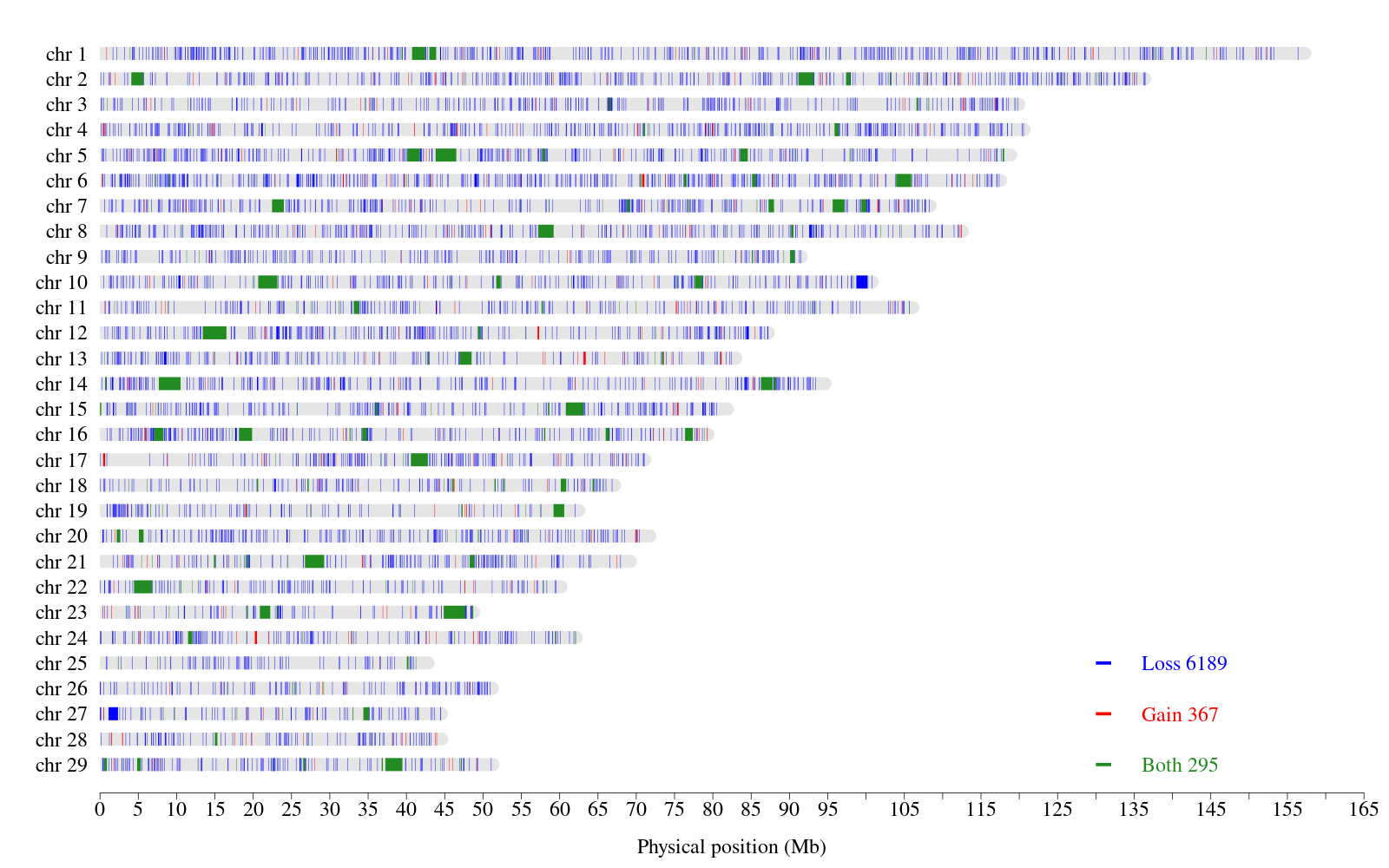
Supplementary Figure 43: Global CNVR with variable SV calling parameters

Imprecise SV included

Lower cut-off length of 50 bp applied before merging Manta and Lumpy SV

Medium SV filters (PE = SR = 3)

Upper cut-off length: 3 Mb


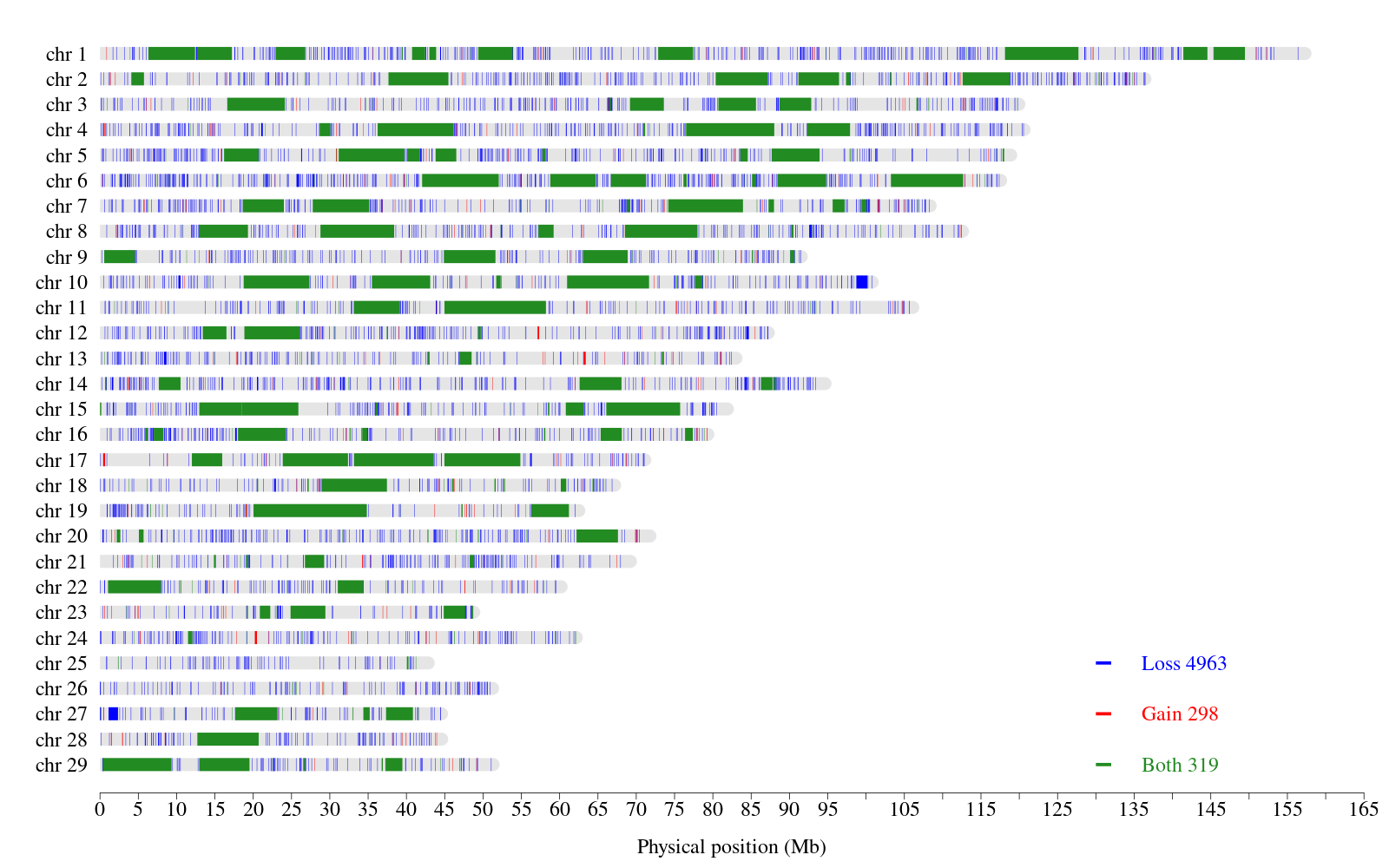
Supplementary Figure 44: Global CNVR with variable SV calling parameters

Imprecise SV included

Lower cut-off length of 50 bp applied before merging Manta and Lumpy SV

Medium SV filters (PE = SR = 3)

Upper cut-off length: 10 Mb


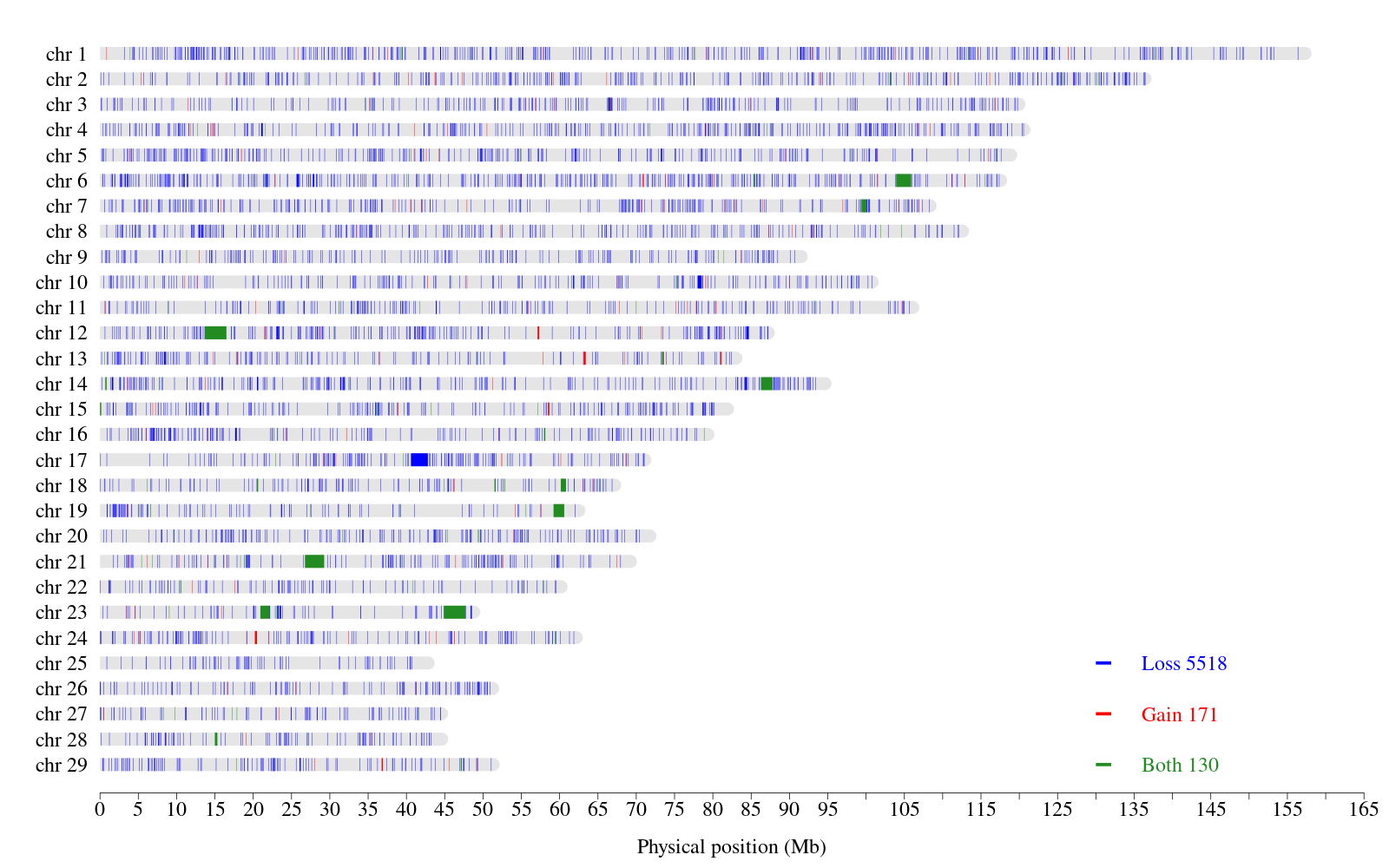
Supplementary Figure 45: Global CNVR with variable SV calling parameters

Imprecise SV included

Lower cut-off length of 50 bp applied before merging Manta and Lumpy SV

Stringent SV filters (PE = SR = 5)

Upper cut-off length: 3 Mb


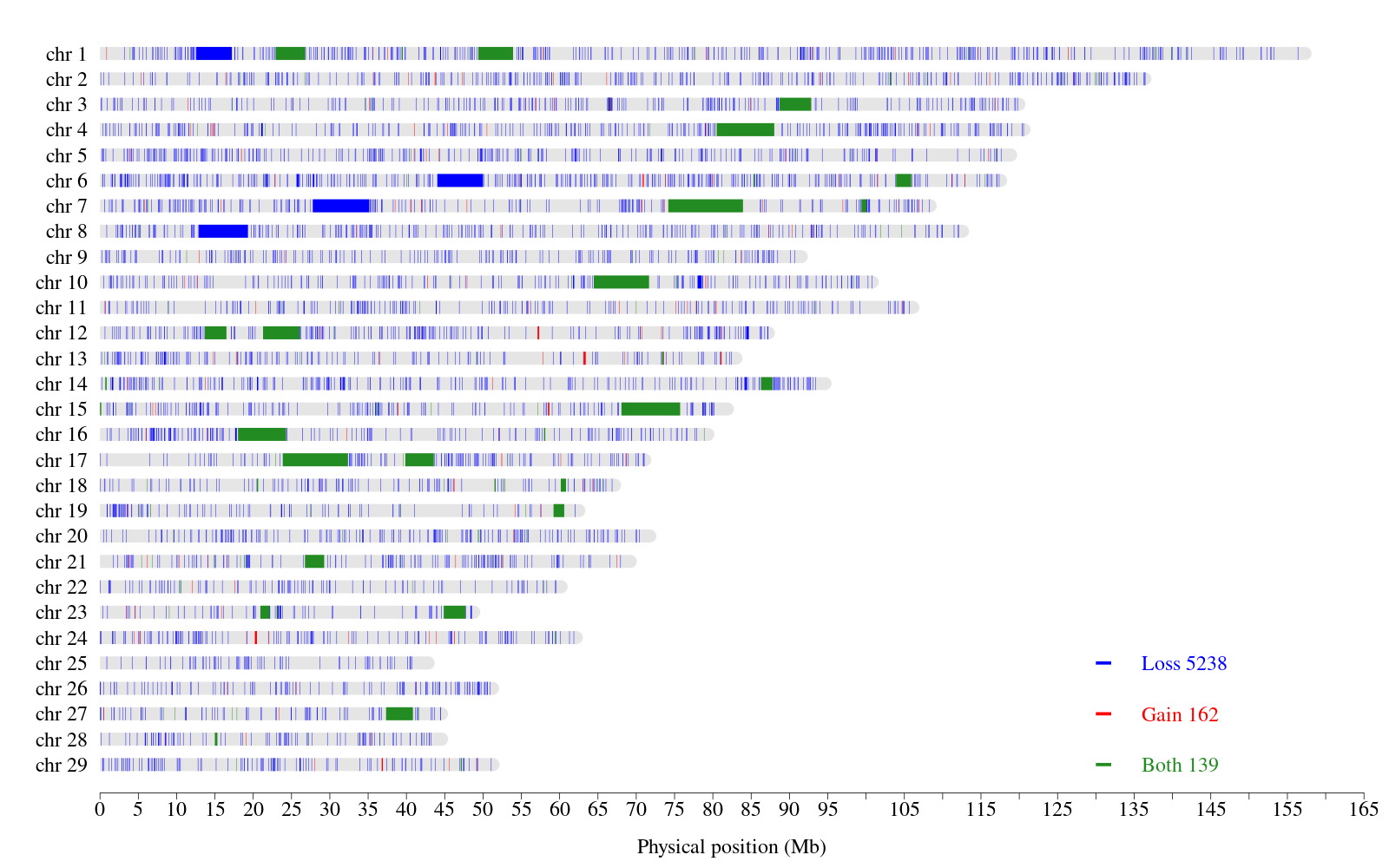
Supplementary Figure 46: Global CNVR with variable SV calling parameters

Imprecise SV included

Lower cut-off length of 50 bp applied before merging Manta and Lumpy SV

Stringent SV filters (PE = SR = 3)

Upper cut-off length: 10 Mb


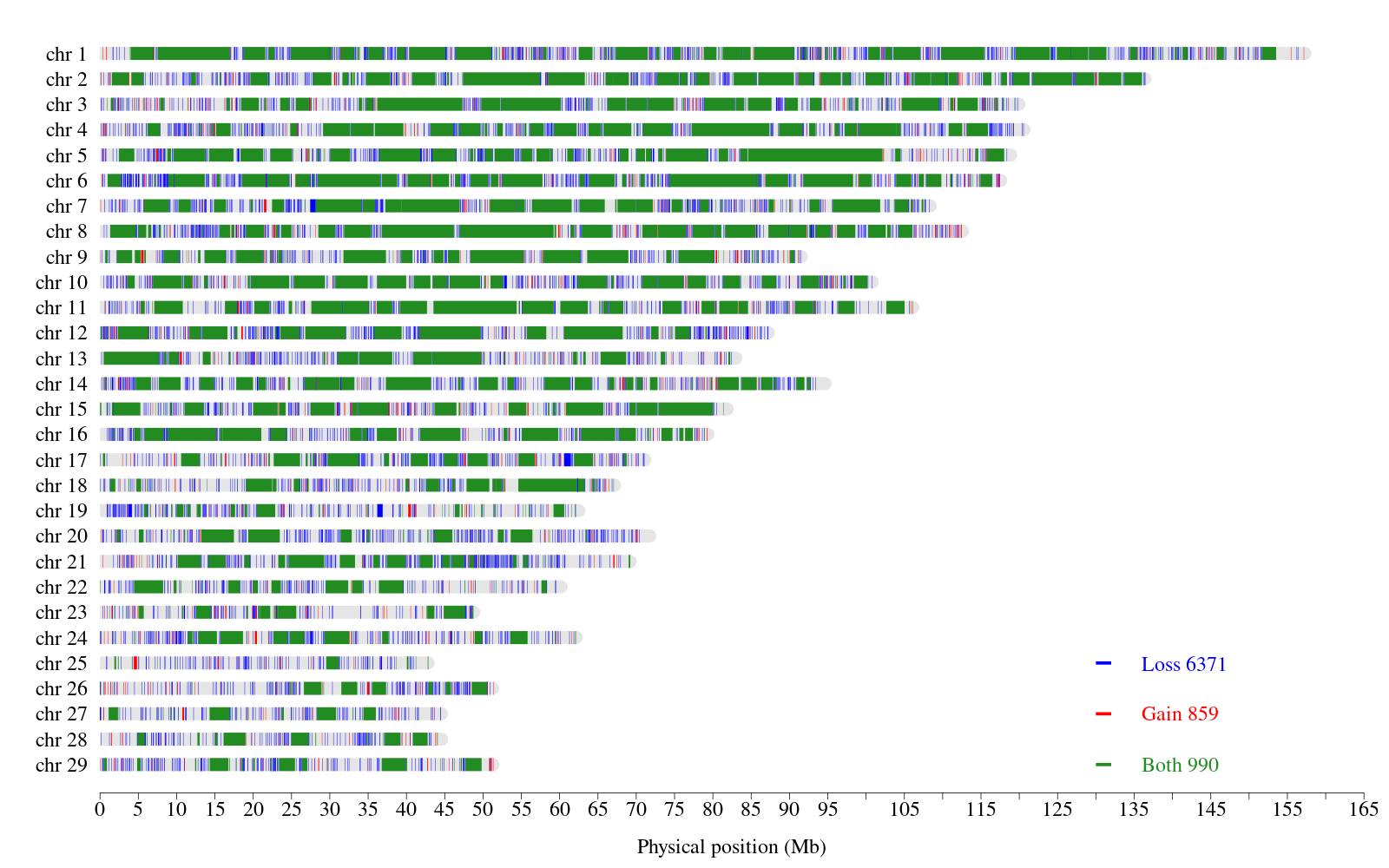
Supplementary Figure 47: Global CNVR with variable SV calling parameters

Imprecise SV included

Lower cut-off length of 50 bp applied after merging Manta and Lumpy SV

No SV filters (PE = SR = 0)

Upper cut-off length: 3 Mb


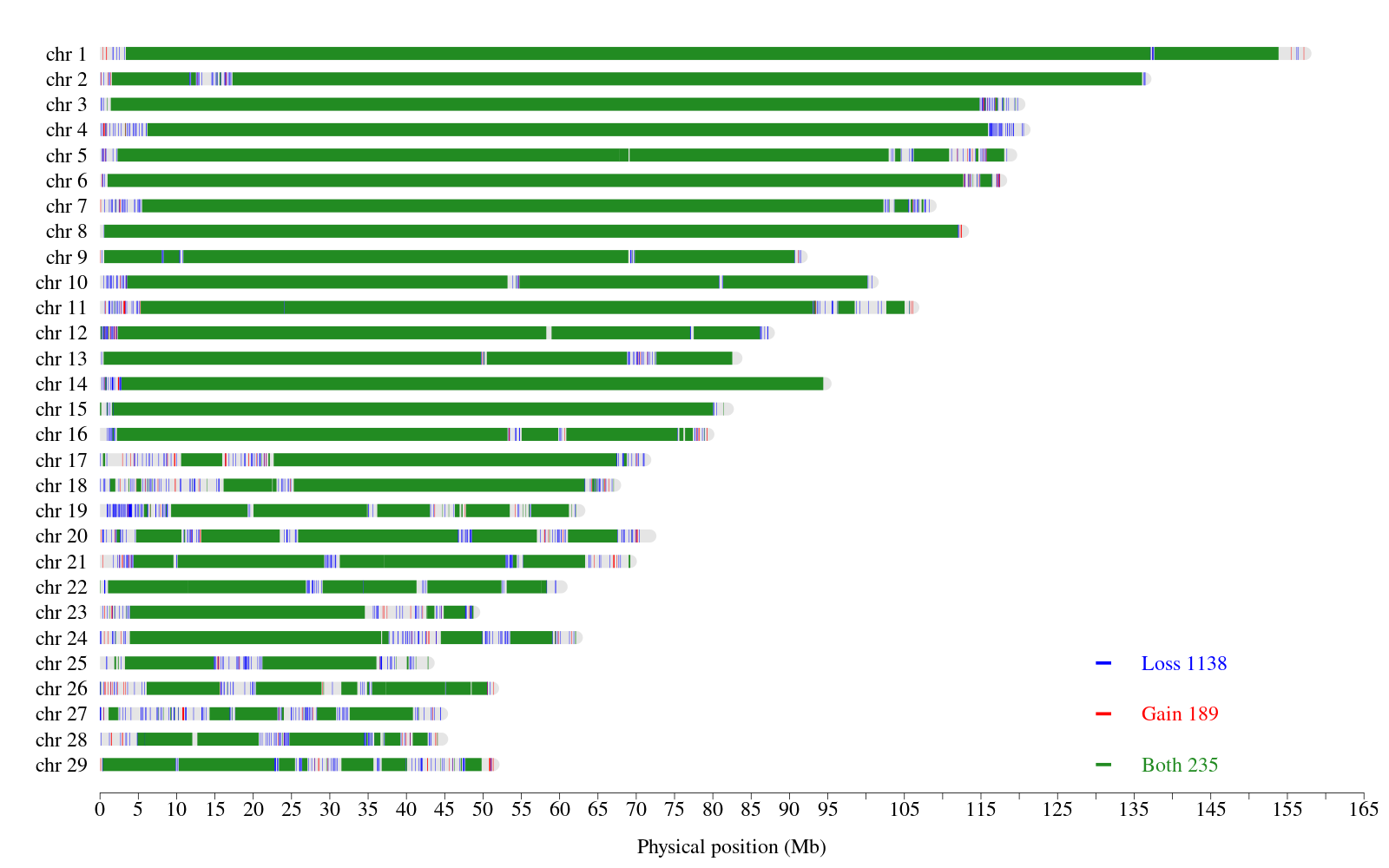
Supplementary Figure 48: Global CNVR with variable SV calling parameters

Imprecise SV included

Lower cut-off length of 50 bp applied after merging Manta and Lumpy SV

No SV filters (PE = SR = 0)

Upper cut-off length: 10 Mb


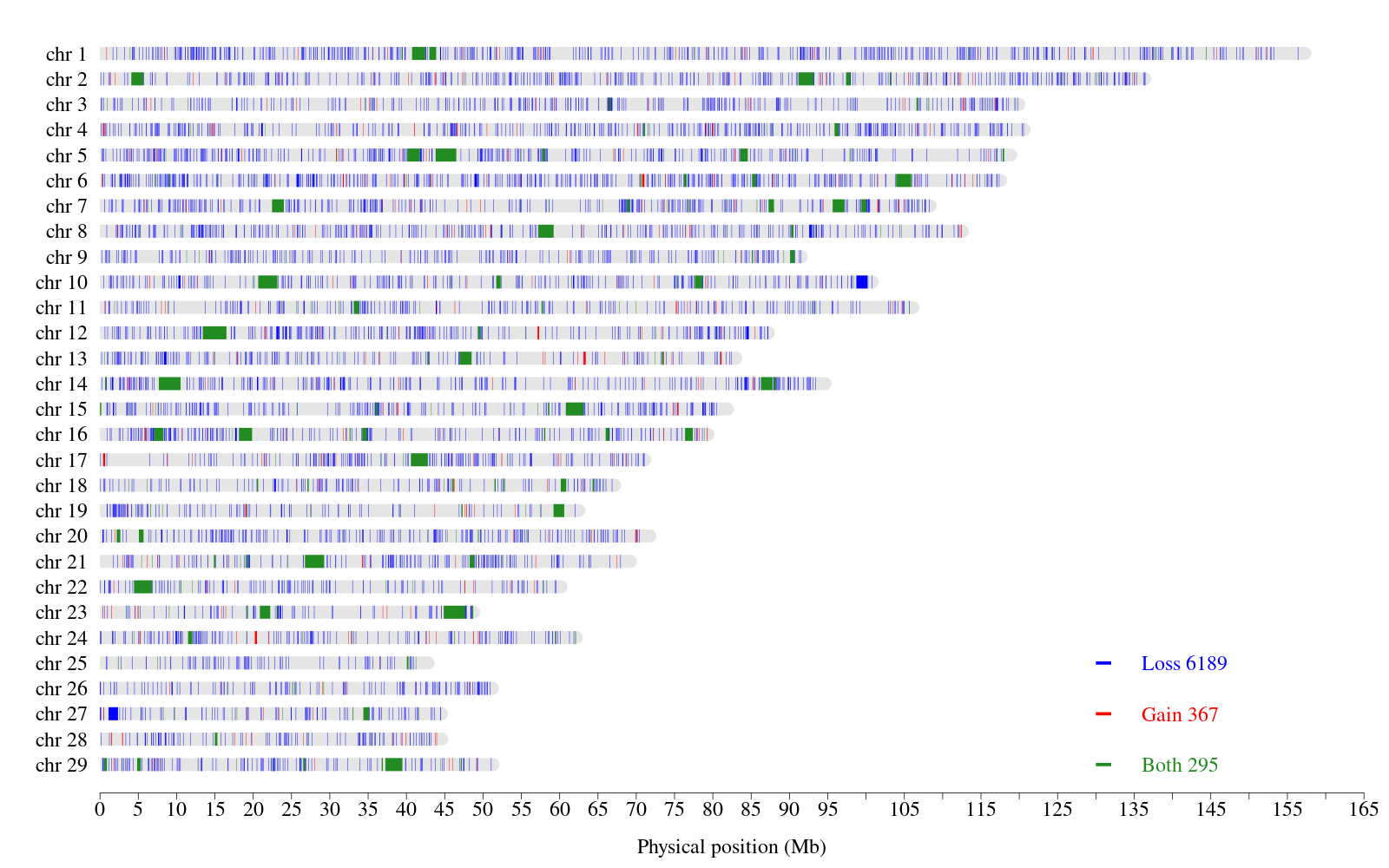
Supplementary Figure 49: Global CNVR with variable SV calling parameters

Imprecise SV included

Lower cut-off length of 50 bp applied after merging Manta and Lumpy SV

Medium SV filters (PE = SR = 3)

Upper cut-off length: 3 Mb


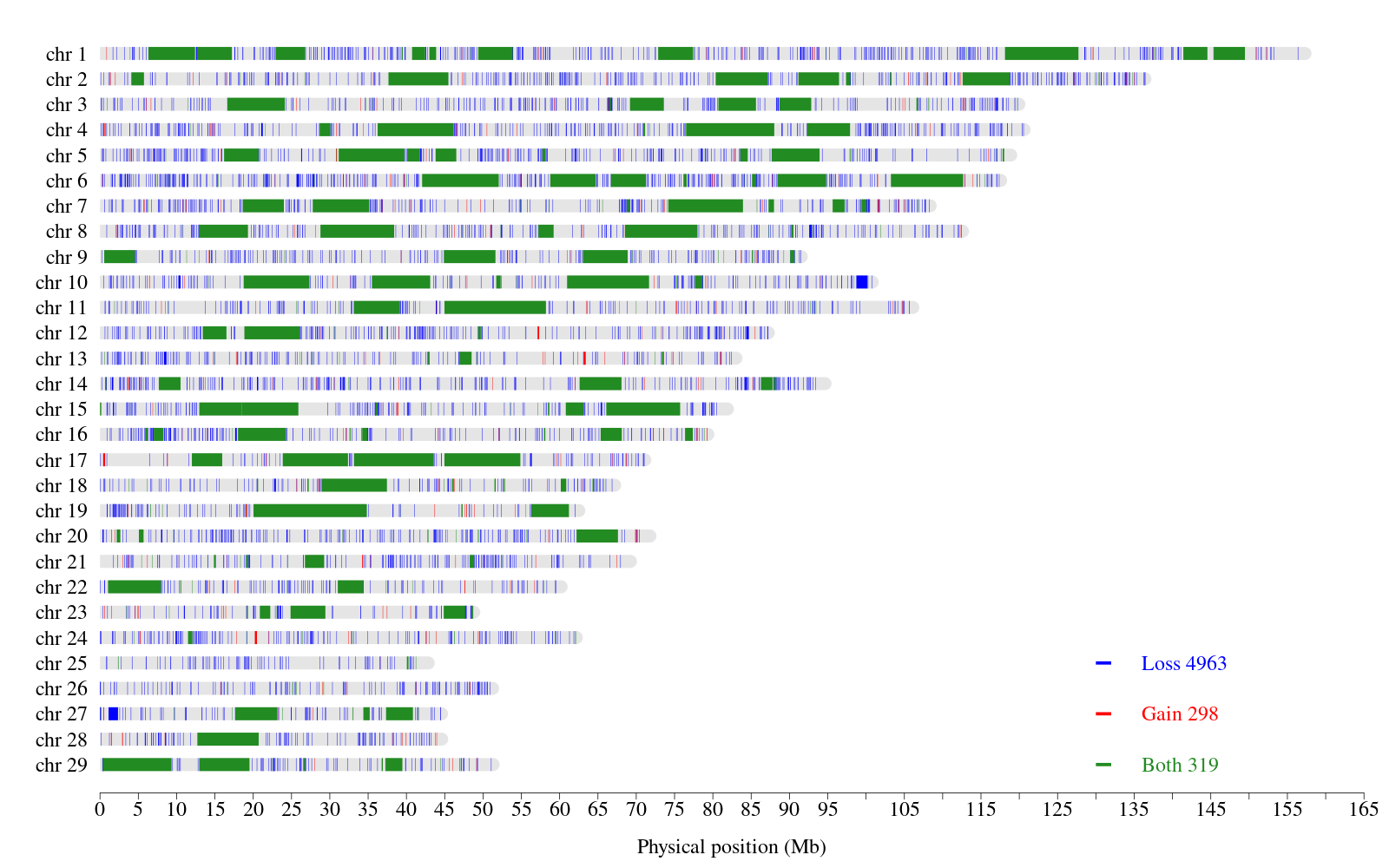
Supplementary Figure 50: Global CNVR with variable SV calling parameters

Imprecise SV included

Lower cut-off length of 50 bp applied after merging Manta and Lumpy SV

Medium SV filters (PE = SR = 3)

Upper cut-off length: 10 Mb


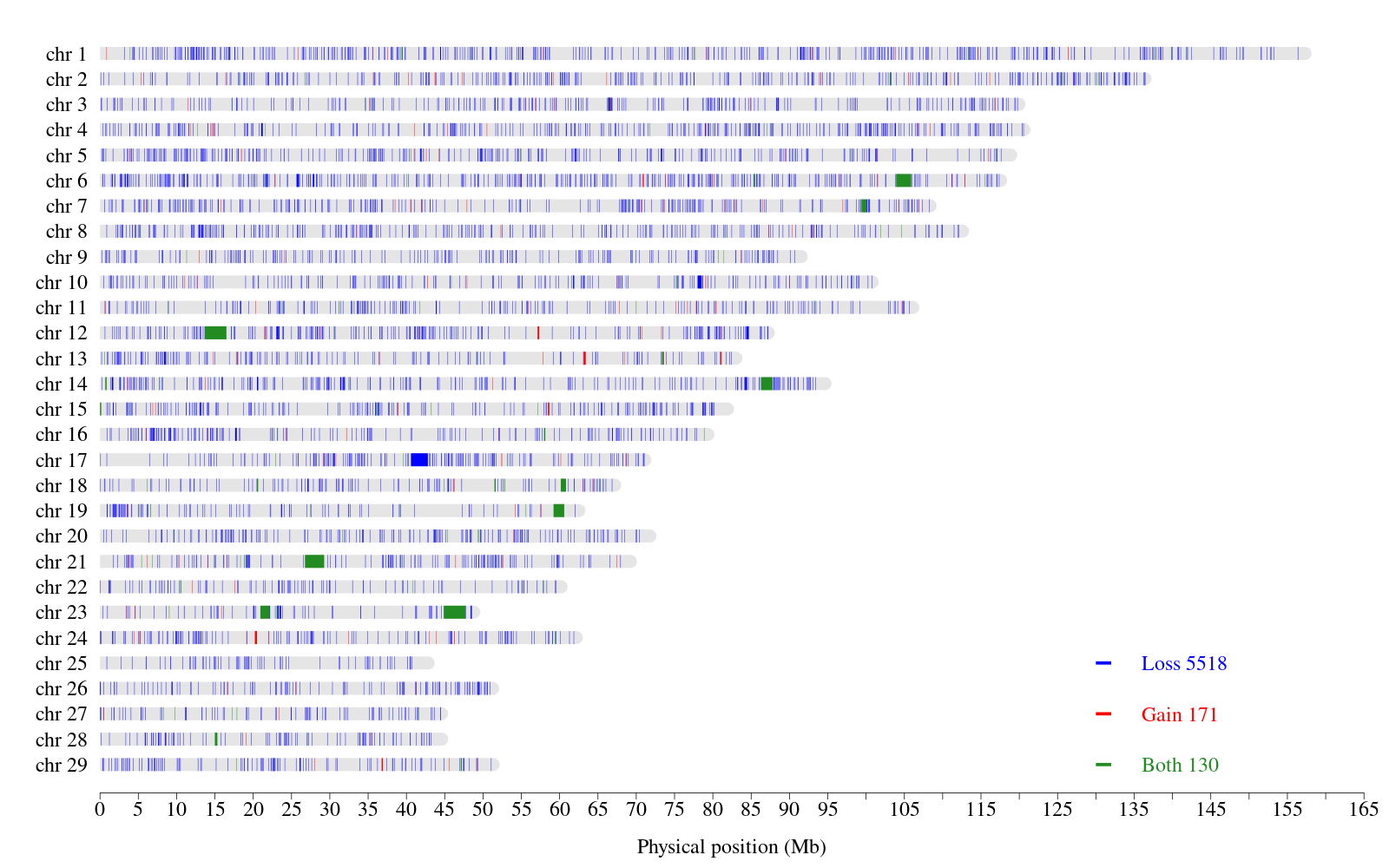
Supplementary Figure 51: Global CNVR with variable SV calling parameters

Imprecise SV included

Lower cut-off length of 50 bp applied after merging Manta and Lumpy SV

Stringent SV filters (PE = SR = 5)

Upper cut-off length: 3 Mb


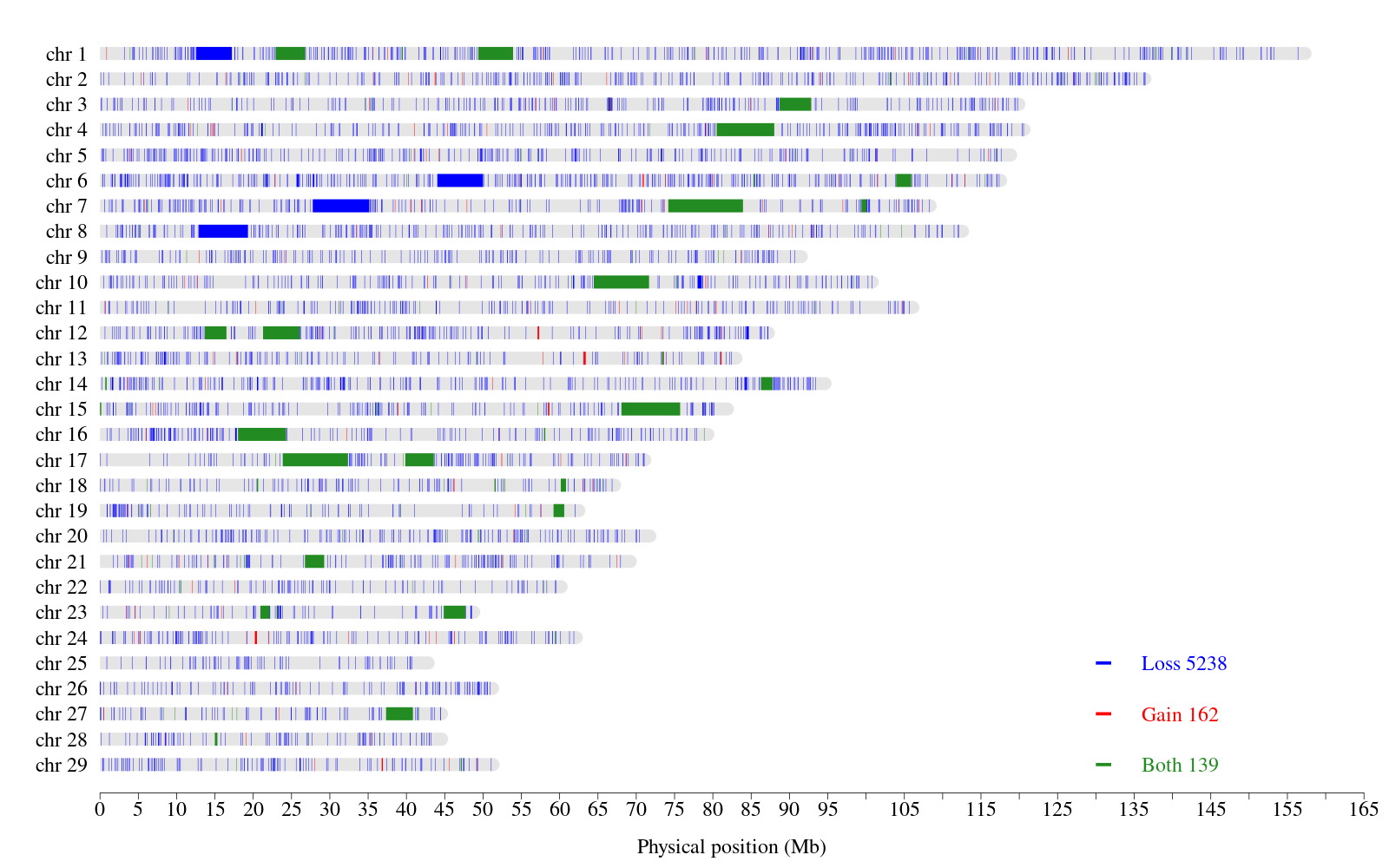
Supplementary Figure 52: Global CNVR with variable SV calling parameters

Imprecise SV included

Lower cut-off length of 50 bp applied after merging Manta and Lumpy SV

Stringent SV filters (PE = SR = 5)

Upper cut-off length: 10 Mb


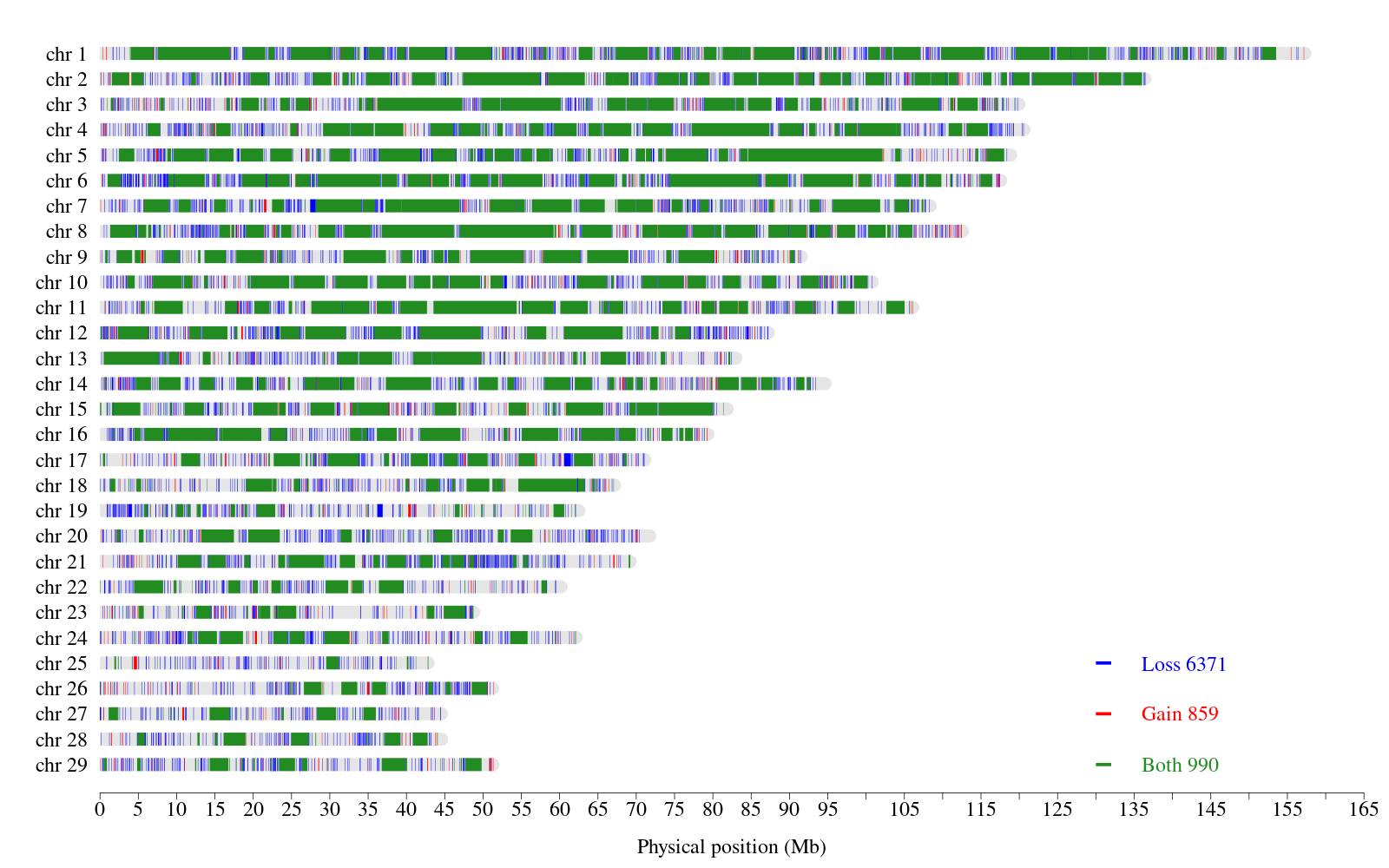
Supplementary Figure 53: Global CNVR with variable SV calling parameters

Precise SV

Lower cut-off length of 50 bp applied before merging Manta and Lumpy SV

No SV filters (PE = SR = 0)

Upper cut-off length: 3 Mb


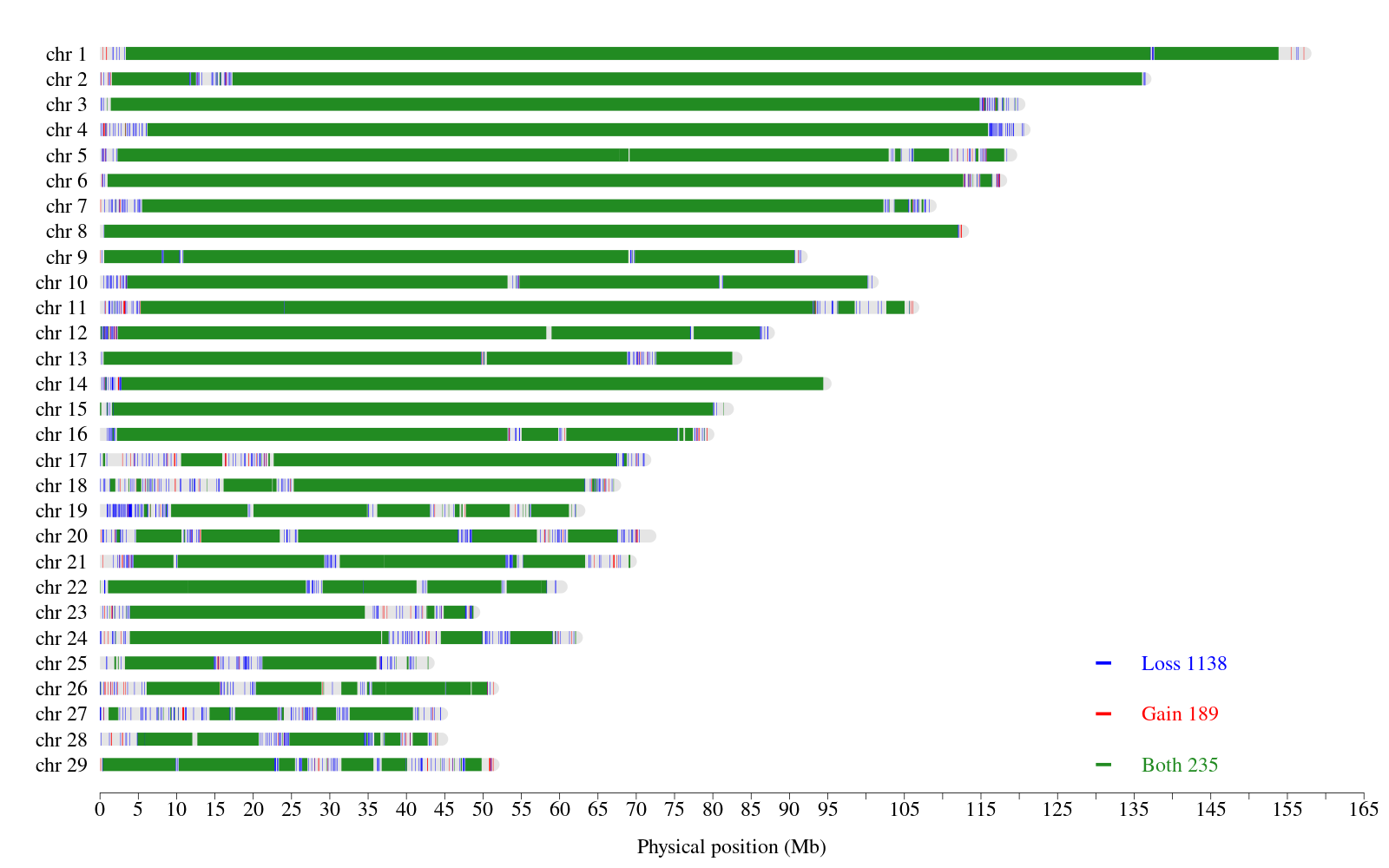
Supplementary Figure 54: Global CNVR with variable SV calling parameters

Precise SV

Lower cut-off length of 50 bp applied before merging Manta and Lumpy SV

No SV filters (PE = SR = 0)

Upper cut-off length: 10 Mb


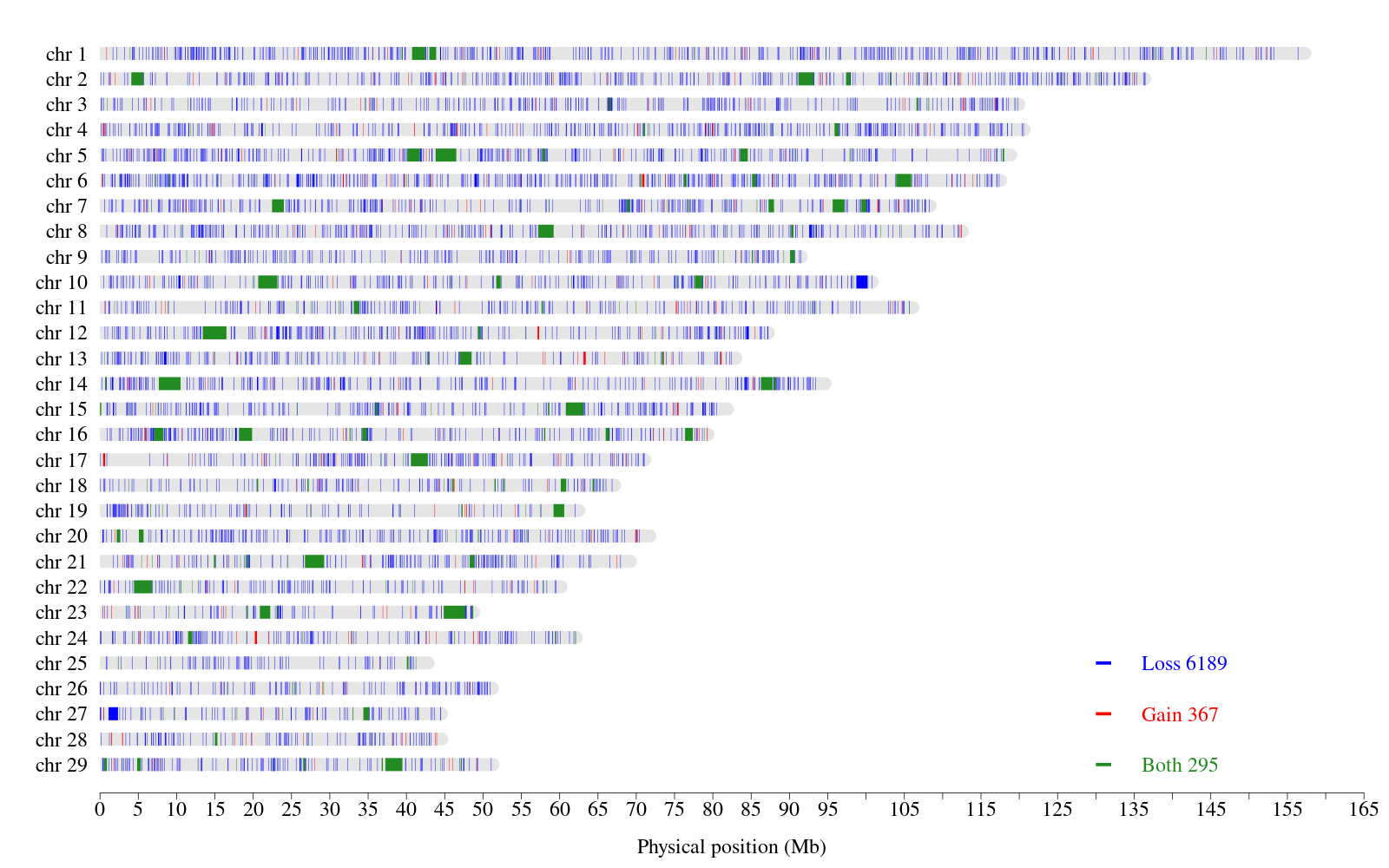
Supplementary Figure 55: Global CNVR with variable SV calling parameters

Precise SV

Lower cut-off length of 50 bp applied before merging Manta and Lumpy SV

Medium SV filters (PE = SR = 3)

Upper cut-off length: 3 Mb


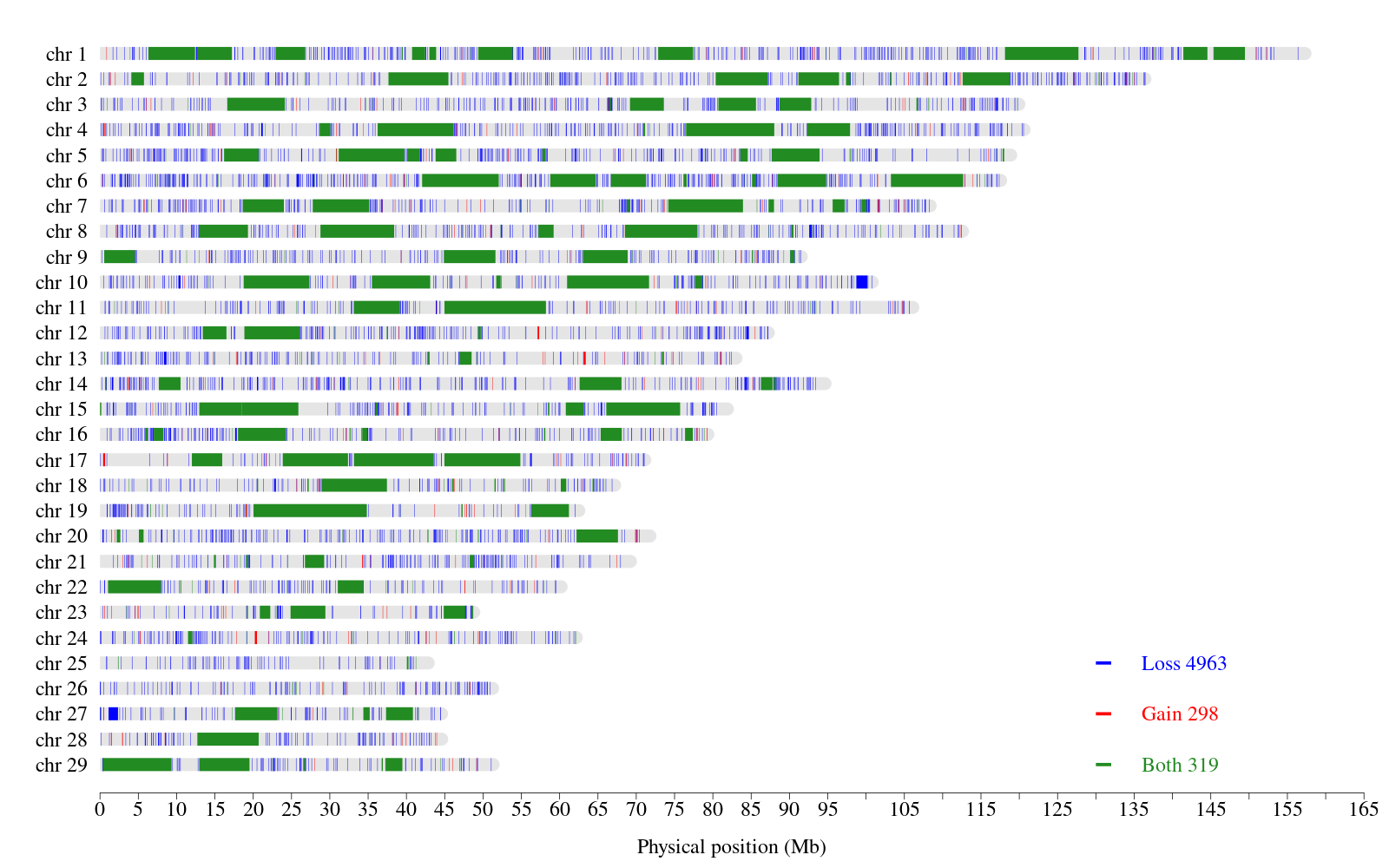
Supplementary Figure 56: Global CNVR with variable SV calling parameters

Precise SV

Lower cut-off length of 50 bp applied before merging Manta and Lumpy SV

Medium SV filters (PE = SR = 3)

Upper cut-off length: 10 Mb


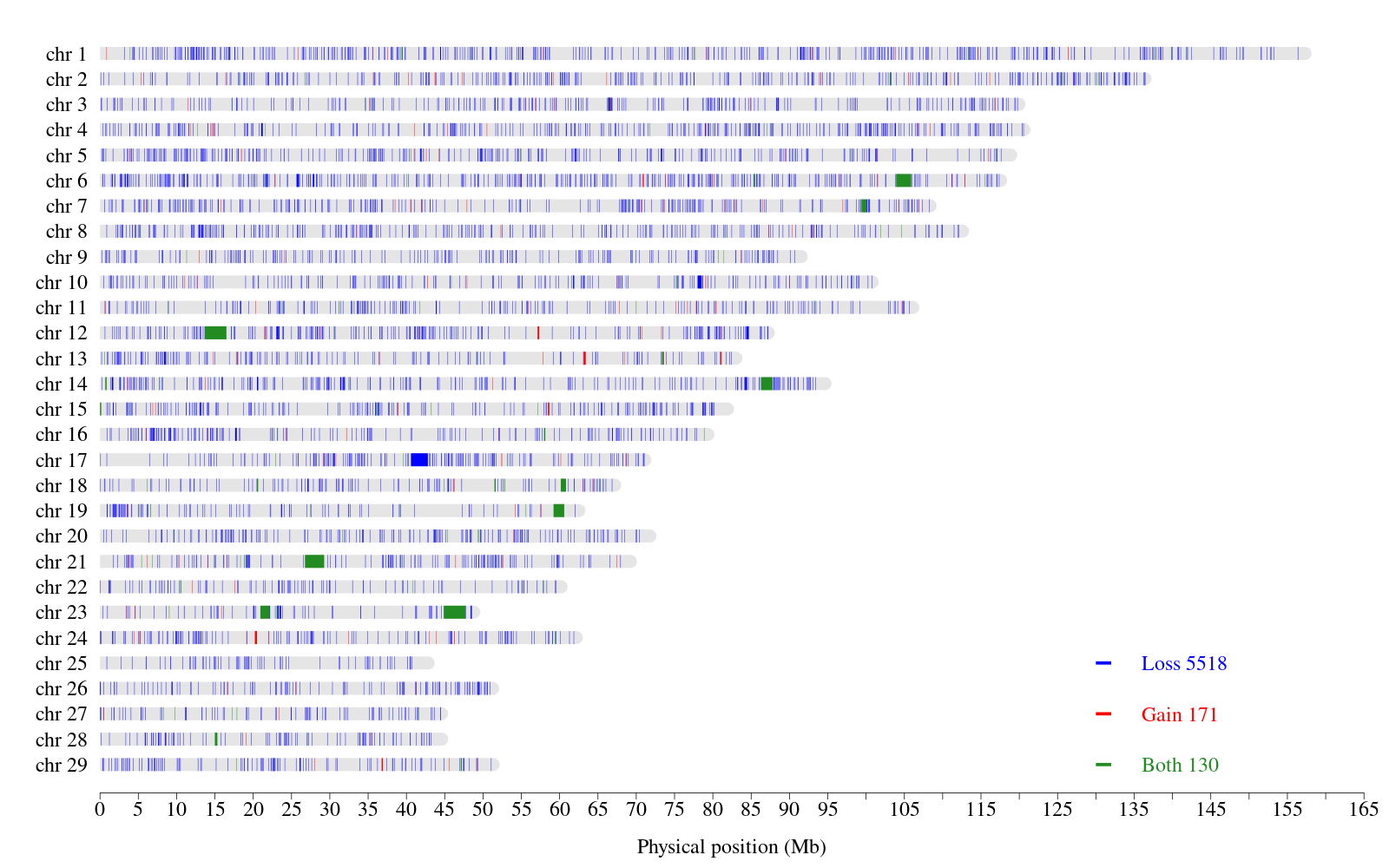
Supplementary Figure 57: Global CNVR with variable SV calling parameters

Precise SV

Lower cut-off length of 50 bp applied before merging Manta and Lumpy SV

Stringent SV filters (PE = SR = 5)

Upper cut-off length: 3 Mb


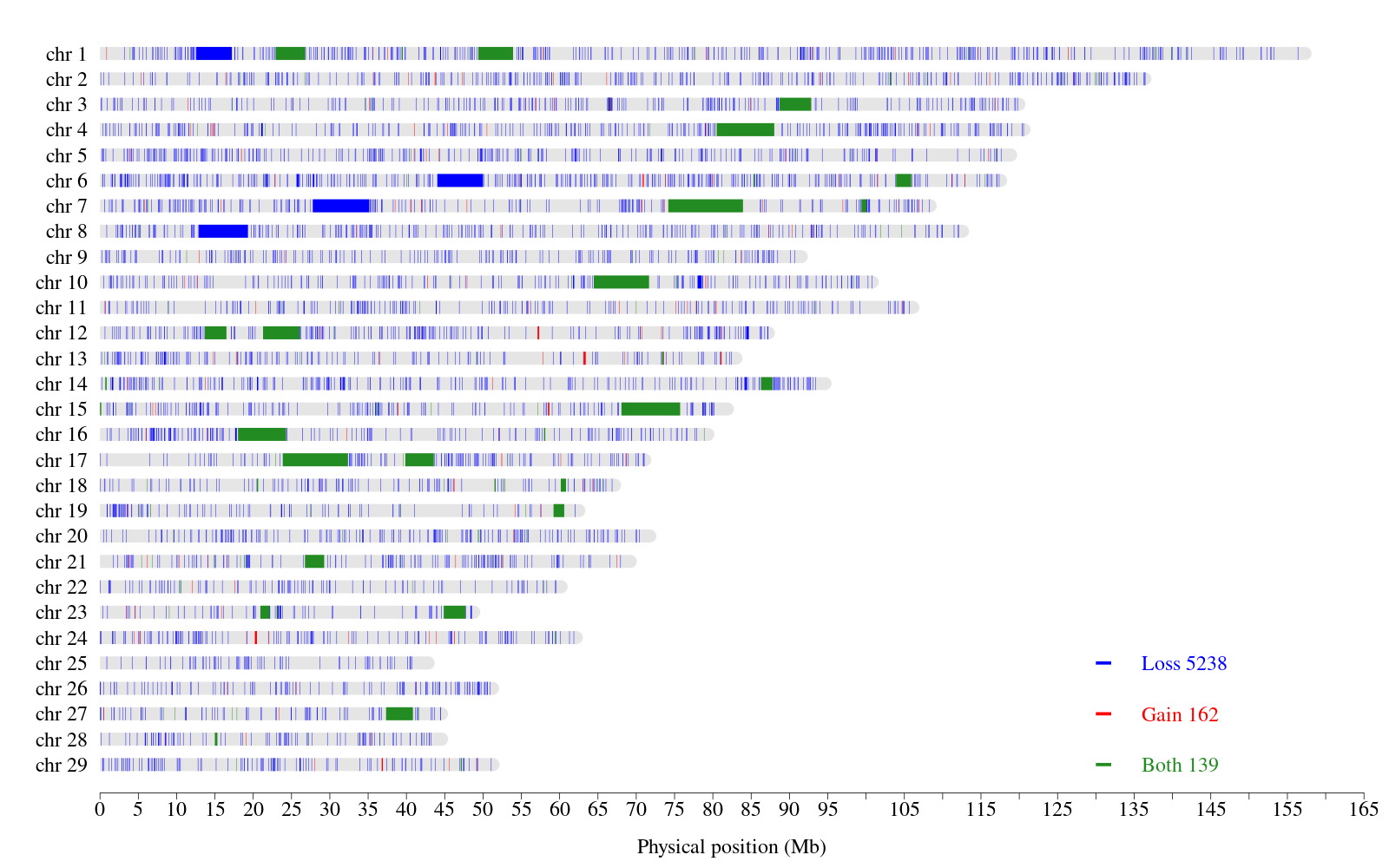
Supplementary Figure 58: Global CNVR with variable SV calling parameters

Precise SV

Lower cut-off length of 50 bp applied before merging Manta and Lumpy SV

Stringent SV filters (PE = SR = 5)

Upper cut-off length: 10 Mb


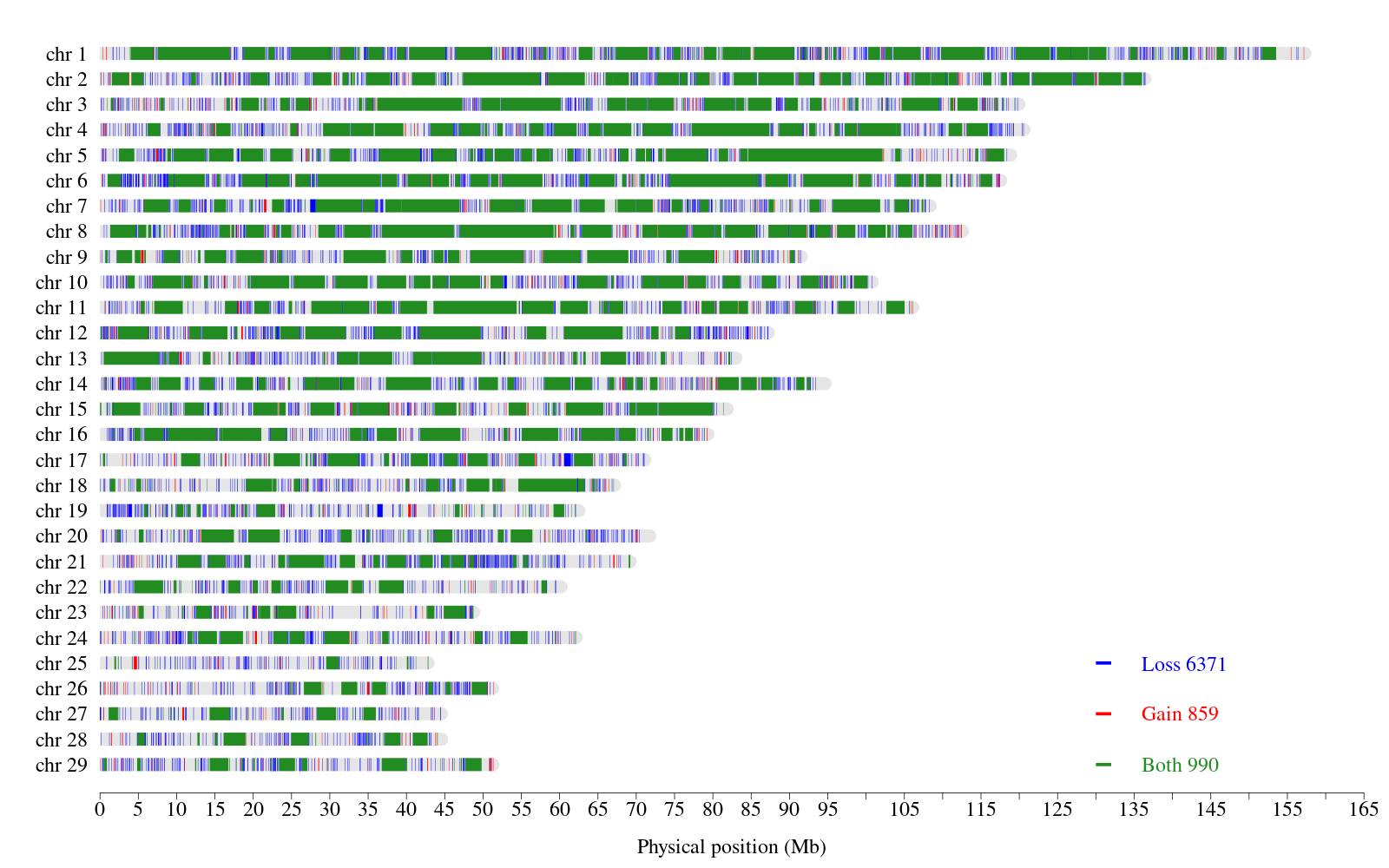
Supplementary Figure 59: Global CNVR with variable SV calling parameters

Precise SV

Lower cut-off length of 50 bp applied after merging Manta and Lumpy SV

No SV filters (PE = SR = 0)

Upper cut-off length: 3 Mb


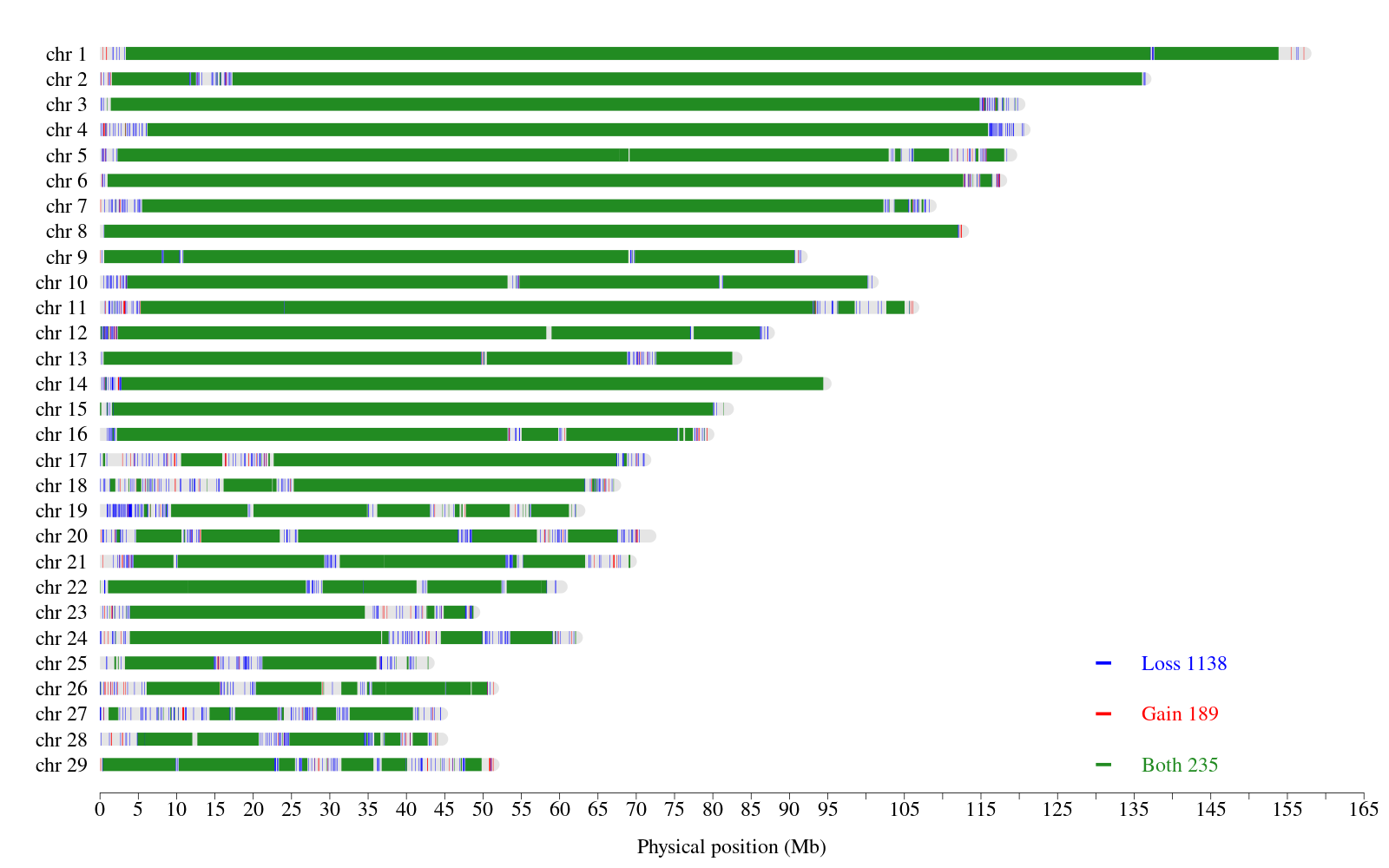
Supplementary Figure 60: Global CNVR with variable SV calling parameters

Precise SV

Lower cut-off length of 50 bp applied after merging Manta and Lumpy SV

No SV filters (PE = SR = 0)

Upper cut-off length: 10 Mb


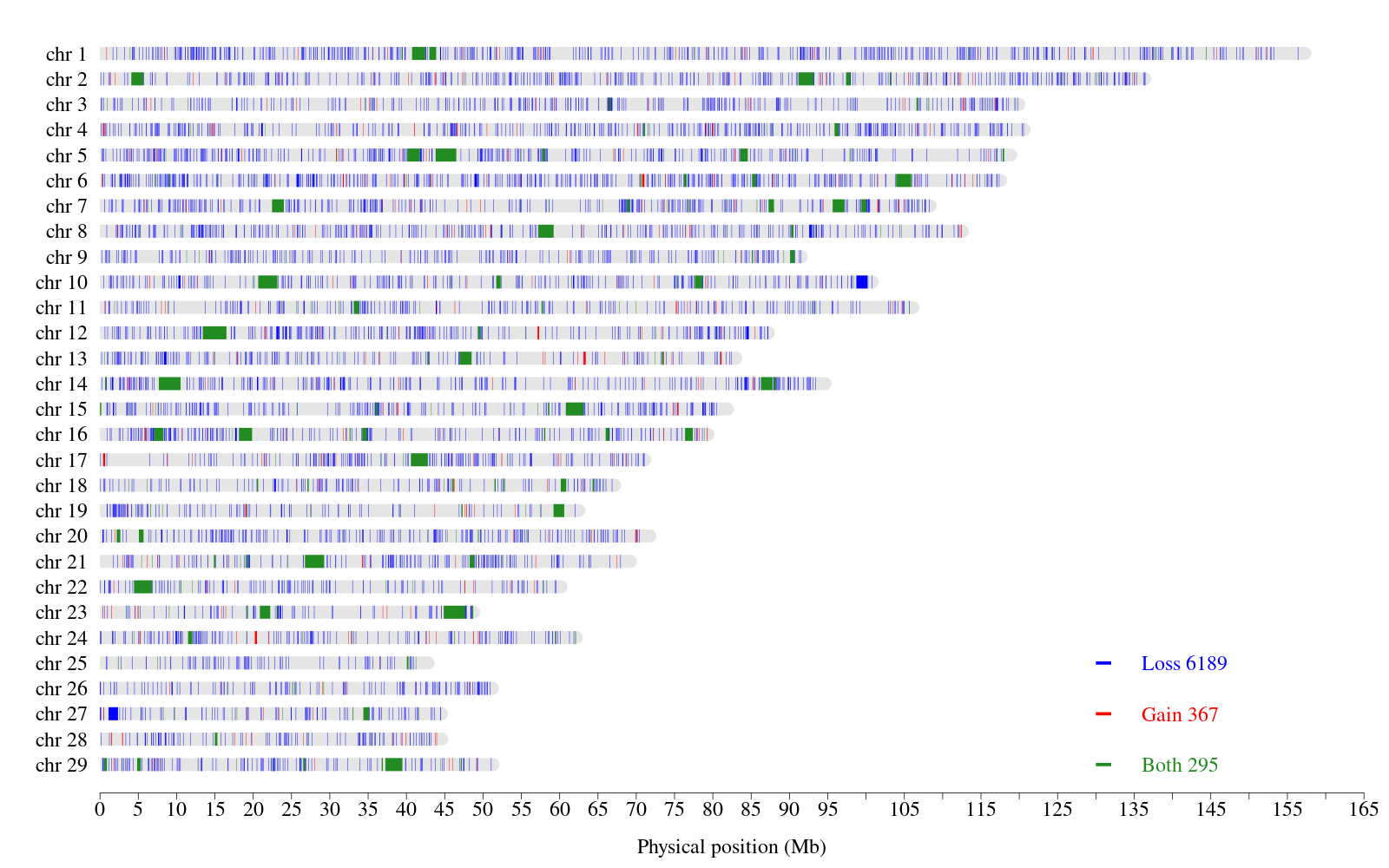
Supplementary Figure 61: Global CNVR with variable SV calling parameters

Precise SV

Lower cut-off length of 50 bp applied after merging Manta and Lumpy SV

Medium SV filters (PE = SR = 3)

Upper cut-off length: 3 Mb


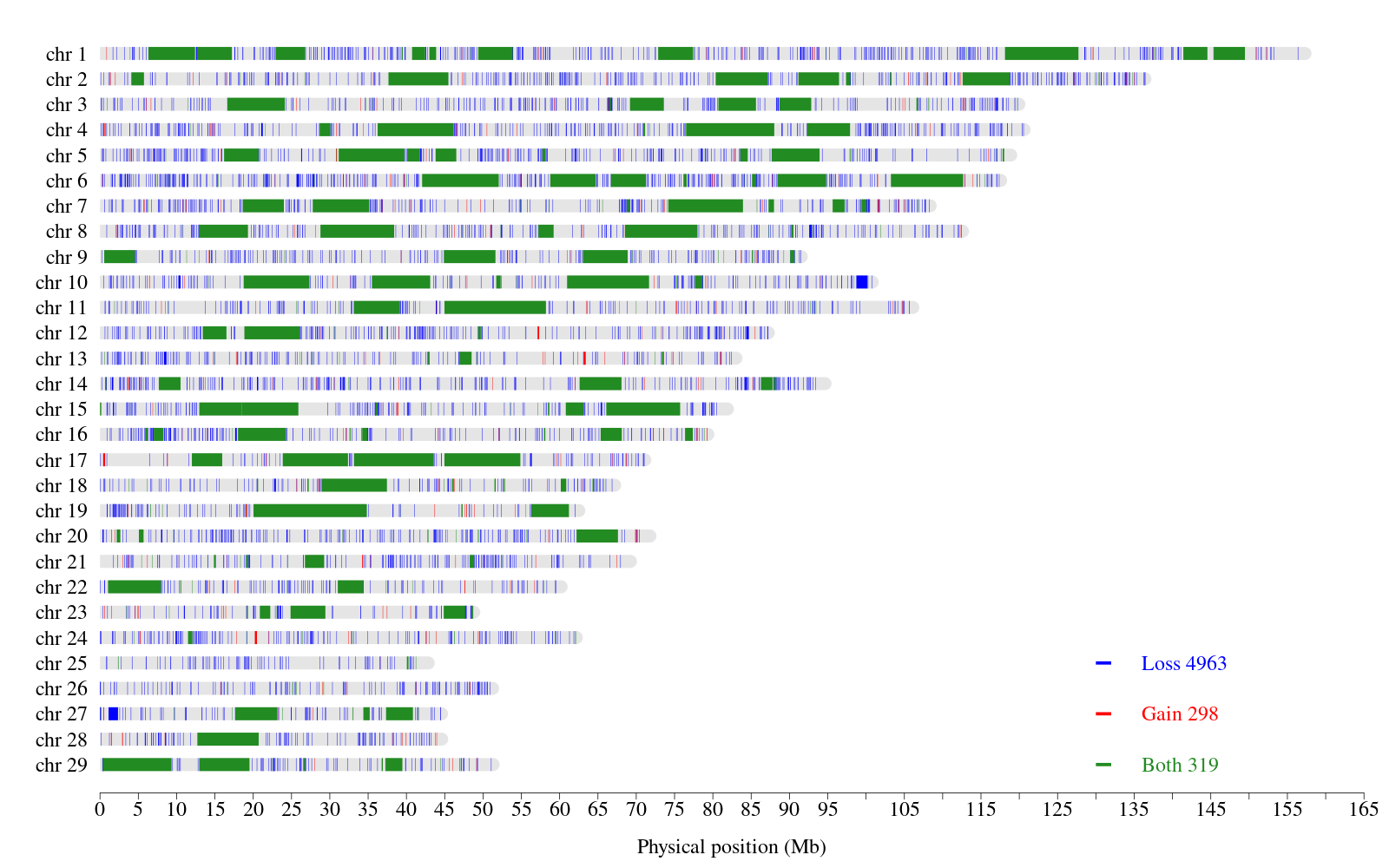
Supplementary Figure 62: Global CNVR with variable SV calling parameters

Precise SV

Lower cut-off length of 50 bp applied after merging Manta and Lumpy SV

Medium SV filters (PE = SR = 3)

Upper cut-off length: 10 Mb


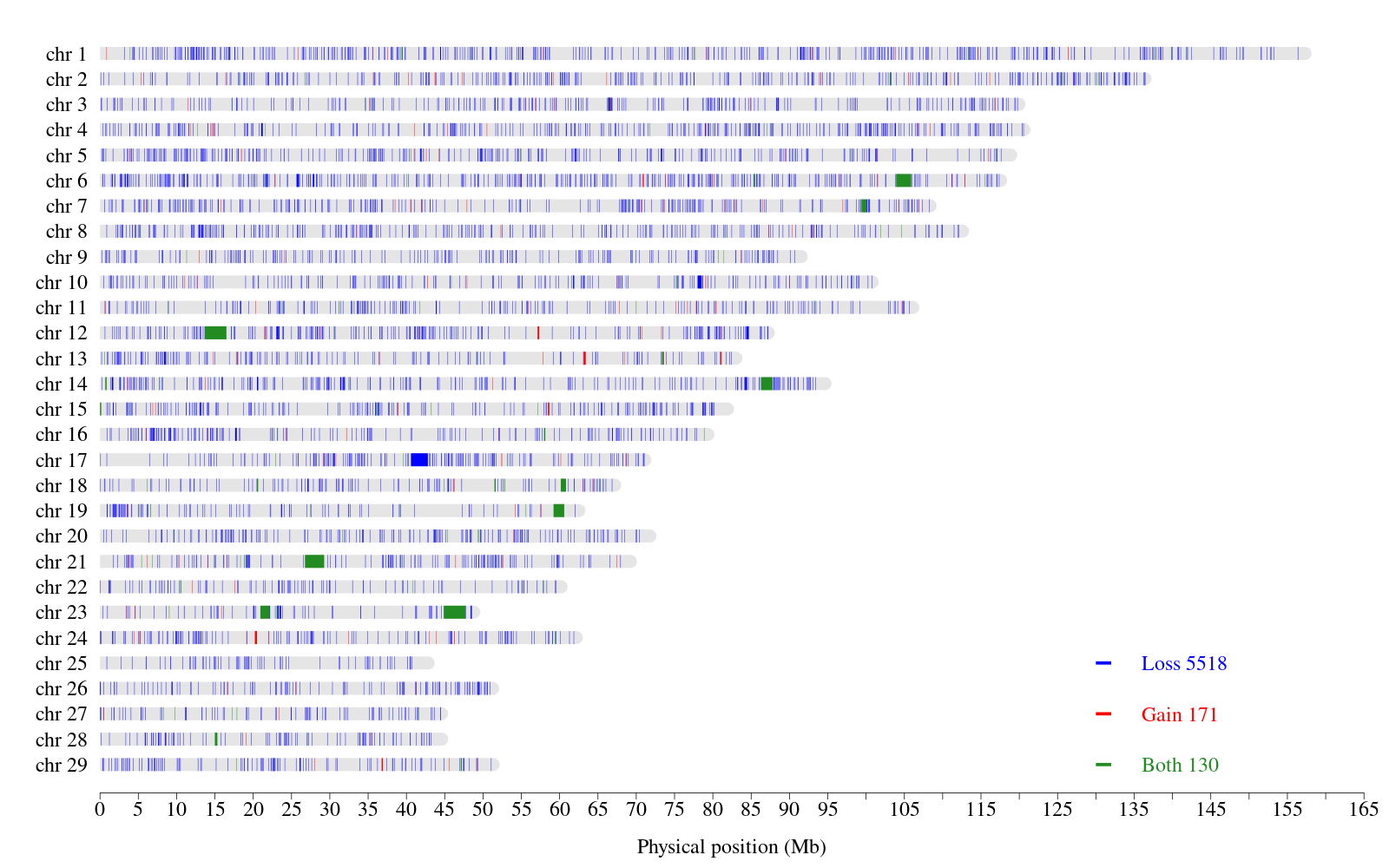
Supplementary Figure 63: Global CNVR with variable SV calling parameters

Precise SV

Lower cut-off length of 50 bp applied after merging Manta and Lumpy SV

Stringent SV filters (PE = SR = 5)

Upper cut-off length: 3 Mb


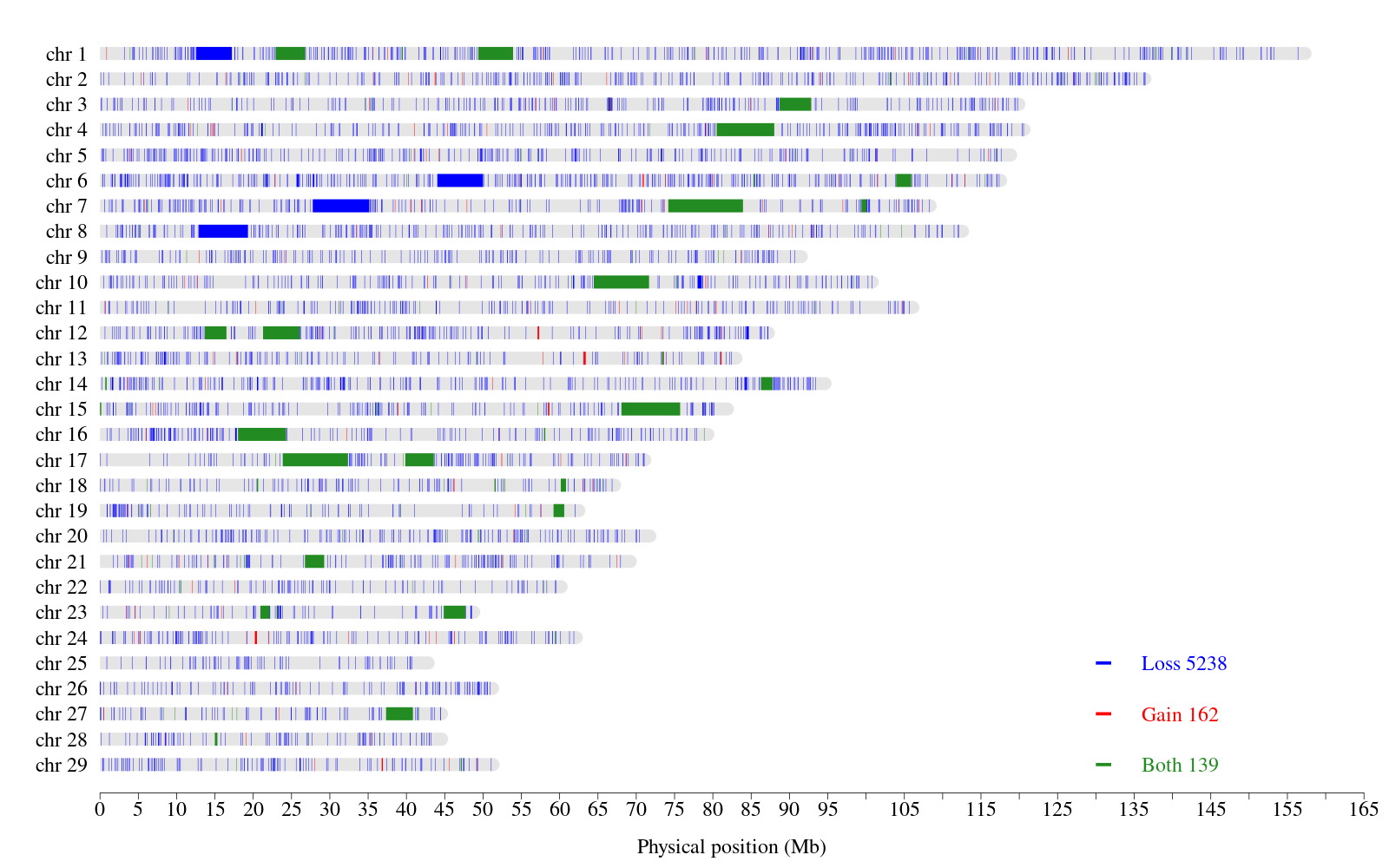
Supplementary Figure 64: Global CNVR with variable SV calling parameters

Precise SV

Lower cut-off length of 50 bp applied after merging Manta and Lumpy SV

Stringent SV filters (PE = SR = 5)

Upper cut-off length: 10 Mb
